# Global Incidence Trend of Early-Onset Obesity-Related and Non-Obesity-Related Cancers

**DOI:** 10.3390/curroncol32060324

**Published:** 2025-05-31

**Authors:** Miyu Terashima, Kota Nakayama, Satoko Ugai, Hwa-Young Lee, Yuta Tsukumo, Etsuji Suzuki, Hiroki Mizuno, Minkyo Song, Naoko Sasamoto, Ichiro Kawachi, Tomotaka Ugai

**Affiliations:** 1 Okayama University Medical School, Okayama 700-8558, Japan; 2 Department of Social and Behavioral Sciences, Harvard T.H. Chan School of Public Health, Boston, MA 02215, USA; 3 Department of Epidemiology, Harvard T.H. Chan School of Public Health, Boston, MA 02215, USAtsukumo-yuuta@mhlw.go.jp (Y.T.); hmizuno@hsph.harvard.edu (H.M.); 4 Graduate School of Public Health and Healthcare Management, The Catholic University of Korea, Seoul 06591, Republic of Korea; diana0224@catholic.ac.kr; 5 Catholic Institute for Public Health and Healthcare Management, The Catholic University of Korea, Seoul 06591, Republic of Korea; 6 Ministry of Health, Labour and Welfare, Tokyo 100-8916, Japan; 7 Department of Epidemiology, Graduate School of Medicine, Dentistry and Pharmaceutical Sciences, Okayama University, Okayama 700-8558, Japan; 8 Laboratory of Epidemiology and Population Sciences, National Institute on Aging, National Institutes of Health, Baltimore, MD 21224, USA; 9 Public Health Sciences Division, Fred Hutchinson Cancer Center, Seattle, WA 98109, USA; 10 Department of Pathology, Brigham and Women’s Hospital, Harvard Medical School, Boston, MA 02115, USA; 11 Division of Integrative Cancer Research, National Cancer Center Research Institute, Tokyo 104-0045, Japan; 12 Cancer Epidemiology Program, Dana-Farber/Harvard Cancer Center, Boston, MA 02115, USA

**Keywords:** neoplasms, etiology, risk factors, global health, young adults

## Abstract

The global rise in obesity prevalence and the incidence of early-onset cancer (diagnosed between 20 and 49 years of age) is a serious public health concern. We, therefore, evaluated the recent global trends in the incidence of early-onset obesity-related cancers and compared them to those of non-obesity-related cancers. We obtained age-standardized incidence rates of early-onset cancers diagnosed between 2000 and 2012 in 44 countries from the Cancer Incidence in Five Continents database. Using joinpoint regression models, we calculated the average annual percentage changes (AAPCs) and their corresponding 95% confidence intervals (95% CIs) for combined and individual categories of obesity-related cancers (11 and 9 cancer types in females and males, respectively) and non-obesity-related cancers (12 cancer types in both females and males). Differences in the AAPC were assessed by comparing 95% CIs, where nonoverlapping 95% CIs were considered statistically significantly different. We observed statistically significant positive AAPCs for early-onset obesity-related cancers in all available countries combined among females (global AAPC, 4.3%; 95% CI, 4.1–4.6%) and males (global AAPC, 1.4%; 95% CI, 1.2–1.7%). When analyzed by countries, we observed statistically significant positive AAPCs in 26 countries among females and 11 countries among males. AAPCs for early-onset obesity-related cancers were statistically significantly higher than those of non-obesity-related cancers in several regions, especially North America and Oceania. In conclusion, this study indicates that the incidence of early-onset obesity-related cancers exhibited a more pronounced increasing trend than non-obesity-related cancers among both sexes in many countries and regions.

## 1. Introduction

In recent decades, multiple countries, especially countries in North America, Europe, and Oceania, have reported increasing incidence of early-onset cancers (defined as cancers diagnosed between 20 and 49 years of age) in many organs, including the breast, colorectum, endometrium, esophagus, gallbladder, bile duct, head and neck, kidney, liver, bone marrow (multiple myeloma), pancreas, prostate, stomach, and thyroid [1,2,3,4,5,6,7,8,9,10,11,12,13,14]. Early-onset cancer patients stand to lose more years of their life and productivity and face more financial hardship, compared to later-onset cancer patients [15]. Furthermore, early-onset cancer survivors are at higher risk of long-term health consequences such as infertility, cardiovascular disease, and secondary cancer [16,17,18]. Therefore, a better understanding of the global incidence pattern of early-onset cancers is crucial to address this emerging global public health problem.

The obesity epidemic, which has been recognized as one of the contributing factors to the incidence of certain types of cancer, is another global public health concern [2,19]. The prevalence of obesity has dramatically increased worldwide in both adults and children/adolescents for more than a half-century [20,21]. A pooled analysis has shown that the global prevalence of obesity increased from 0.7% to 5.6% in girls and from 0.9% to 7.8% in boys during 1975–2016 [21]. The global prevalence of adult obesity also increased from 3.2% to 10.8% in males and from 6.4% to 14.9% in females during 1975–2014 [21]. However, little is known about the global trends in obesity-related early-onset cancers and whether such trends differ from those in non-obesity-related cancers.

The objectives of this study were to evaluate the recent global trends in the incidence of early-onset obesity-related cancers and to compare such trends to those of early-onset non-obesity-related cancers. We further illustrated how the early-onset cancer incidence trends differ between cancer types and how the obesity prevalence trend correlates with early-onset obesity-related cancer incidence trends by country.

## 2. Methods

### 2.1. Data Sources

We retrieved age-standardized cancer incidence rates (ASRs) of early-onset cancer (diagnosed at 20–49 years of age) from the Cancer Incidence in Five Continents database, produced by the International Agency for Research on Cancer. The original data sources and methods are described in more detail on the Cancer Incidence in Five Continents website (https://ci5.iarc.fr/) accessed on 24 October 2022. Based on data availability, we defined the study period as 2000 to 2012 for country-specific analyses and 2000 to 2010 for global and region-specific analyses to evaluate the recent trend of early-onset cancer incidence.

We retrieved ASRs of early-onset cancer in the following seven regions and 44 countries: (1) Africa: Uganda; (2) South America: Brazil, Chile, Colombia, and Ecuador; (3) Central America: Costa Rica; (4) North America: Canada and U.S.; (5) Asia: China, India, Israel, Japan, Republic of Korea, Kuwait, Thailand, and Turkey; (6) Europe: Bulgaria, Belarus, Croatia, Czechia, Denmark, Estonia, France, Germany, Iceland, Ireland, Italy, Latvia, Lithuania, Malta, Netherlands, Norway, Poland, Slovakia, Slovenia, Spain, Sweden, Switzerland, Ukraine, and U.K.; (7) Oceania: Australia and New Zealand.

According to a recent evaluation of obesity and cancer by the International Agency for Research on Cancer [22], we defined obesity-related cancer as follows: esophagus (ICD-10: C15), stomach (C16), colorectum (C18–C21), liver (C22), gallbladder and extrahepatic bile duct (C23–24), pancreas (C32), corpus uteri (C54), ovary (C56), kidney (C64), thyroid (C73), and multiple myeloma (C88 + C90). Non-obesity-related cancer was defined as follows: lip, oral cavity and pharynx (C00–14), larynx (C32), lung (C33–34), melanoma of skin (C43), Kaposi sarcoma (C46), breast (C50), cervix uteri (C53), prostate (C61), testis (C62), bladder (C67), brain and central nervous system (C70–72), Hodgkin lymphoma (C81), non-Hodgkin lymphoma (C82–86, C96), and leukemia (C91–95). Postmenopausal breast cancer is an obesity-related cancer, but most early-onset breast cancers were classified as premenopausal breast cancer, which has been inversely associated with body mass index [23,24,25,26,27,28,29]. We, therefore, classified early-onset breast cancer as non-obesity-related breast cancer in this analysis.

Based on the World Development Report 2000/2001 [30,31], we classified countries into two income groups: low-/middle-income countries and high-income countries [30,31]. We retrieved national obesity prevalence data from the National Clinical Database [32]. The age-standardized obesity prevalence in the younger population (20–49 years old) in each country was estimated referencing the Segi–Doll World standard population (1966), as it was performed for early-onset cancer incidence [33].

### 2.2. Statistical Analysis

The ASRs of early-onset cancer incidence per 100,000 person-years by cancer types, regions, countries, and sexes were calculated using direct age standardization referencing the Segi–Doll World standard population (1966) [33]. To evaluate variations in the rates over time, we calculated average ASRs during the study period for individual cancers. Using the Joinpoint Regression Program (Version 5.0.2), average annual percentage changes (AAPCs) with 95% confidence intervals (CIs) in ASRs were calculated with statistical significance corresponding to a 95% CI that does not include zero. A maximum of 2 joinpoints (3-line segments) were considered in this study. Data on “missing” or “zero” values in ASRs were excluded in this analysis.

We calculated average ASRs and AAPCs for obesity-related (11 and 9 cancer types in females and males, respectively) and non-obesity-related cancers (12 cancer types in both females and males), as well as individual cancers. We defined global AAPCs as the population-weighted averages of AAPCs in all available countries combined. We also calculated ASRs and AAPCs by regions, income levels, and countries. Differences in the AAPC between obesity-related and non-obesity-related cancers were assessed by comparing 95% CIs, where non-overlapping 95% CIs were considered as statistically significantly different.

To assess the correlation between the obesity prevalence in younger populations (20–49 years old) and early-onset cancer incidence in each country, we calculated Spearman’s rank correlation coefficient (ρ) as a measure of correlation.

We conducted the following sensitivity analyses: (1) excluding thyroid cancer from obesity-related cancers; (2) excluding stomach cancer from obesity-related cancers; (3) excluding esophageal cancer from obesity-related cancers; and (4) excluding prostate cancer from non-obesity-related cancers. We conducted analyses excluding thyroid and prostate cancers considering the effect of the increasing screening rate on the rising incidence of thyroid and prostate cancer [2,34,35]. Among esophageal and stomach cancer, only specific subtypes—esophageal adenocarcinoma and cardiac stomach cancer—are obesity-related cancers [22]; however, we were not able to classify esophageal and stomach cancers into detailed subtypes and anatomical subsites. Therefore, we conducted sensitivity analyses excluding esophageal or stomach cancers.

## 3. Results

### 3.1. Global Incidence Trends of Early-Onset Obesity-Related and Non-Obesity-Related Cancers

**Figure 1:**
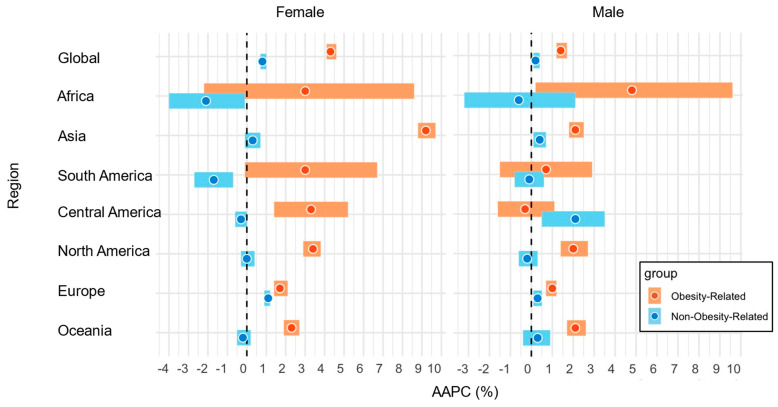
AAPCs in the incidence of early-onset obesity-related cancers and non-obesity-related cancers by regions and sexes in 2000–2010. Abbreviations: AAPC, average annual percentage change.

The global incidence of early-onset obesity-related cancers has increased both in females (ASR, 42.4; global AAPC, 4.3%; 95% CI, 4.1–4.6%) and males (ASR, 27.9; AAPC, 1.4%; 95% CI, 1.2–1.7%) (Table 1). Statistically significant positive AAPCs for obesity-related cancers were observed in Asia, Central America, North America, Europe, and Oceania among females, and in Africa, Asia, North America, Europe, and Oceania among males (Figure 1). Early-onset obesity-related cancers exhibited statistically significantly higher AAPCs compared to non-obesity-related cancers both in females [Global AAPC, 4.3% (95% CI, 4.1–4.6%) for obesity-related cancers vs. 0.8% for non-obesity-related cancers] and males [Global AAPC, 1.4% (95% CI, 1.2–1.7%) for obesity-related cancers vs. 0.2% (95% CI, 0.1–0.4%) for non-obesity-related cancers]. In Asia, while obesity-related cancers had increasing trends in both females and males, the magnitude was much higher in females [regional AAPC in females, 9.2% (95% CI, 8.8–9.7%) vs. AAPC in males, 2.1% (95% CI, 1.8–2.5%)]. Compared to non-obesity-related cancers, we observed statistically significantly higher AAPCs in early-onset obesity-related cancers in Asia, South America, Central America, North America, Europe, and Oceania among females, and in Asia, North America, Europe, and Oceania among males. When we analyzed by income levels, we observed statistically significantly higher AAPCs in early-onset obesity-related cancers in high-income countries compared to low-/middle-income countries among both females and males (Table 1).

**Figure 2:**
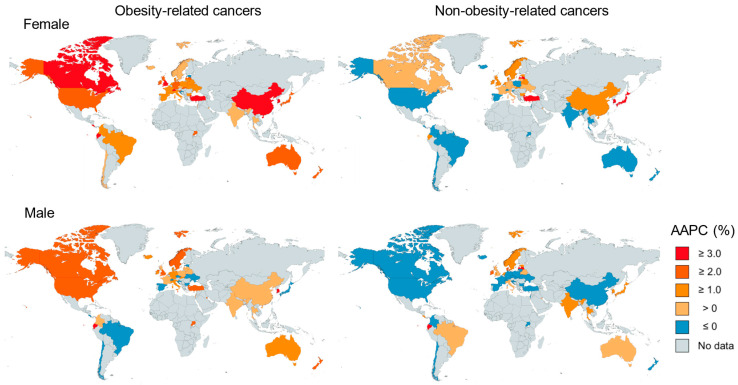
World map of the incidence trend of early-onset obesity-related cancers and non-obesity-related cancers by countries in 2000–2012.

Of the 44 countries assessed, in females, statistically significant positive AAPCs for early-onset obesity-related cancer were observed in 26 countries, ranging from 1.1% (95% CI, 0.2–2.0%; The Netherlands) to 13.2% (95% CI, 12.3–14.0%; Republic of Korea) (Figure 2, Supplementary Table S1). In males, statistically significant positive AAPCs for early-onset obesity-related cancer were observed in 11 countries, ranging from 1.2% (95% CI, 0.5–2.0%; Italy) to 4.2% (95% CI, 3.7–4.9%; Republic of Korea) (Figure 2, Supplementary Table S1). Compared to non-obesity-related cancers, we observed statistically significantly higher AAPCs for early-onset obesity-related cancers in 12 countries among females and 9 countries among males.

We further assessed the correlations between estimated obesity prevalence in younger populations and early-onset obesity-related cancer incidence for countries with statistically significant positive AAPCs for early-onset obesity-related cancers (Supplementary Figures S1 and S2). Overall, our results showed high correlations in many countries.

### 3.2. Incidence Trends of Individual Obesity-Related and Non-Obesity-Related Cancers

Among 11 obesity-related cancers in females, statistically significant positive global AAPCs were observed in six cancer types, including colorectal cancer [1.3% (0.9–1.8%)], uterine cancer [2.2% (2.0–2.4%)], kidney cancer [3.0% (2.2–3.8%)], multiple myeloma [1.9% (0.7–3.1%)], pancreas cancer [1.1% (0.2–2.1%)], and thyroid cancer [11.0% (10.5–11.5%)]. Among nine obesity-related cancers in males, statistically significant positive global AAPCs were observed in four cancer types, including colorectal cancer [1.3% (0.9–1.8%)], kidney cancer [3.3% (2.9–3.8%)], multiple myeloma [2.1% (1.3–2.8%)], and thyroid cancer [10.5% (9.7–11.4%)] (Table 2).

In females, statistically significant positive AAPCs for thyroid, uterine, colorectal, and kidney cancers were observed in 35, 14, 11, and 11 countries, respectively (Supplementary Table S2). In males, statistically significant positive AAPCs for thyroid, kidney, and colorectal cancers were observed in 24, 20, and 11 countries, respectively (Supplementary Table S3)

For non-obesity-related cancer types, we observed statistically significant positive global AAPCs in four cancer types out of 12, including breast [1.1% (0.9–1.3%)], Hodgkin lymphoma [1.4% (0.8–2.0%)], melanoma of skin [1.7% (1.3–2.0%)], and non-Hodgkin lymphoma [1.0% (0.6–1.5%)] in females, and in five cancer types out of 12, including Hodgkin lymphoma [1.0% (0.5–1.6%)], melanoma of skin [1.1% (0.6–1.6%)], and non-Hodgkin lymphoma [0.8% (0.2–1.4%)], prostate cancer [7.2% (6.5–8.0%)], and testis cancer [2.0% (1.7–2.2%)] in males (Table 3). Detailed analyses of individual non-obesity-related cancer types by countries are shown in Supplementary Tables S4 and S5.

Temporal trends in global AAPCs in early-onset obesity-related and non-obesity-related cancers are shown in Supplementary Figure S3. Temporal trends in each cancer type are shown in Supplementary Figures S4 and S5.

### 3.3. Sensitivity Analyses

To increase the robustness of our findings, we conducted several sensitivity analyses (Supplementary Table S6). Excluding esophageal, stomach, or prostate cancers did not change our findings substantially. Excluding thyroid cancer attenuated AAPCs in early-onset obesity-related cancers. However, even after excluding thyroid cancer, we still observed statistically significant differences in AAPCs between early-onset non-obesity-related cancers and non-obesity-related cancers in North America and Oceania.

## 4. Discussion

This study showed that the incidence of early-onset obesity-related cancers increased in both females and males worldwide. Notably, compared to non-obesity-related cancers, the increase in the incidence of early-onset obesity-related cancers was more prominent, especially in Asia, North America, and Oceania. For individual obesity-related cancer types, statistically significant global AAPCs were observed in colorectal, uterine, kidney, pancreas, and thyroid cancer and multiple myeloma among females, and in colorectal, kidney, and thyroid cancer and multiple myeloma among males. These cancer types considerably contributed to the increasing incidence of early-onset obesity-related cancers.

Our analyses here focused on early-onset obesity-related cancers. We defined obesity-related cancers based on evidence on the association of obesity with cancers of all ages, not specific to early-onset cancers [22]. However, emerging evidence indicates differences in risk factors between early-onset and late-onset cancers [2]. For early-onset colorectal cancer, a positive association of obesity was found in several studies [36,37,38], but evidence remains sparse for other early-onset cancer types. For breast cancer, we defined early-onset breast cancer as a non-obesity-related cancer because a higher body mass index (BMI) has been associated with reduced risk of premenopausal breast cancer. However, studies reported that a higher waist circumference was associated with reduced risk of premenopausal breast cancer after adjusting for BMI, suggesting that central adiposity and metabolic dysfunction may contribute to early-onset breast cancer risk [39]. Ideally, in future studies, we should refine the definition of early-onset obesity-related cancers after we accumulate more evidence on the association between obesity and early-onset cancers.

The rising incidence of early-onset cancers in certain organs, especially thyroid and prostate cancers, could be, at least in part, attributed to increased screening rates and/or early detection. For example, in South Korea, it was reported that the incidence of thyroid cancer increased 6.4-fold between 1999 and 2008 after the introduction of a government-funded national cancer screening program in 1999 [34,35]. About 94% of the increased cases were reported to be tumors less than 20 mm, which were mainly clinically non-significant cases [34]. To mitigate the effect of screening on early-onset cancers, we conducted analyses excluding thyroid and prostate cancers. These analyses still showed statistically significant positive AAPCs in Asia, North America, and Oceania for females, and in Africa, North America, Europe, and Oceania for males. Future studies that include cancer incidence and detailed screening data in specific areas should be conducted to decipher the impact of screening on early-onset cancer incidence.

Emerging evidence supports the hypothesis that early-life obesity could play a role in the development of early-onset cancers [2,40,41,42,43]. The exact latency period of obesity is still unknown but possibly long (maybe more than decades) and different by cancer types, which poses challenges in analyses. The childhood obesity epidemic first emerged in North America (U.S., Canada) in the 1990s, closely followed by Oceania and some parts of Europe [44,45]. In the 2000s, the epidemic spread to Central America, South America, and Asia, followed by Africa [44,45]. Although we assume that the situations were complex and different by cancer types, our findings in increased early-onset cancer incidence in North America, Oceania, and Europe between 2000 and 2010 could parallel with the childhood obesity epidemic in North America, Oceania, and Europe from the 1990s. If the childhood obesity epidemic is a significant contributor, other regions, including Central America, South America, Asia, and Africa, may follow this increasing trend. Therefore, we will need to follow future global trends in obesity and early-onset cancers.

Socioeconomic context likely plays an important role in both the obesity epidemic and the early-onset cancer epidemic on a global scale. In this study, we observed divergent trends in early-onset obesity-related cancer incidence by income levels and sexes. A more pronounced rise in obesity-related cancer incidence among females in high-income countries could be attributed to exposure to obesity and reproductive and hormonal factors, such as delayed childbirth and lower parity [46,47]. Additionally, widespread cancer screening in high-income settings may contribute to the increased detection of certain obesity-related cancers, particularly thyroid, breast, endometrial, and colorectal cancers [48]. In contrast, an emerging but less steep rise in obesity-related cancers among females in low- and middle-income countries may reflect a more recent shift toward Westernized diets, reduced physical activity, and urbanization. Interestingly, a similar steep rise was not observed among males in low- and middle-income countries. This may reflect sex-specific biological differences as well as sex differences in many environmental and lifestyle factors, healthcare-seeking behaviors, and access to healthcare [49]. Further investigations are warranted to validate our findings and clarify the potential mechanism behind the observed differences in early-onset cancer incidence by income levels and sexes.

There are several limitations in this study. First, the Cancer Incidence in Five Continents database lacks the most recent cancer incidence data in many countries. Second, cancer incidence data were not available for several regions or countries. In particular, cancer incidence data were limited in Africa, South America, and Central America. Moreover, the quality of cancer registries could be variable between countries. Third, we need to interpret our findings cautiously, especially for specific cancer types that can be affected by the increased screening rate, such as thyroid and prostate cancer, although population-level screening was not implemented in adults under age 50 in most regions except for thyroid and breast cancers. Nonetheless, we conducted several sensitivity analyses excluding these cancer types as well as analyses of individual cancer sites to better understand the global incidence patterns of early-onset cancers. Fourth, we acknowledge that incidence patterns may differ by histological and anatomical subtypes, but we were not able to compute ASRs or AAPCs by those subtypes due to the lack of data. For example, esophageal adenocarcinoma is recognized as obesity-related cancer, while esophageal squamous cell carcinoma is not. Also, stomach cancer is classified as obesity-related cancer in our study; however, cancer occurring in the lower part of the stomach is usually not considered as obesity-related cancer by the scientific community. Further studies that incorporate data on detailed histopathological and anatomical subtypes are warranted. Finally, due to the ecological study design, our study has limited values in causality. Although our study showed correlations between the global obesity prevalence trends among younger populations and rising early-onset cancer incidence trends in many countries, it is important to emphasize that cancer is a multifactorial disease affected by many factors, including many environmental exposures, behavioral patterns, and genetic susceptibility. Also, other factors, including factors related to healthcare access, screening, and cancer registry, could contribute to cancer incidence trends. Moreover, in this study, we could not consider potential confounding factors, such as smoking, alcohol consumption, hepatitis B/C infection rates, racial/ethnic demographics, socioeconomic status, healthcare access, screening rates, etc. To address potential confounding and confirm individual-level associations of obesity (including early-life obesity) with the risk of early-onset cancers, future research using individual data from large, diverse, and longitudinal cohort databases is warranted.

Our current study has notable strengths. First, this study focused on the international comparisons of early-onset obesity-related and non-obesity-related cancers across multiple cancer types and countries. This study provides important descriptive statistics warranting further investigations on how the obesity epidemic could considerably contribute to the recent rise of early-onset cancers. Second, the Cancer Incidence in Five Continents includes databases across various sources in various countries, which allowed us to conduct comprehensive analyses across organ types and regions.

## 5. Conclusions

In conclusion, this study indicates a recent global increasing incidence trend of early-onset obesity-related cancers in both sexes. Notably, the incidence of early-onset obesity-related cancers exhibited more dramatic increases compared to that of non-obesity-related cancers. Further individual-level studies are warranted to better understand the mechanisms linking lifetime obesity-related exposures to multiple early-onset cancer types.

## Figures and Tables

**Table 1:** Incidence trends of early-onset obesity-related cancers and non-obesity-related cancers by regions in 2000–2010.

Region	All Obesity-Related Cancers	All Non-Obesity-Related Cancers
	ASR/100,000 *AAPC (95% CI) ^#^	ASR/100,000 AAPC (95% CI) ^#^
Female		
Global (All countries combined) **	**42.4**	**97.2**
	**4.3 (4.1, 4.6)**	**0.8 (0.7, 1.0)**
Africa	21.6	125.5
	3.0 (−2.2, 8.6)	−2.1 (−4.0, −0.1)
Asia **	**60.4**	29.2
	**9.2 (8.8, 9.7)**	0.3 (−0.1, 0.7)
South America **	39.5	75.4
	3.0 (−0.1, 6.7)	−1.7 (−2.7, −0.7)
Central America **	**40.6**	102.4
	**3.3 (1.4, 5.2)**	−0.3 (−0.6, 0.0)
North America **	**48.3**	102.9
	**3.4 (2.9, 3.8)**	0.0 (−0.3, 0.4)
Europe **	**32.8**	**102.5**
	**1.7 (1.4, 2.1)**	**1.1 (0.9, 1.2)**
Oceania **	**38.7**	123.7
	**2.3 (1.9, 2.7)**	−0.2 (−0.5, 0.2)
Low-/Middle-Income Countries	**31.3**	68.5
	**2.7 (2.0 3.4)**	−0.1 (−0.5, 0.3)
High-Income Countries	**51.1**	**103.0**
	**7.4 (6.8, 8.1)**	**1.0 (0.8, 1.2)**
Male		
Global (All countries combined) **	**27.9**	**53.5**
	**1.4 (1.2, 1.7)**	**0.2 (0.1, 0.4)**
Africa	**19.9**	62.7
	**4.8 (0.2, 9.6)**	−0.6 (−3.2, 2.1)
Asia **	**43.4**	**31.2**
	**2.1 (1.8, 2.5)**	**0.4 (0.1, 0.7)**
South America	22.1	36.7
	0.7 (−1.5, 2.9)	−0.1 (−0.8, 0.6)
Central America	18.1	**25.9**
	−0.3 (−1.6, 1.1)	**2.1 (0.5, 3.5)**
North America **	**26.3**	62.2
	**2.0 (1.4, 2.7)**	−0.2 (−0.6, 0.3)
Europe **	**22.2**	**57.4**
	**1.0 (0.7, 1.2)**	**0.3 (0.1, 0.5)**
Oceania **	**25.9**	80.5
	**2.1 (1.7, 2.6)**	0.3 (−0.4, 0.9)
Low-/Middle-Income Countries	24.5	31.6
	−0.3 (−0.8, 0.2)	−1.1 (−1.6, −0.6)
High-Income Countries	**34.5**	38.6
	**3.0 (2.7, 3.3)**	−0.2 (−0.6, 0.1)

* Average age-standardized cancer incidence rates (ASRs) between 2000 and 2010 were calculated. ^#^ Average annual percentage changes (AAPCs) with 95% confidence intervals (CIs) in cancer incidence among 20–49-year-old adults during the period of 2000–2010 were calculated using the Joinpoint Regression Program. A maximum of two joinpoints were permitted in this analysis. ** These regions/countries showed statistically significant differences in AAPCs between obesity-related and non-obesity-related cancers. Bold numbers denote statistically significant positive AAPCs. Abbreviations: AAPC, average annual percentage change; ASR, age-standardized cancer incidence rate; CI, confidence interval.

**Table 2:** Incidence trends of individual early-onset obesity-related cancers by regions in 2000–2010.

Region	All Obesity-Related Cancers	Colorectum	Corpus Uteri	Esophagus	Gallbladder and Extrahepatic Bile Duct	Kidney	Liver	Multiple Myeloma	Ovary	Pancreas	Stomach	Thyroid
	ASR/100,000 * AAPC (95% CI) ^#^	ASR/100,000 AAPC (95% CI)	ASR/100,000 AAPC (95% CI)	ASR/100,000 AAPC (95% CI)	ASR/100,000 AAPC (95% CI)	ASR/100,000 AAPC (95% CI)	ASR/100,000 AAPC (95% CI)	ASR/100,000 AAPC (95% CI)	ASR/100,000 AAPC (95% CI)	ASR/100,000 AAPC (95% CI)	ASR/100,000 AAPC (95% CI)	ASR/100,00 AAPC (95% CI)
Female												
Global (all countries combined)	**42.4**	**7.8**	**4.6**	0.3	0.4	**2.0**	0.9	**0.6**	6.0	**1.0**	3.4	**15.4**
	**4.3 (4.1, 4.6)**	**1.3 (0.9, 1.8)**	**2.2 (2.0, 2.5)**	−0.8 (−2.5, 1.1)	−1.3 (−2.1, −0.2)	**3.0 (2.2, 3.8)**	−0.1 (−0.9, 0.8)	**1.9 (0.7, 3.1)**	−0.4 (−1.1, 0.4)	**1.1 (0.2, 2.1)**	−0.6 (−1.0, −0.2)	**11.0 (10.5, 11.5)**
Africa	21.6	3.3	2.0	**1.9**	0.4	0.4	4.1	1.0	4.7	1.5	2.3	**1.6**
	3.0 (−2.2, 8.6)	4.2 (−7.2, 17.2)	−9.8 (−24.3, 6.2)	**15.9 (4.0, 29.6)**	4.3 (−1.7, 10.6)	3.3 (−12.9, 25.1)	10.9 (−0.1, 23.7)	16.9 (−1.5, 40.0)	0.4 (−6.7, 7.3)	3.7 (−31.3, 63.0)	−16.5 (−29.5, −1.1)	**17.9 (3.3, 35.1)**
Asia	**60.4**	**7.9**	**4.2**	0.3	0.7	**1.3**	2.1	0.3	**5.7**	0.8	8.2	**29.0**
	**9.2 (8.8, 9.7)**	**1.8 (0.6, 3.0)**	**4.8 (3.6, 5.9)**	0.2 (−3.1, 3.6)	−1.9 (−4.0, 0.2)	**5.2 (2.3, 8.1)**	−2.8 (−4.3, −1.2)	1.5 (−1.1, 4.1)	**1.5 (0.5, 2.7)**	0.5 (−1.6, 2.7)	−0.3 (−0.9, 0.3)	**20.3 (18.8, 21.9)**
South America	39.5	6.5	2.2	0.2	1.5	1.3	0.7	0.4	5.3	0.8	5.3	**15.2**
	3.0 (−0.1, 6.7)	4.5 (−1.8, 11.4)	−0.8 (−3.2, 2.6)	0.5 (−19.1, 24.9)	−3.4 (−8.4, 1.7)	1.1 (−2.7, 6.3)	−4.4 (−12.4, 4.6)	−0.4 (−13.7, 15.7)	0.7 (−3.3, 5.0)	−4.7 (−14.7, 6.7)	1.4 (−1.2, 4.0)	**7.5 (3.8, 11.2)**
Central America	**40.6**	5.2	3.0	0.1	0.8	1.1	0.9	0.3	4.1	0.7	6.0	**18.3**
	**3.3 (1.4, 5.2)**	0.6 (−3.4, 4.8)	1.3 (−3.3, 6.2)	8.0 (−10.5, 28.5)	−3.3 (−10.4, 4.4)	−0.4 (−7.2, 7.0)	4.2 (−3.2, 12.2)	−8.3 (−16.4, 0.7)	−0.9 (−3.0, 1.4)	−0.9 (−6.0, 4.4)	−2.2 (−4.6, 0.2)	**8.2 (6.5, 10.0)**
North America	**48.3**	**8.6**	**7.0**	0.2	0.4	**2.7**	0.6	0.7	5.8	**1.2**	**1.4**	**19.7**
	**3.4 (2.9, 3.8)**	**1.8 (1.2, 2.4)**	**2.5 (1.8, 3.1)**	−2.7 (−7.2, 2.0)	1.2 (−1.1, 3.5)	**2.8 (1.5, 4.2)**	2.8 (−0.1, 5.8)	2.2 (−1.0, 5.6)	0.1 (−1.2, 1.4)	**2.6 (0.7, 4.7)**	**1.4 (0.3, 2.6)**	**6.0 (5.7, 6.3)**
Europe	**32.8**	**7.2**	**4.1**	0.4	0.3	**2.0**	**0.5**	0.6	6.5	1.1	2.1	**7.9**
	**1.7 (1.4, 2.1)**	**0.9 (0.5, 1.3)**	**1.2 (0.7, 2.0)**	−0.7 (−2.6, 1.3)	−1.6 (−2.3, −0.4)	**2.7 (2.0, 3.5)**	**2.6 (1.3, 4.0)**	1.2 (−0.1, 3.4)	−1.2 (−1.9, −0.6)	0.9 (−0.3, 2.1)	−1.8 (−2.5, −1.1)	**6.0 (5.4, 6.5)**
Oceania	**38.7**	10.6	**4.1**	0.2	0.3	**2.4**	0.6	0.9	4.6	1.0	1.5	**12.5**
	**2.3 (1.9, 2.7)**	1.1 (0.0, 2.3)	**2.8 (1.4, 4.4)**	0.0 (−5.3, 5.7)	0.1 (−3.8, 4.2)	**2.5 (0.3, 4.6)**	2.3 (−3.5, 8.6)	3.1 (−0.5, 6.9)	0.2 (−1.5, 2.0)	2.0 (−0.6, 4.6)	−0.4 (−4.1, 3.5)	**4.3 (3.8, 4.8)**
Male												
Global (all countries combined)	**27.9**	**8.6**		1.3	0.4	**3.7**	3.5	**0.8**		1.6	4.6	**3.4**
	**1.4 (1.2, 1.7)**	**1.3 (0.9, 1.8)**		−1.5 (−2.2, −0.8)	0.3 (−0.5, 1.1)	**3.3 (2.9, 3.8)**	−1.7 (−2.0, −1.4)	**2.1 (1.3, 2.8)**		−0.3 (−0.8, 0.5)	−1.9 (−2.2, −1.6)	**10.5 (9.7, 11.4)**
Africa	**19.9**	3.9		**5.8**	0.2	0.2	6.0	0.8		1.1	2.2	0.6
	**4.8 (0.2, 9.6)**	−1.4 (−9.8, 8.1)		**8.7 (5.0, 12.5)**	NA	−11.3 (−33.2, 21.1)	8.7 (0.0, 19.1)	−0.8 (−19.6, 22.6)		5.0 (−10.7, 23.2)	−5.6 (−21.5, 13.4)	−2.0 (−23.1, 23.5)
Asia	**43.4**	**9.4**		1.1	0.7	**2.7**	10.9	0.5		1.5	11.0	**5.6**
	**2.1 (1.8, 2.5)**	**2.8 (1.9, 3.6)**		−3.5 (−6.3, −0.6)	1.2 (−1.1, 3.5)	**5.5 (4.9, 6.2)**	−2.9 (−3.3, −2.3)	0.8 (−0.9, 2.6)		0.2 (−1.6, 2.2)	−1.7 (−2.3, −1.1)	**23.5 (21.1, 26.0)**
South America	22.1	**5.8**		1.1	0.8	2.0	1.0	0.9		0.9	7.2	**2.6**
	0.7 (−1.5, 2.9)	**3.0 (0.1, 6.1)**		−7.9 (−12.0, −3.6)	−3.4 (−12.2, 6.3)	2.2 (−6.3, 11.5)	3.5 (−12.8, 23.5)	1.8 (−17.1, 25.6)		5.3 (−0.7, 11.9)	−2.6 (−5.4, 0.2)	**5.8 (2.1, 9.8)**
Central America	18.1	**4.8**		0.2	0.4	1.4	1.3	0.3		0.9	6.3	**2.6**
	−0.3 (−1.6, 1.1)	**3.3 (0.7, 5.9)**		−7.4 (−29.4, 18.4)	−12.4 (−26.3, 4.5)	−1.2 (−10.2, 8.5)	−5.4 (−14.6, 4.7)	−7.2 (−15.8, 2.4)		−1.9 (−6.5, 3.0)	−3.4 (−5.5, −1.2)	**10.5 (3.1, 18.5)**
North America	**26.3**	**9.6**		0.9	0.4	**4.6**	2.0	**1.0**		1.5	1.9	**4.4**
	**2.0 (1.4, 2.7)**	**1.5 (1.0, 2.0)**		0.4 (−1.9, 2.7)	1.3 (−0.4, 3.7)	**3.6 (2.2, 4.9)**	−2.0 (−3.4, −0.5)	**2.7 (0.8, 4.0)**		0.1 (−1.0, 1.5)	0.1 (−1.6, 2.0)	**5.2 (3.8, 6.5)**
Europe	**22.2**	**7.7**		1.6	0.3	**3.9**	**1.2**	0.9		1.7	2.9	**2.0**
	**1.0 (0.7, 1.2)**	**0.7 (0.2, 1.2)**		−1.5 (−2.8, −0.2)	0.2 (−1.1, 1.6)	**2.7 (2.1, 3.0)**	**3.1 (1.6, 4.3)**	1.8 (−0.1, 3.7)		−0.6 (−1.4, 0.4)	−2.4 (−2.9, −2.0)	**5.3 (4.5, 6.5)**
Oceania	**25.9**	**10.5**		1.1	0.3	**4.2**	1.7	1.3		1.4	2.0	**3.3**
	**2.1 (1.7, 2.6)**	**1.3 (0.9, 1.8)**		0.6 (−1.7, 3.0)	−2.9 (−9.5, 4.2)	**4.5 (1.2, 8.0)**	2.6 (−0.5, 6.0)	1.8 (−1.7, 5.4)		2.4 (−1.4, 5.9)	−0.3 (−2.0, 1.4)	**5.2 (2.9, 7.6)**

* Average age-standardized cancer incidence rates (ASRs) between 2000 and 2010 were calculated. ^#^ Average annual percentage changes (AAPCs) with 95% confidence intervals (CIs) in cancer incidence among 20–49-year-old adults during the period of 2000–2010 were calculated using the Joinpoint Regression Program. A maximum of two joinpoints were permitted in this analysis. Bold numbers denote statistically significant positive AAPCs. Abbreviations: AAPC, average annual percentage change; ASR, age-standardized cancer incidence rate; CI, confidence interval.

**Table 3:** Incidence trends of individual early-onset non-obesity-related cancers by regions in 2000–2010.

Region	All Non-Obesity-Related Cancers	Bladder	Brain CNS	Breast	Cervix Uteri	Hodgkin Lymphoma	Kaposi Sarcoma	Larynx	Leukemia	Lips, Oral Cavity, and Pharynx	Lung	Melanoma of Skin	Non-Hodgkin Lymphoma	Prostate	Testis
	ASR/100,000 * AAPC (95% CI) ^#^	ASR/100,000 AAPC (95% CI)	ASR/100,000 AAPC (95% CI)	ASR/100,000 AAPC (95% CI)	ASR/100,000 AAPC (95% CI)	ASR/100,000 AAPC (95% CI)	ASR/100,000 AAPC (95% CI)	ASR/100,000 AAPC (95% CI)	ASR/100,000 AAPC (95% CI)	ASR/100,000 AAPC (95% CI)	ASR/100,000 AAPC (95% CI)	ASR/100,000 AAPC (95% CI)	ASR/100,000 AAPC (95% CI)	ASR/100,000 AAPC (95% CI)	ASR/100,000 AAPC (95% CI)
Female															
Global (all countries combined)	**97.2**	0.8	3.4	**52.8**	12.7	**2.5**	0.2	0.2	2.9	2.3	4.6	**10.7**	**4.1**		
	**0.8 (0.7, 1.0)**	−0.8 (−1.6, 0.1)	0.2 (−0.2, 0.7)	**1.1 (0.9, 1.3)**	0.0 (−0.4, 0.3)	**1.4 (0.8, 2.0)**	−0.3 (−1.5, 0.8)	−2.2 (−4.4, 0.1)	0.4 (−0.3, 1.2)	0.5 (−0.1, 1.1)	−0.7 (−1.5, 0.1)	**1.7 (1.3, 2.0)**	**1.0 (0.6, 1.5)**		
Africa	125.5	0.6	0.9	25.8	49.3	1.7	36.2	0.4	**1.0**	2.6	1.6	**0.7**	5.5		
	−2.1 (−4.0, −0.1)	−4.0 (−23.5, 20.6)	2.9 (−19.7, 31.8)	0.1 (−4.1, 7.4)	−1.8 (−4.7, 1.3)	−5.6 (−25.3, 20.1)	−6.2 (−11.0, −1.2)	−4.4 (−39.2, 47.5)	**15.4 (1.5, 31.8)**	−5.8 (−14.8, 3.9)	−13.7 (−32.6, 10.9)	**27.4 (6.5, 52.0)**	−1.5 (−9.5, 7.2)		
Asia	29.2	0.5	2.1	**43.6**	12.0	**0.9**	0.0	0.1	2.7	2.6	**3.9**	1.0	**3.3**		
	0.3 (−0.1, 0.7)	0.7 (−1.2, 3.1)	0.3 (−0.5, 1.0)	**3.0 (2.6, 3.4)**	−1.0 (−2.1, 0.1)	**3.9 (0.1, 7.9)**	3.9 (−12.0, 21.9)	−1.0 (−8.4, 7.0)	0.5 (−0.5, 1.5)	−0.4 (−1.2, 0.5)	**1.6 (0.7, 2.5)**	1.0 (−0.4, 2.4)	**2.7 (0.9, 4.6)**		
South America	75.4	0.6	3.0	35.0	21.1	1.3	0.2	0.2	3.7	**1.7**	**2.0**	2.2	4.5		
	−1.7 (−2.7, −0.7)	−4.1 (−8.4, 0.5)	−0.8 (−5.7, 4.4)	−1.0 (−2.1, 0.1)	−5.2 (−7.3, −3.0)	−0.4 (−10.3, 11.0)	−0.1 (−8.4, 8.7)	−0.8 (−22.4, 28.2)	−2.0 (−5.9, 2.0)	**5.3 (1.0, 10.0)**	**3.5 (0.1, 7.1)**	−0.6 (−5.0, 4.2)	0.4 (−4.0, 5.0)		
Central America	102.4	0.4	1.9	28.3	19.1	1.9	0.2	0.2	2.8	1.2	0.9	1.6	2.8		
	−0.3 (−0.6, 0.0)	−2.8 (−9.1, 6.4)	−3.7 (−10.5, 4.0)	0.6 (−1.4, 2.6)	−2.6 (−5.6, 0.6)	2.1 (−4.3, 9.0)	−4.9 (−16.3, 8.2)	7.9 (−33.3, 87.2)	−4.4 (−11.0, 2.7)	−3.4 (−10.6, 4.7)	1.1 (−9.1, 12.5)	8.1 (−0.9, 16.0)	2.6 (−3.3, 9.0)		
North America	102.9	1.0	3.1	56.6	9.5	3.3	0.0	0.2	3.2	2.4	5.8	12.1	5.5		
	0.0 (−0.3, 0.4)	−3.3 (−5.2, −1.4)	−1.3 (−4.0, 1.4)	0.2 (−0.3, 0.7)	−1.5 (−2.0, −1.0)	0.2 (−0.8, 1.2)	−7.0 (−13.8, 0.4)	−2.0 (−5.6, 1.8)	1.3 (0.0, 2.6)	0.2 (−2.1, 2.0)	−1.7 (−3.2, −0.3)	0.1 (−1.0, 1.2)	0.6 (−0.6, 1.9)		
Europe	**102.5**	1.0	**4.2**	**54.9**	14.3	**3.0**	0.1	0.2	2.7	2.0	4.7	**12.5**	3.7		
	**1.1 (0.9, 1.2)**	1.4 (0.0, 2.9)	**0.6 (0.1, 1.1)**	**0.9 (0.6, 1.3)**	0.7 (0.0, 1.4)	**1.2 (0.6, 1.9)**	6.2 (−5.7, 18.2)	−2.8 (−4.0, −1.5)	0.2 (−0.6, 0.9)	1.4 (0.0, 2.9)	−0.6 (−1.2, 0.1)	**3.3 (2.6, 4.1)**	1.2 (−0.4, 2.8)		
Oceania	123.7	0.5	3.1	**62.6**	8.6	2.8	0.0	0.1	3.5	3.0	4.3	30.1	5.0		
	−0.2 (−0.5, 0.2)	−6.1 (−11.5, −0.2)	0.6 (−1.6, 2.8)	**0.6 (0.1, 1.1)**	0.0 (−1.5, 1.6)	1.5 (−0.5, 3.5)	−12.9 (−29.3, 7.1)	1.6 (−7.9, 12.2)	0.4 (−2.4, 3.3)	−0.2 (−1.8, 1.2)	−0.6 (−1.6, 0.3)	−1.9 (−2.8, −1.0)	0.6 (−0.6, 1.9)		
Male															
Global (all countries combined)	**53.5**	2.4	4.2			**2.9**	0.7	1.2	3.8	5.5	6.6	**7.5**	**5.8**	**2.9**	**10.0**
	**0.2 (0.1, 0.4)**	−1.0 (−1.5, −0.5)	0.1 (−0.4, 0.7)			**1.0 (0.5, 1.6)**	−0.3 (−2.2, 2.5)	−5.3 (−6.0, −4.6)	0.5 (−0.2, 1.2)	−1.3 (−1.5, −1.1)	−3.5 (−3.9, −3.2)	**1.1 (0.6, 1.6)**	**0.8 (0.2, 1.4)**	**7.2 (6.5, 8.0)**	**2.0 (1.7, 2.2)**
Africa	62.7	0.8	1.0			1.6	41.8	0.8	1.6	4.5	1.6	0.9	6.7	1.4	0.5
	−0.6 (−3.2, 2.1)	−5.1 (−19.6, 12.4)	−1.2 (−19.9, 22.5)			4.8 (−5.4, 16.3)	−1.5 (−5.4, 2.7)	−2.3 (−16.1, 14.1)	18.1 (−7.8, 50.2)	−2.9 (−12.7, 8.0)	6.4 (−9.5, 25.8)	−5.7 (−25.4, 18.5)	4.9 (−7.2, 19.1)	−9.7 (−18.6, 0.3)	−8.4 (−28.1, 17.6)
Asia	**31.2**	2.1	2.6			**1.1**	**0.1**	0.8	3.4	5.6	7.3	0.9	**4.2**	**0.4**	**2.7**
	**0.4 (0.1, 0.7)**	0.1 (−1.5, 1.7)	0.0 (−1.5, 1.5)			**4.0 (3.0, 4.6)**	**12.8 (4.0, 22.6)**	−2.4 (−3.9, −0.7)	0.9 (−0.1, 2.0)	−0.9 (−1.2, −0.5)	−2.4 (−3.6, −1.1)	0.8 (−0.3, 1.9)	**1.5 (0.9, 2.2)**	**12.7 (9.0, 17.1)**	**4.9 (3.6, 6.3)**
South America	36.7	1.1	4.2			1.6	1.8	0.8	4.2	3.1	2.4	1.9	5.9	**2.8**	6.9
	−0.1 (−0.8, 0.6)	−2.7 (−10.6, 6.1)	−0.4 (−6.6, 6.4)			−0.1 (−7.6, 8.1)	−3.8 (−9.5, 2.5)	−0.7 (−9.9, 9.3)	−2.3 (−5.8, 1.5)	0.4 (−3.1, 4.1)	−1.5 (−6.7, 4.0)	−4.2 (−16.1, 9.9)	1.2 (−5.1, 7.3)	**7.4 (3.8, 11.1)**	0.7 (−1.2, 2.9)
Central America	**25.9**	0.7	2.5			2.1	0.7	0.5	3.2	1.8	1.4	1.2	**3.8**	**1.8**	**6.0**
	**2.1 (0.5, 3.5)**	0.9 (−11.6, 15.2)	1.5 (−1.8, 5.0)			1.4 (−5.6, 8.9)	−2.6 (−13.1, 9.2)	4.7 (−5.5, 16.2)	−3.1 (−12.4, 4.7)	−4.8 (−8.8, −0.5)	−0.5 (−10.2, 10.6)	1.1 (−8.7, 11.9)	**5.1 (2.2, 8.2)**	**9.1 (0.2, 19.0)**	**4.6 (2.6, 6.6)**
North America	62.2	2.8	4.2			3.8	1.5	0.7	**4.5**	4.8	5.2	8.8	8.2	**7.1**	**10.6**
	−0.2 (−0.6, 0.3)	−1.5 (−2.8, 0.0)	0.3 (−1.5, 2.1)			0.1 (−1.4, 1.5)	−3.5 (−5.6, −1.2)	−4.7 (−6.9, −2.3)	**0.6 (0.2, 1.1)**	−0.9 (−2.0, −0.2)	−4.3 (−5.6, −3.1)	−0.8 (−1.6, 0.1)	−0.5 (−0.9, 0.1)	**2.5 (0.8, 4.6)**	**1.2 (0.5, 1.9)**
Europe	**57.4**	2.6	5.0			3.5	0.4	1.7	3.6	5.5	7.4	**7.7**	**5.3**	**1.9**	**13.1**
	**0.3 (0.1, 0.5)**	−0.7 (−1.2, −0.2)	0.1 (−0.5, 0.8)			0.9 (0.0, 1.8)	2.2 (−2.1, 7.5)	−6.2 (−7.0, −5.5)	0.4 (−0.6, 1.5)	−2.1 (−2.6, −1.8)	−4.0 (−4.4, −3.5)	**3.3 (2.6, 4.1)**	**1.3 (0.9, 1.8)**	**10.3 (9.1, 11.6)**	**1.9 (1.6, 2.3)**
Oceania	80.5	1.2	4.5			**3.2**	0.4	0.6	4.8	8.2	4.4	26.4	7.6	**6.1**	13.0
	0.3 (−0.4, 0.9)	−4.3 (−9.3, 1.0)	0.0 (−1.9, 1.5)			**1.6 (0.4, 2.9)**	−1.2 (−10.2, 8.7)	−2.4 (−6.5, 1.6)	1.3 (−0.5, 3.3)	0.2 (−1.7, 1.5)	−0.2 (−3.0, 2.6)	−2.2 (−3.1, −1.2)	−0.6 (−1.5, 0.4)	**14.0 (10.5, 17.7)**	0.6 (−0.6, 1.9)

* Average age-standardized cancer incidence rates (ASRs) between 2000 and 2010 were calculated. ^#^ Average annual percentage changes (AAPCs) with 95% confidence intervals (CIs) in cancer incidence among 20–49-year-old adults during the period of 2000–2010 were calculated using the Joinpoint Regression Program. A maximum of two joinpoints were permitted in this analysis. Bold numbers denote statistically significant positive AAPCs. Abbreviations: AAPC, average annual percentage change; ASR, age-standardized cancer incidence rate; CI, confidence interval; CNS, central nervous system.

## Data Availability

The data that support the findings of this study are openly available in the Cancer Incidence in Five Continents website (https://ci5.iarc.fr/) accessed on 24 October 2022.

## Supplementary Materials

The following supporting information can be downloaded at: https://www.mdpi.com/article/10.3390/curroncol32060324/s1, Supplementary Figure S1. Trends in obesity prevalence among younger populations and early-onset obesity-related cancers by country in females; Supplementary Figure S2. Trends in obesity prevalence among younger populations and early-onset obesity-related cancers by country in males; Supplementary Figure S3. Global trends of early-onset obesity-related and non-obesity-related cancer incidence; Supplementary Figure S4. Global trends of early-onset cancer incidence in females by cancer types; Supplementary Figure S5. Global trends of early-onset cancer incidence by cancer types in males; Supplementary Table S1. Incidence trends of early-onset obesity-related cancers and non-obesity-related cancers by countries in 2000–2012; Supplementary Table S2. Incidence trends of individual early-onset obesity-related cancers among females by countries in 2000–2012; Supplementary Table S3. Incidence trends of individual early-onset obesity-related cancers among males by countries in 2000–2012; Supplementary Table S4. Incidence trends of individual early-onset non-obesity-related cancers among females by countries in 2000–2012; Supplementary Table S5. Incidence trends of individual early-onset non-obesity-related cancers among males by countries in 2000–2012; Supplementary Table S6. Sensitivity analyses of incidence trends of early-onset obesity-related cancers and non-obesity-related cancers by regions in 2000–2010.

## Abbreviations

The following abbreviations are used in this manuscript:

AAPC	average annual percentage changes
ASR	age-standardized cancer incidence rate
BMI	body mass index
CI	confidence interval
