# Supplementary material for: Global Incidence Trend of Early-Onset Obesity-Related and Non-Obesity-Related Cancers
Source: Curr Oncol. 2025 May 31;32(6):324. doi: 10.3390/curroncol32060324 (PMC12191960; doi:10.3390/curroncol32060324)
Supplement: Supplementary file 1 [file curroncol-32-00324-s001.zip › curroncol-3656716-supplementary.pdf]

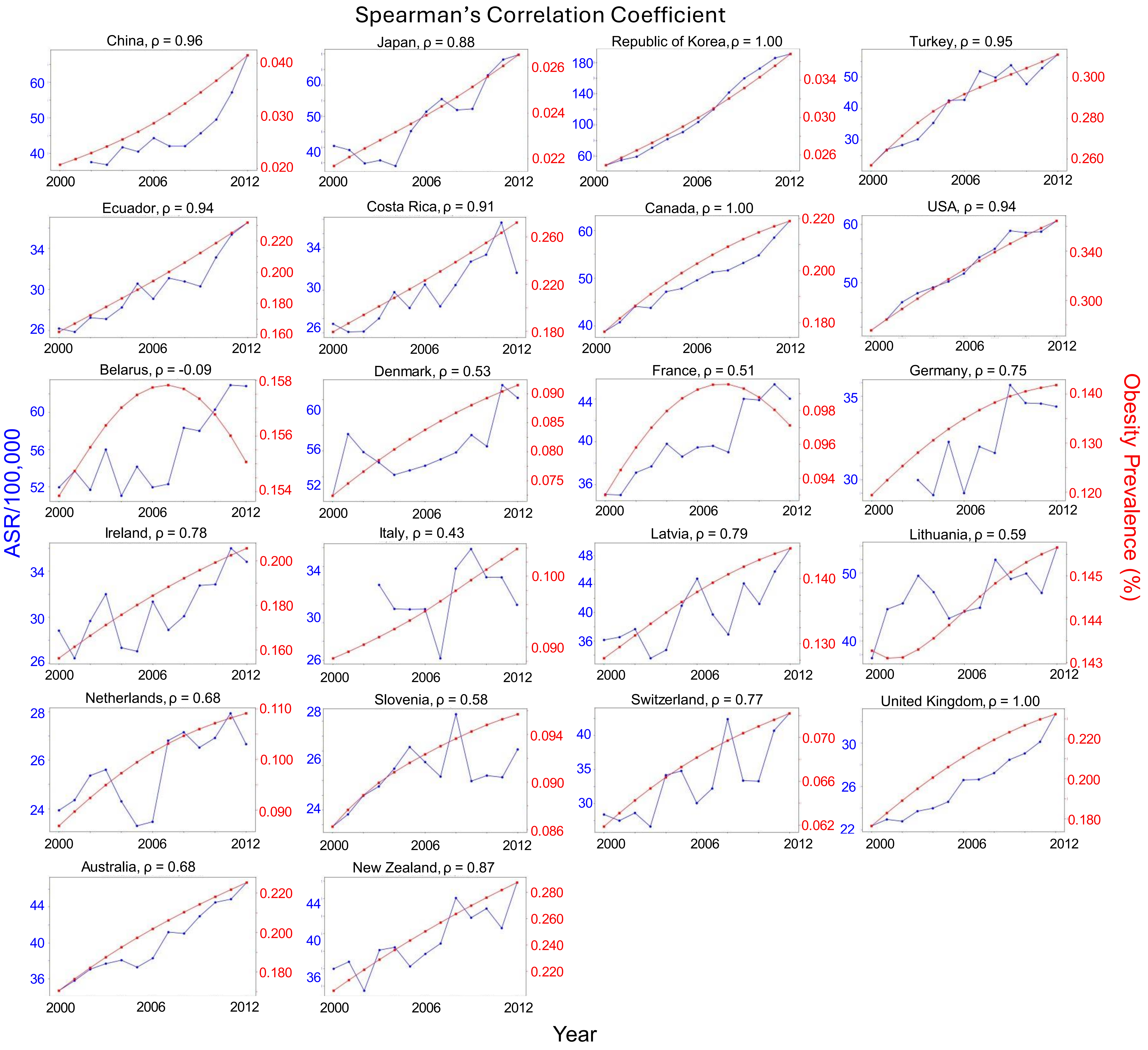

Supplementary Figure S1. Trends in obesity prevalence among younger populations and early-onset obesity-related cancers by country in females.

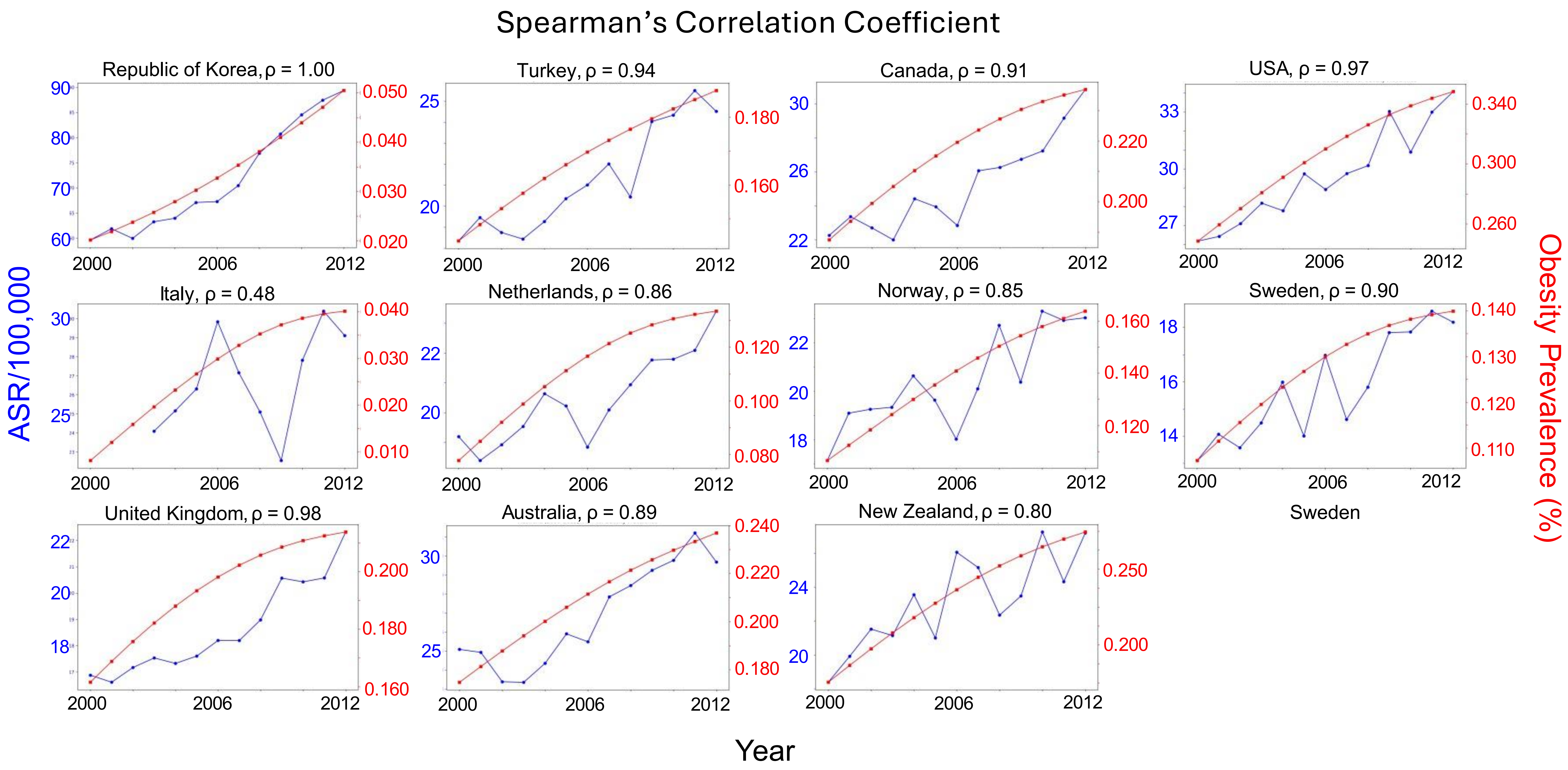

Supplementary Figure S2. Trends in obesity prevalence among younger populations and early-onset obesity-related cancers by country in males.

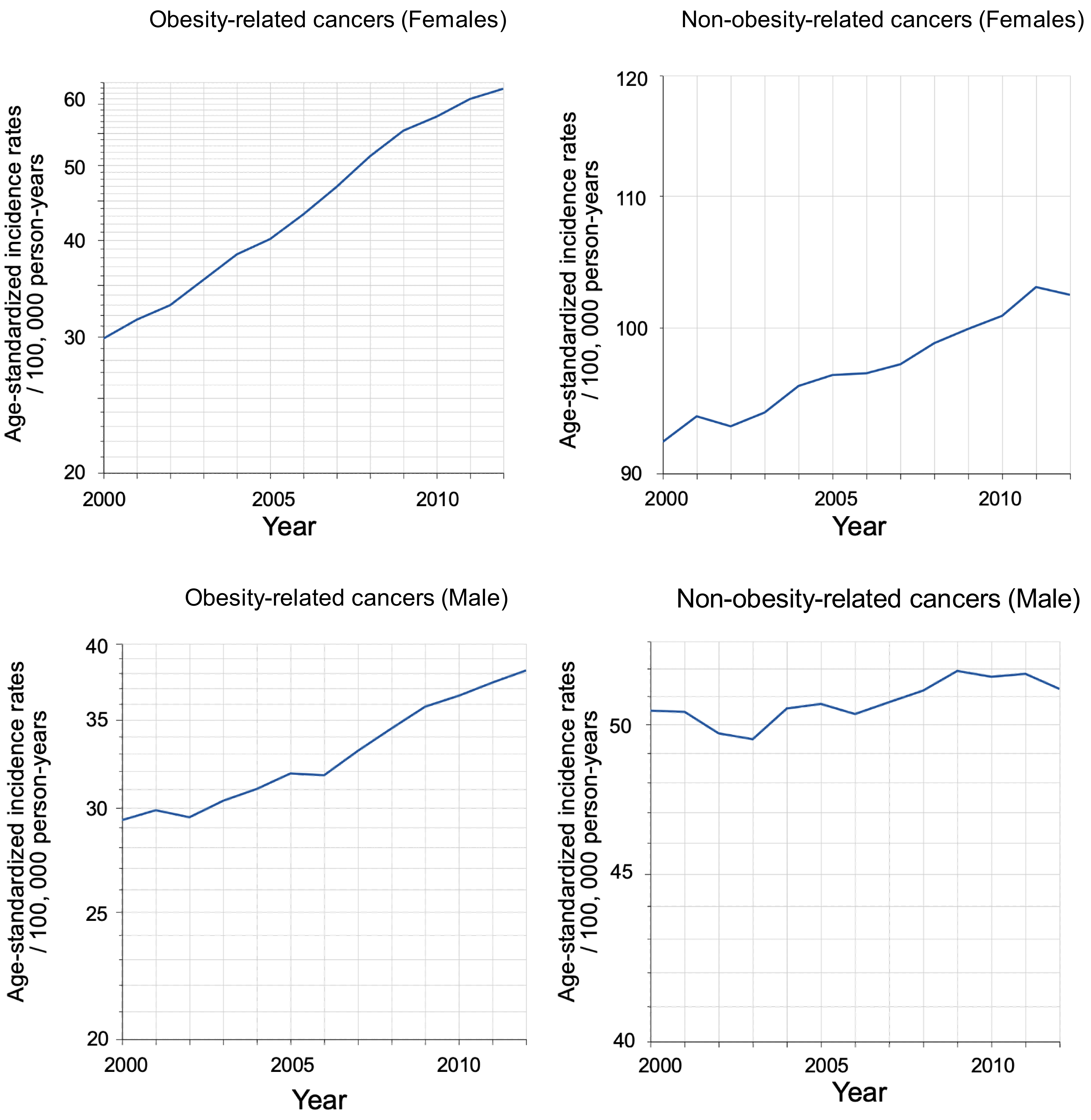

Supplementary Figure S3. Global trends of early-onset obesity-related and non-obesity-related cancer incidence.

Early-onset obesity-related cancer

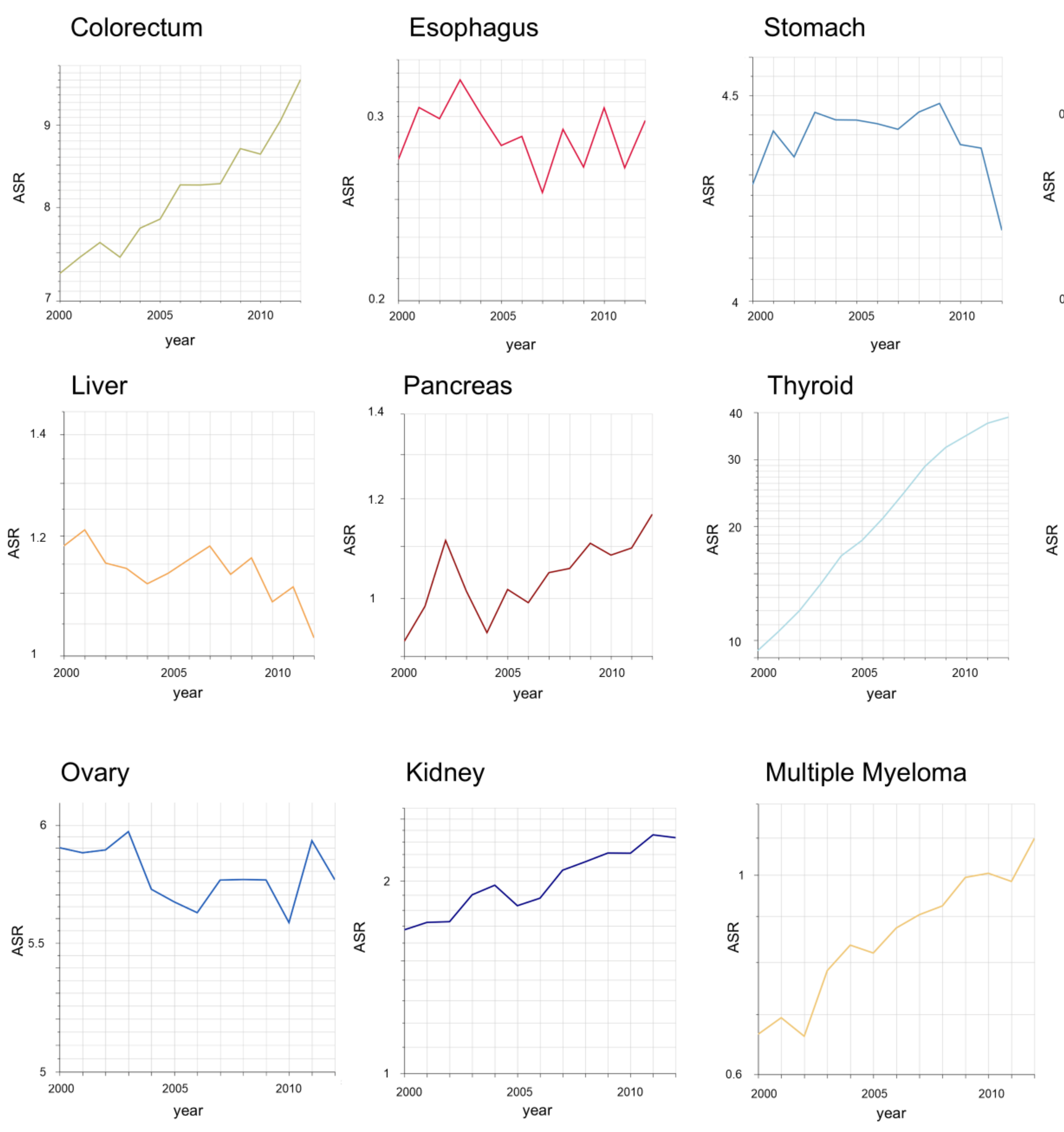

Early-onset non-obesity-related cancer

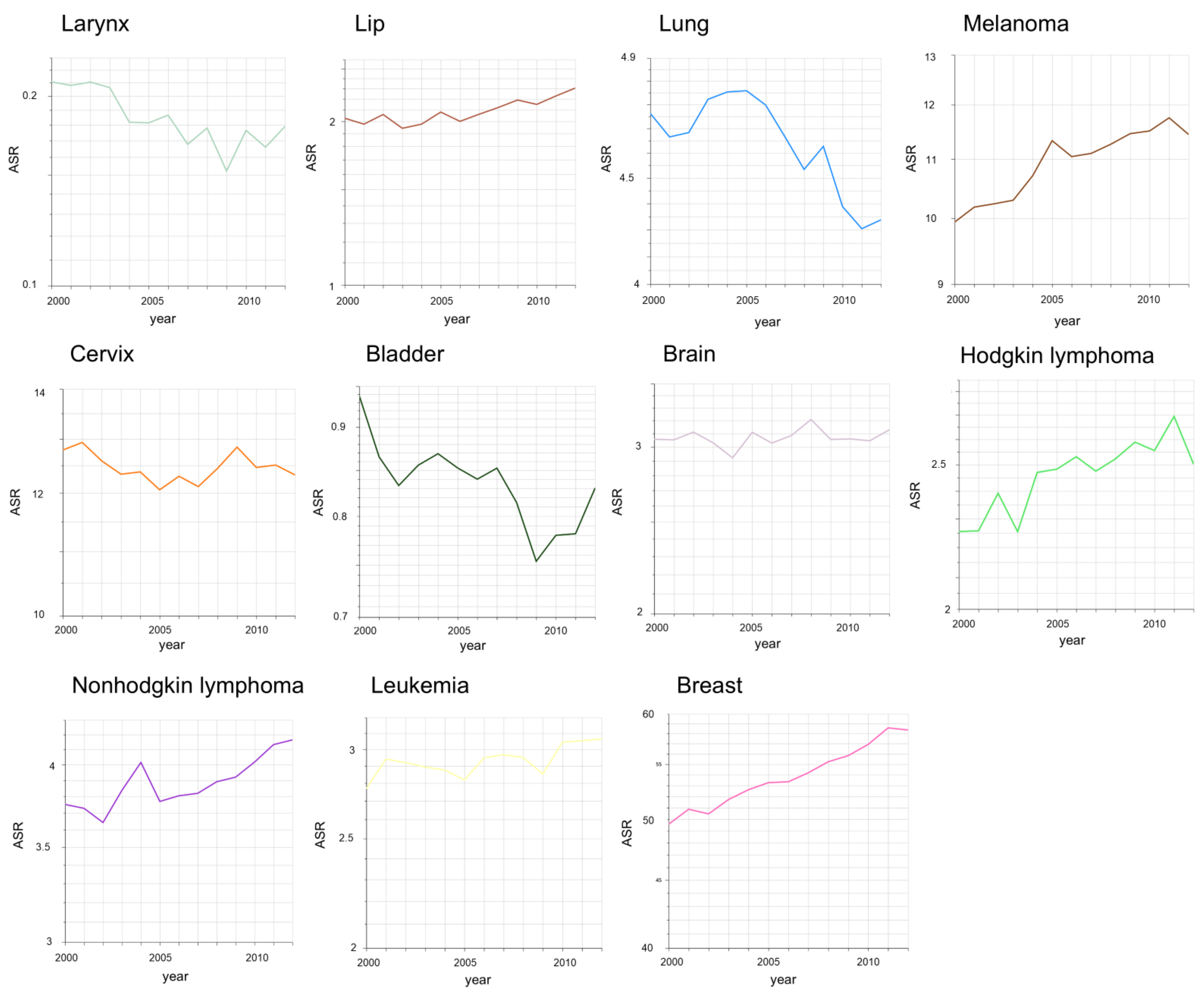

Supplementary Figure S4. Global trends of early-onset cancer incidence in females by cancer types

Early-onset obesity-related cancer

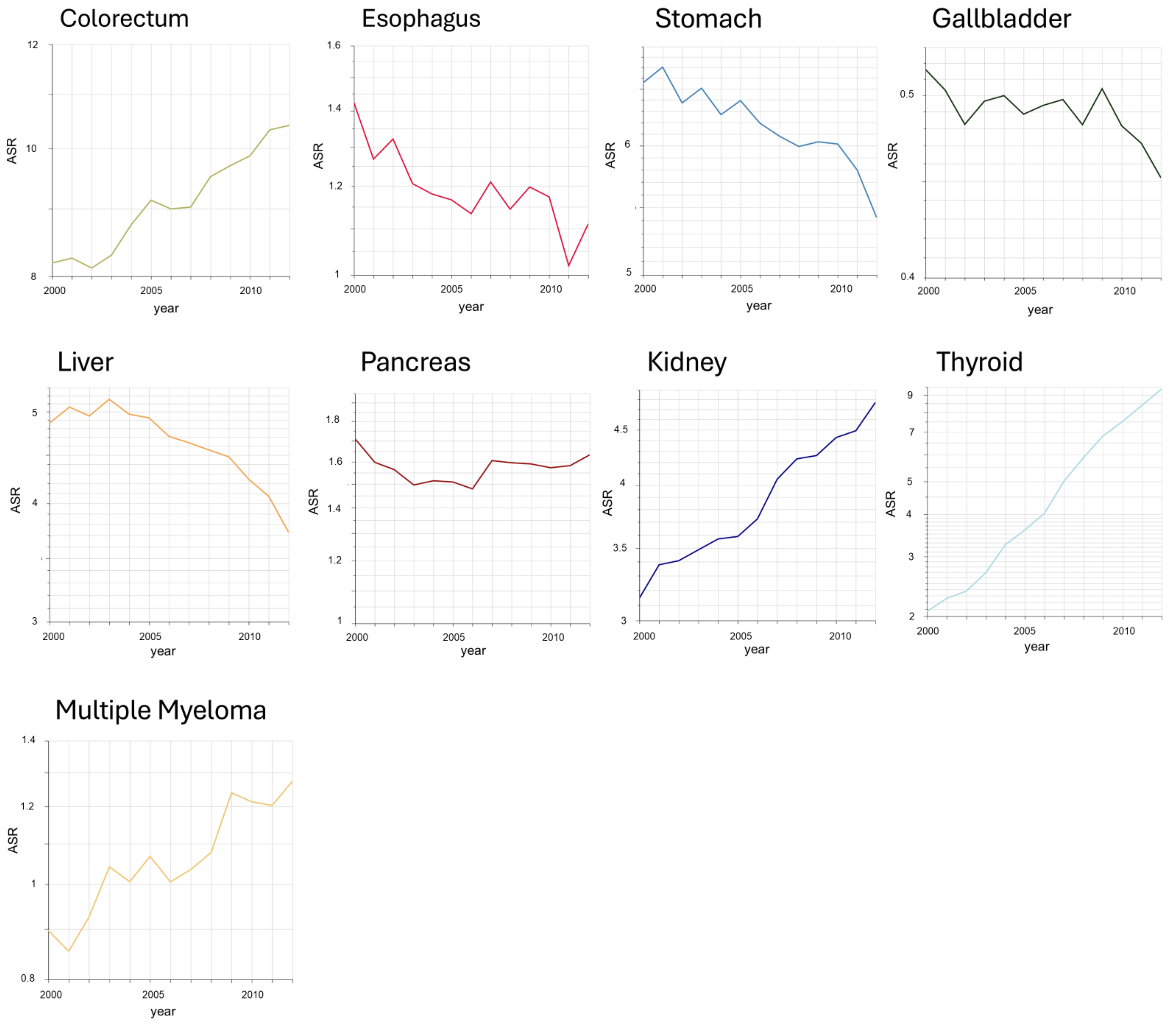

Early-onset non-obesity-related cancer

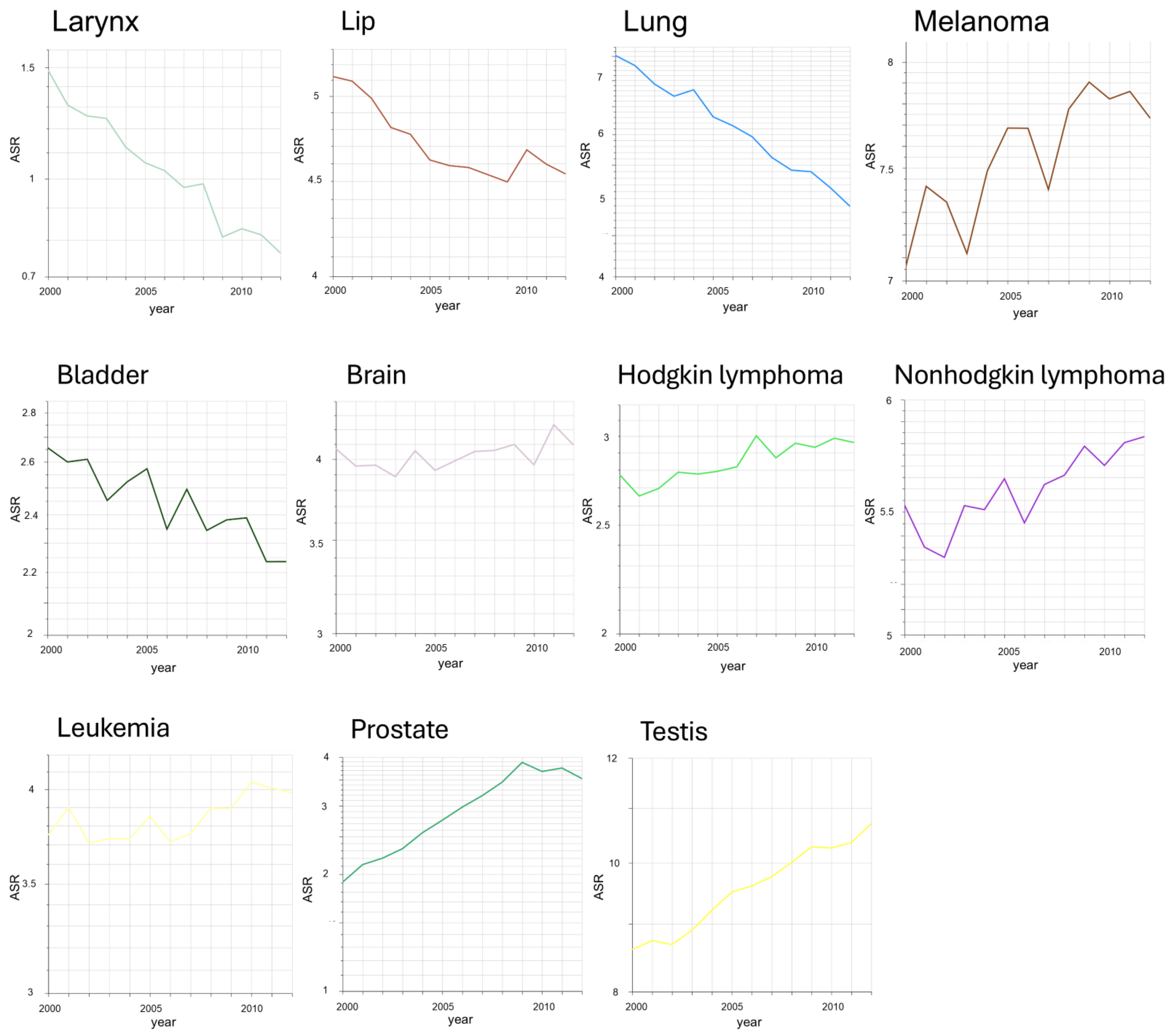

Supplementary Figure S5. Global trends of early-onset cancer incidence by cancer types in males. Data on Kaposi sarcoma was not available.

Supplementary Table S1. Incidence trends of early-onset obesity-related cancers and non-obesity-related cancers by countries in 2000-2012.

Country	Female		Country	Male	
	All obesity-related cancers	All non-obesity-related cancers		All obesity-related cancers	All non-obesity-related cancers
	ASR/100,000* AAPC (95% CI)#	ASR/100,000 AAPC (95% CI)		ASR/100,000* AAPC (95% CI)#	ASR/100,000 AAPC (95% CI)
Africa					
Uganda	22.2	98.0	Uganda	19.9	60.4
	2.9 (-0.7, 6.8)	-1.7 (-3.8, 0.5)		2.8 (-0.3, 6.1)	-2.7 (-6.2, 1.4)
Asia					
China**	**44.7**	**42.6**	China**	36.8	35.6
	**4.1 (3.5, 4.5)**	**1.4 (0.9, 1.8)**		0.6 (0.0, 1.1)	-1.1 (-1.5, -0.7)
India	20.6	29.7	India	16.0	29.1
	0.9 (-1.3, 3.1)	-1.1 (-3.3, 0.9)		0.1 (-2.0, 2.3)	1.1 (0.0, 2.1)
Israel	45.3	41.8	Israel	24.2	57.5
	0.5 (-0.1, 1.1)	-0.7 (-1.4, 0.0)		0.4 (-0.8, 1.7)	-1.4 (-2.3, -0.6)
Japan	**39.5**	**26.1**	Japan	35.0	**26.4**
	**2.0 (1.0, 3.5)**	**3.3 (2.3, 4.4)**		-2.0 (-3.3, -0.8)	**1.4 (0.4, 2.7)**
Kuwait	31.4	16.2	Kuwait	15.8	30.8
	0.9 (-1.1, 2.8)	0.7 (-2.0, 3.5)		2.2 (-1.8, 6.6)	0.5 (-2.2, 3.4)
Republic of Korea**	**116.7**	**28.3**	Republic of Korea**	**70.9**	**22.3**
	**13.2 (12.3, 14.0)**	**3.6 (3.3, 3.8)**		**4.2 (3.7, 4.9)**	**1.3 (0.6, 1.9)**
Thailand	26.4	35.3	Thailand	27.2	23.5
	0.2 (-1.1, 1.4)	-1.5 (-2.7, -0.3)		0.6 (-0.9, 2.2)	1.1 (-0.6, 2.3)
Turkey**	**41.3**	**23.9**	Turkey**	**21.0**	52.3
	**7.6 (5.7, 9.3)**	**2.5 (1.8, 3.3)**		**2.6 (1.8, 3.6)**	-0.7 (-1.6, 0.1)
South America					
Brazil**	39.7	43.9	Brazil	24.9	42.4
	1.5 (-0.4, 3.5)	-2.7 (-4.6, -0.8)		-0.3 (-2.7, 2.2)	0.1 (-3.7, 2.9)
Chile	39.8	38.1	Chile	24.8	42.7
	0.1 (-2.9, 3.3)	-2.7 (-5.6, 0.2)		-4.5 (-8.5, -0.3)	-0.6 (-4.6, 3.6)
Colombia	36.7	39.8	Colombia	21.3	34.9
	1.4 (-1.2, 4.2)	-2.0 (-3.1, -0.9)		0.5 (-1.6, 2.8)	-1.5 (-3.4, 0.5)
Ecuador**	**50.2**	36.7	Ecuador	20.4	**33.0**
	**8.5 (6.7, 10.3)**	1.1 (-0.6, 2.7)		3.5 (0.0, 7.2)	**3.3 (1.5, 4.8)**
Central America					
Costa Rica**	**41.7**	32.8	Costa Rica	18.0	**25.8**
	**3.7 (2.4, 5.0)**	-0.3 (-2.5, 2.0)		-0.5 (-1.7, 0.8)	**1.7 (0.3, 3.1)**
North America					
Canada**	**49.0**	44.8	Canada**	**25.1**	55.6
	**3.5 (3.0, 4.1)**	0.1 (-0.2, 0.5)		**2.5 (1.9, 3.1)**	0.0 (-0.3, 0.2)
United States of America**	**50.5**	46.5	United States of America**	**28.5**	65.9
	**2.9 (2.5, 3.3)**	-0.3 (-0.5, 0.0)		**2.1 (1.7, 2.5)**	-1.1 (-1.3, -0.9)
Europe					
Belarus	**55.8**	**37.9**	Belarus	33.3	47.4
	**1.6 (0.9, 2.3)**	**1.4 (0.7, 2.1)**		0.4 (-0.3, 1.2)	0.5 (-0.4, 1.4)
Bulgaria	**37.8**	**48.8**	Bulgaria	23.8	53.8
	**1.3 (0.3, 2.4)**	**1.0 (0.7, 1.2)**		-0.1 (-2.1, 2.0)	0.0 (-0.9, 0.8)
Croatia	47.2	48.4	Croatia	29.2	71.5
	-0.5 (-1.6, 1.5)	-1.7 (-2.3, -1.1)		-2.6 (-3.7, -0.6)	-3.6 (-4.5, -1.8)
Czech Republic	40.1	**48.0**	Czech Republic	26.9	56.4
	0.2 (-0.5, 1.0)	**0.5 (0.1, 1.0)**		-1.1 (-2.4, 0.0)	-1.0 (-1.5, -0.6)
Denmark	**27.9**	**78.8**	Denmark	19.4	**76.5**
	**1.6 (0.6, 2.6)**	**1.9 (1.3, 2.5)**		1.2 (0.0, 2.5)	**2.1 (1.5, 2.6)**
Estonia	35.0	**49.8**	Estonia	25.1	45.3
	0.0 (-1.2, 1.3)	**1.9 (1.1, 2.7)**		-0.2 (-2.3, 2.0)	1.2 (-0.9, 3.5)
France	**37.4**	**49.3**	France**	26.5	74.3
	**1.8 (1.1, 2.4)**	**0.8 (0.3, 1.4)**		0.6 (-0.2, 1.4)	-0.8 (-1.3, -0.3)
Germany	**30.3**	50.9	Germany	23.1	69.6
	**2.1 (0.3, 4.0)**	0.2 (-0.8, 1.2)		1.1 (-0.3, 2.6)	-0.9 (-1.8, 0.0)
Iceland	38.3	71.9	Iceland	19.0	57.9
	0.8 (-3.9, 5.7)	-0.3 (-2.7, 2.1)		1.9 (-4.9, 9.2)	-1.6 (-4.8, 1.8)
Ireland	**29.5**	**48.3**	Ireland	21.3	**56.8**
	**2.0 (0.6, 3.6)**	**1.3 (0.7, 2.1)**		1.7 (-0.1, 3.7)	**2.1 (1.5, 2.6)**
Italy	**63.4**	**48.0**	Italy	**32.8**	65.9
	**1.4 (0.3, 3.0)**	**0.8 (0.2, 1.3)**		**1.2 (0.5, 2.0)**	0.8 (-0.5, 2.1)
Latvia	**40.1**	**39.5**	Latvia	24.5	**42.5**
	**2.4 (1.2, 3.6)**	**3.1 (1.8, 4.0)**		0.7 (-0.5, 1.6)	**3.1 (1.8, 4.5)**
Lithuania	**46.8**	**50.9**	Lithuania	28.4	45.8
	**1.7 (0.1, 3.3)**	**0.8 (0.2, 1.3)**		0.9 (-0.5, 1.6)	-0.3 (-1.8, 1.4)
Malta	40.0	**31.5**	Malta	18.7	44.9
	1.7 (-2.2, 5.7)	**4.3 (2.4, 6.4)**		6.0 (-0.8, 13.3)	1.7 (-1.3, 4.8)
Netherlands	**25.6**	**56.9**	Netherlands	**20.4**	**61.4**
	**1.1 (0.2, 2.0)**	**1.2 (0.7, 1.6)**		**1.7 (1.1, 2.3)**	**1.7 (1.4, 2.1)**
Norway	33.6	**64.1**	Norway	**19.9**	**71.2**
	0.6 (-0.5, 1.8)	**1.3 (0.5, 2.0)**		**2.0 (0.9, 3.1)**	**1.1 (0.4, 1.9)**
Poland	38.8	35.7	Poland	20.1	46.4
	1.2 (-1.1, 3.2)	-1.9 (-3.6, -0.1)		0.5 (-0.8, 1.9)	-2.3 (-3.8, -0.5)
Slovakia	**39.1**	**47.1**	Slovakia**	29.0	64.5
	**2.3 (0.4, 3.4)**	**1.1 (0.6, 1.6)**		1.1 (-0.2, 2.4)	-1.0 (-1.7, -0.4)
Slovenia	**36.2**	53.5	Slovenia	25.6	66.6
	**2.0 (0.4, 3.4)**	-0.3 (-1.2, 0.7)		0.2 (-2.1, 2.5)	-0.9 (-2.3, 0.6)
Spain	35.4	40.8	Spain	25.5	65.2
	1.0 (-0.5, 2.4)	-0.4 (-1.3, 0.4)		-0.2 (-1.6, 1.2)	-1.3 (-3.2, 0.6)
Sweden	25.4	**57.3**	Sweden	**15.3**	**55.8**
	0.9 (-0.3, 2.1)	**1.3 (0.7, 2.0)**		**2.6 (1.6, 3.6)**	**1.5 (1.0, 2.0)**
Switzerland	**32.2**	50.0	Switzerland	22.8	71.7
	**3.2 (0.8, 5.7)**	0.0 (-1.1, 1.0)		-0.4 (-2.0, 1.3)	-0.2 (-1.0, 0.6)
Ukraine	**44.3**	**44.2**	Ukraine	27.8	45.4
	**1.3 (0.8, 1.7)**	**0.8 (0.5, 1.2)**		-0.7 (-1.1, -0.3)	-0.3 (-0.7, 0.1)
United Kingdom – England and Wales**	**26.2**	**44.9**	United Kingdom – England and Wales**	**18.6**	**52.5**
	**3.3 (2.6, 3.7)**	**1.0 (0.6, 1.5)**		**2.4 (1.9, 2.8)**	**0.9 (0.5, 1.5)**
United Kingdom – Northern Ireland	29.7	50.8	United Kingdom – Northern Ireland	19.9	54.8
	1.7 (-0.4, 3.9)	1.5 (0.0, 3.0)		2.2 (-0.7, 5.3)	0.8 (-0.3, 1.9)
United Kingdom - Scotland**	**27.9**	**53.5**	United Kingdom - Scotland	19.9	60.6
	**2.5 (1.8, 3.3)**	**1.1 (0.6, 1.6)**		0.8 (-0.1, 1.8)	0.3 (-0.7, 1.2)
Oceania					
Australia**	**40.6**	60.7	Australia	**27.2**	82.5
	**2.4 (2.0, 2.7)**	0.0 (-0.3, 0.3)		**1.6 (0.7, 2.4)**	0.3 (0.0, 0.7)
New Zealand**	**35.3**	60.7	New Zealand**	**23.2**	70.0
	**2.2 (1.3, 3.2)**	-0.2 (-1.1, 0.6)		**2.6 (0.6, 4.6)**	-1.4 (-2.2, -0.4)

* Average age-standardized cancer incidence rates (ASRs) between 2000 and 2010 were calculated.
# Average annual percentage changes (AAPCs) with 95% confidence intervals (CIs) in cancer incidence among 20-49-year-old adults during the period of 2000-2012 were calculated using the Joinpoint Regression Program (version 4.9.0.1). A maximum of two joinpoints were permitted in this analysis.
** These regions denote statistically significant differences in AAPCs between obesity-related and non-obesity-related cancers.
Bold numbers denote statistically significant positive AAPCs.
Abbreviations: AAPC, Average annual percentage change; ASR, age-standardized cancer incidence rate; CI, confidence interval.

Supplementary Table S2. Incidence trends of individual early-onset obesity-related cancers among females by countries in 2000-2012.

Country	All obesity-related cancers	All obesity-related cancers excluding thyroid cancer	Colorectum	Corpus Uteri	Esophagus	Gallbladder and extrahepatic bile duct	Kidney	Liver	Multiple Myeloma	Ovary	Pancreas	Stomach	Thyroid
	ASR/100,000[*]<br>AAPC (95% CI)[#]	ASR/100,000<br>AAPC (95% CI)	ASR/100,000<br>AAPC (95% CI)	ASR/100,000<br>AAPC (95% CI)	ASR/100,000<br>AAPC (95% CI)	ASR/100,000<br>AAPC (95% CI)	ASR/100,000<br>AAPC (95% CI)	ASR/100,000<br>AAPC (95% CI)	ASR/100,000<br>AAPC (95% CI)	ASR/100,000<br>AAPC (95% CI)	ASR/100,000<br>AAPC (95% CI)	ASR/100,000<br>AAPC (95% CI)	ASR/100,000<br>AAPC (95% CI)
Africa													
Uganda	22.2	20.6	3.5	2.1	**5.7**	0.4	0.5	**4.4**	0.9	4.8	1.3	2.3	**1.6**
	2.9 (-0.7, 6.8)	2.5 (-1.2, 6.4)	5.5 (-3.3, 15.6)	-2.6 (-15.2, 12.2)	**16.7 (3.2, 27.8)**	4.3 (-1.7, 10.6)	1.5 (-16.5, 24.5)	**9.4 (1.5, 18.3)**	6.7 (-6.8, 22.2)	-0.9 (-8.0, 6.8)	-1.0 (-17.8, 20.5)	-12.3 (-22.4, -0.8)	**13.7 (5.4, 23.2)**
Asia													
China	**44.7**	29.9	7.6	**6.2**	1.4	0.5	1.4	2.0	0.3	**6.3**	0.7	4.6	**14.8**
	**4.1 (3.5, 4.5)**	0.5 (-0.3, 1.2)	-0.8 (-2.9, 1.5)	**3.2 (1.3, 5.1)**	-5.1 (-12.9, 0.9)	-3.7 (-8.7, 1.2)	3.2 (-0.1, 6.6)	-3.2 (-5.0, -1.4)	1.3 (-8.0, 11.8)	**4.8 (1.9, 8.6)**	-1.0 (-7.1, 4.6)	-3.2 (-5.1, -1.2)	**10.9 (9.7, 12.9)**
India	20.6	17.5	3.2	1.2	2.3	0.8	0.3	0.6	0.4	5.1	0.6	3.5	3.0
	0.9 (-1.3, 3.1)	1.0 (-1.0, 3.0)	1.1 (-3.2, 5.6)	2.4 (-3.1, 8.4)	-4.5 (-8.1, -0.7)	4.9 (-3.7, 15.5)	3.9 (-12.0, 22.6)	1.1 (-11.3, 15.6)	0 (-10.1, 11.2)	1.9 (-1.3, 5.5)	0.2 (-7.5, 8.8)	0.7 (-3.4, 4.9)	1.9 (-7.0, 9.4)
Israel	45.3	26.2	9.0	4.8	0.4	0.3	2.2	0.3	0.8	5.2	1.2	2.3	**19.1**
	0.5 (-0.1, 1.1)	-1.6 (-2.9, -0.2)	-1.1 (-3.2, 1.2)	0.9 (-4.0, 4.5)	-2.0 (-17.3, 17.5)	3.3 (-2.2, 11.0)	0.8 (-2.9, 4.6)	-0.6 (-7.5, 6.9)	-5.7 (-14.2, 3.7)	-3.2 (-5.5, -0.9)	-3.0 (-8.0, 2.3)	-1.4 (-5.8, 3.2)	**3.4 (2.2, 4.5)**
Japan	**39.5**	**32.9**	**8.7**	**4.7**	1.6	0.6	**0.9**	1.0	0.3	**6.7**	1.0	8.4	**6.7**
	**2.0 (1.0, 3.5)**	**1.8 (0.3, 3.4)**	**1.7 (0.1, 3.4)**	**9.3 (5.9, 13.0)**	6.7 (-3.4, 17.6)	-5.1 (-7.5, -2.6)	**6.2 (2.2, 10.4)**	-2.3 (-8.2, 4.2)	2.5 (-3.0, 8.5)	**4.1 (2.4, 5.7)**	2.6 (-4.3, 10.0)	-3.4 (-6.2, -0.5)	**5.2 (3.0, 8.8)**
Kuwait	31.4	17.6	8.2	2.3	0.6	0.8	1.0	0.9	1.0	2.6	1.1	1.4	**13.7**
	0.9 (-1.1, 2.8)	-2.9 (-5.2, 0.1)	-3.1 (-9.4, 6.1)	-9.9 (-19.5, 0.5)	NA	-7.4 (-16.1, 2.1)	0.7 (-14.4, 12.6)	-6.4 (-13.9, 1.9)	-7.0 (-14.7, 3.0)	-5.7 (-17.2, 7.3)	0.7 (-6.9, 9.3)	4.2 (-4.4, 13.9)	**10.0 (8.7, 11.6)**
Republic of Korea	**116.7**	**40.5**	**9.6**	**3.9**	0.6	1.0	**1.5**	3.1	**0.3**	**5.5**	0.9	14.5	**76.3**
	**13.2 (12.3, 14.0)**	**2.6 (1.9, 3.2)**	**4.7 (3.9, 5.4)**	**6.4 (4.7, 7.9)**	5.0 (-6.0, 17.8)	-3.1 (-5.6, -0.6)	**6.4 (2.9, 10.0)**	-2.5 (-3.7, -1.3)	**5.2 (0.1, 10.5)**	**2.7 (1.6, 3.9)**	3.6 (-0.2, 5.7)	0.4 (-0.1, 1.1)	**23.4 (22.1, 24.6)**
Thailand	26.4	20.0	4.9	2.1	0.8	0.5	0.3	4.2	0.2	5.5	0.5	1.8	6.3
	0.2 (-1.1, 1.4)	-0.3 (-1.1, 0.6)	0.4 (-2.3, 3.1)	2.0 (-2.2, 6.4)	-2.4 (-14.1, 10.8)	3.2 (-4.6, 11.6)	2.8 (-8.5, 15.6)	-3.6 (-6.5, -0.7)	0.3 (-9.2, 10.9)	-0.6 (-3.7, 2.5)	6.3 (-3.5, 17.5)	1.5 (-2.7, 5.8)	0.0 (-3.0, 2.6)
Turkey	**41.3**	**22.5**	5.1	**4.4**	0.7	0.4	**1.4**	0.5	0.6	**5.1**	0.9	3.3	**18.8**
	**7.6 (5.7, 9.3)**	**3.8 (2.7, 4.7)**	4.0 (-0.2, 7.2)	**5.2 (1.1, 8.2)**	6.9 (-0.3, 13.6)	-0.8 (-6.6, 5.2)	**5.1 (1.6, 8.7)**	-3.4 (-9.0, 2.5)	6.7 (-2.3, 16.6)	**2.2 (0.3, 4.3)**	2.1 (-2.6, 7.1)	3.3 (-0.2, 6.2)	**14.0 (11.6, 16.4)**
South America													
Brazil	39.7	24.4	9.4	2.3	2.6	0.9	1.4	0.8	0.7	4.6	1.1	3.5	15.3
	1.5 (-0.4, 3.5)	-2.0 (-3.8, 0.4)	-2.3 (-7.2, 4.6)	-4.7 (-12.2, 3.7)	4.9 (-16.3, 27.2)	-6.0 (-11.3, -0.5)	9.9 (-3.8, 28.7)	-3.5 (-12.2, 6.5)	-4.3 (-11.5, 1.2)	0.0 (-4.8, 5.1)	2.1 (-13.8, 20.5)	-1.8 (-6.6, 3.3)	4.1 (-0.6, 9.0)
Chile	39.8	29.4	4.9	3.5	1.9	6.0	3.2	1.8	2.0	5.5	1.9	4.9	10.4
	0.1 (-2.9, 3.3)	-2.3 (-7.1, 2.8)	2.5 (-2.7, 7.7)	3.2 (-8.4, 16.4)	0.6 (-2.7, 3.7)	-3.0 (-9.4, 3.8)	2.8 (-8.8, 15.7)	-9.3 (-30.6, 17.6)	-0.9 (-21.4, 20.5)	-0.2 (-12.1, 13.3)	-1.6 (-8.6, 5.9)	-6.7 (-14.2, 1.5)	7.4 (-0.1, 15.4)
Colombia	36.7	23.9	6.5	2.0	0.7	0.9	1.2	0.5	0.4	5.7	0.9	5.6	12.8
	1.4 (-1.2, 4.2)	0.6 (-2.1, 3.5)	2.0 (-0.3, 4.2)	-1.6 (-5.8, 2.8)	-6.0 (-11.0, 2.1)	-3.3 (-12.6, 7.1)	-6.8 (-15.5, 0.2)	2.7 (-7.2, 14.0)	1.7 (-13.6, 20.0)	-1.6 (-4.7, 1.8)	-6.0 (-18.5, 8.6)	1.0 (-2.5, 4.7)	3.2 (-0.7, 7.2)
Ecuador	**50.2**	**23.8**	**4.5**	2.1	0.5	1.8	1.2	1.2	0.4	5.0	0.7	6.8	**26.3**
	**8.5 (6.7, 10.3)**	**2.8 (1.1, 4.6)**	**4.8 (0.5, 9.4)**	5.1 (-3.3, 14.3)	1.3 (-12.1, 17.3)	-4.6 (-12.2, 3.8)	4.4 (-4.3, 14.3)	1.8 (-8.3, 12.9)	-1.0 (-5.9, 4.2)	4.4 (-3.3, 11.5)	0.0 (-13.9, 12.1)	3.9 (-0.1, 8.0)	**14.6 (12.4, 16.8)**
Central America													
Costa Rica	**41.7**	22.5	5.3	3.1	0.3	0.7	1.1	0.8	0.4	4.2	0.7	6.1	**19.2**
	**3.7 (2.4, 5.0)**	0.7 (-1.2, 2.1)	1.4 (-2.4, 5.5)	2.6 (-2.1, 7.7)	3.3 (-13.4, 22.8)	-6.1 (-13.3, 1.9)	-2.6 (-12.9, 9.1)	1.7 (-5.0, 9.1)	0.9 (-4.0, 7.6)	0.3 (-2.5, 3.2)	-4.2 (-11.5, 3.9)	0.8 (-0.6, 2.1)	**8.4 (7.0, 9.7)**
North America													
Canada	**49.0**	**27.0**	**8.7**	**5.8**	1.0	0.4	2.6	**0.4**	0.6	5.8	**1.1**	1.4	**22.0**
	**3.5 (3.0, 4.1)**	**2.1 (1.5, 2.7)**	**1.6 (0.9, 2.7)**	**3.4 (1.9, 5.0)**	1.6 (-6.8, 10.7)	0 (-3.0, 3.1)	1.7 (-0.3, 3.7)	**5.0 (1.5, 8.6)**	1.5 (-0.7, 3.9)	1.4 (-0.1, 2.7)	**2.2 (0.4, 4.0)**	1.9 (-0.2, 4.1)	**6.2 (5.4, 7.0)**
United States of America	**50.5**	**30.7**	**8.9**	**8.0**	0.9	0.4	**2.9**	0.7	0.8	5.9	**1.2**	**1.6**	**19.9**
	**2.9 (2.5, 3.3)**	**1.7 (1.5, 1.8)**	**2.0 (1.5, 2.5)**	**1.6 (1.3, 2.1)**	-4.6 (-8.0, -0.9)	1.1 (-2.5, 4.8)	**3.1 (0.7, 5.8)**	-0.6 (-3.0, 2.4)	2.1 (-1.4, 5.7)	0.3 (-0.6, 1.1)	**2.5 (1.1, 3.9)**	**2.8 (0.4, 4.8)**	**4.8 (4.3, 5.3)**
Europe													
Belarus	**55.8**	**37.1**	6.5	**8.3**	1.6	**0.2**	**3.9**	0.5	0.5	**10.2**	0.9	5.9	**18.7**
	**1.6 (0.9, 2.3)**	**1.6 (0.9, 2.3)**	-0.2 (-1.6, 1.3)	**3.0 (1.5, 4.6)**	4.7 (-5.2, 15.8)	**9.7 (1.0, 19.4)**	**2.0 (0.3, 3.7)**	0.9 (-4.4, 6.6)	2.9 (-0.9, 6.9)	**3.8 (2.1, 5.6)**	0.2 (-4.0, 4.6)	-2.9 (-3.8, -1.9)	**1.6 (0.7, 2.5)**
Bulgaria	**37.8**	**32.2**	7.4	**7.0**	0.8	0.3	1.7	0.7	0.4	10.5	1.3	2.7	**5.6**
	**1.3 (0.3, 2.4)**	**1.3 (0.5, 1.9)**	0.1 (-1.7, 1.9)	**2.1 (0.1, 4.2)**	3.6 (-9.3, 18.4)	-1.5 (-8.9, 6.5)	3.0 (-1.5, 7.7)	4.4 (-4.6, 14.4)	3.1 (-9.3, 17.7)	0.0 (-2.0, 2.1)	1.1 (-4.9, 7.5)	-0.9 (-2.4, 0.7)	**4.9 (1.4, 8.6)**
Croatia	47.2	31.6	8.4	4.5	1.4	0.6	2.7	0.8	0.7	9.9	1.1	2.6	**15.6**
	-0.5 (-1.6, 1.5)	-2.4 (-3.9, 0.0)	-0.9 (-3.3, 1.5)	3.0 (-0.5, 6.8)	-8.6 (-16.4, -0.5)	-7.6 (-18.8, 5.4)	0.2 (-3.7, 4.3)	-5.5 (-12.4, 2.1)	-2.1 (-6.0, 2.0)	-3.4 (-4.7, -1.3)	0.3 (-6.8, 8.0)	-7.1 (-11.6, 0.0)	**3.3 (2.1, 4.3)**
Czech Republic	40.1	30.3	7.8	5.8	1.6	0.6	2.8	0.5	0.5	8.6	1.4	2.2	**9.7**
	0.2 (-0.5, 1.0)	-1.8 (-2.6, -0.9)	0.0 (-0.7, 0.7)	0.4 (-0.7, 1.6)	-0.5 (-10.5, 10.6)	-10.8 (-16.9, -3.7)	-1.6 (-4.1, 1.0)	-1.8 (-8.4, 5.5)	0.5 (-4.1, 5.4)	-4.1 (-5.0, -2.6)	2.1 (-1.5, 5.8)	-0.1 (-4.1, 2.4)	**7.3 (5.0, 9.5)**
Denmark	**27.9**	22.4	**7.8**	2.8	1.2	0.3	**1.4**	0.5	0.7	6.0	1.3	1.3	**5.5**
	**1.6 (0.6, 2.6)**	0.7 (-0.5, 1.9)	**2.2 (0.4, 4.0)**	1.9 (-1.7, 5.6)	3.5 (-7.2, 15.6)	4.7 (-5.0, 15.4)	**7.1 (1.6, 13.2)**	-0.5 (-8.4, 8.5)	4.8 (-1.1, 11.1)	-3.4 (-6.6, -0.1)	0.3 (-6.0, 7.1)	0.1 (-6.2, 6.8)	**5.3 (3.4, 7.2)**
Estonia	35.0	29.4	6.1	**6.0**	1.1	0.5	1.9	0.7	0.8	8.0	1.1	4.9	5.6
	0.0 (-1.2, 1.3)	0.0 (-1.7, 1.7)	0.2 (-5.6, 6.3)	**3.8 (0.1, 7.8)**	1.5 (0.0, 2.9)	4.8 (-10.9, 26.9)	0.7 (-7.8, 10.1)	-3.2 (-17.2, 13.1)	-4.6 (-20.7, 15.2)	-0.4 (-5.7, 5.2)	-3.5 (-12.7, 6.6)	-4.3 (-9.5, 1.4)	0.8 (-4.7, 6.5)
France	**37.4**	22.2	**7.5**	2.4	1.9	0.2	**2.5**	0.7	0.7	5.0	1.3	1.4	**15.3**
	**1.8 (1.1, 2.4)**	0.0 (-1.1, 1.2)	**2.8 (1.0, 3.8)**	-2.0 (-7.4, 2.0)	3.3 (-7.7, 15.9)	1.7 (-6.2, 10.3)	**5.1 (0.1, 10.4)**	-4.9 (-10.2, 2.7)	1.7 (-4.1, 7.9)	-4.5 (-6.6, -2.1)	1.3 (-3.4, 6.4)	-3.5 (-7.6, 0.8)	**4.5 (3.5, 5.4)**
Germany	**30.3**	24.2	7.7	**3.3**	1.4	0.3	1.8	0.8	0.8	6.0	1.1	2.0	**6.1**
	**2.1 (0.3, 4.0)**	1.2 (-0.1, 2.4)	1.6 (-0.9, 4.1)	**5.0 (1.4, 8.7)**	0.0 (-10.0, 11.1)	5.6 (-1.6, 13.4)	3.4 (-1.3, 8.4)	3.5 (-6.1, 14.0)	-0.3 (-11.3, 12.0)	-2.1 (-4.3, 0.9)	2.5 (-4.9, 9.7)	-5.7 (-11.3, 2.1)	**5.8 (0.7, 11.3)**
Iceland	38.3	23.3	7.4	5.3	1.7	1.4	3.6	1.4	2.0	4.5	2.0	2.4	15.1
	0.8 (-3.9, 5.7)	0.2 (-4.8, 5.5)	1.3 (-8.6, 12.7)	3.4 (-7.3, 15.4)	NA	NA	0.4 (-9.7, 8.2)	-1.8 (-7.5, 4.4)	-3.2 (-14.2, 9.8)	0.7 (-7.5, 9.8)	-0.3 (-7.8, 7.5)	-1.2 (-9.5, 7.8)	1.7 (-6.5, 10.6)
Ireland	**29.5**	23.6	7.9	3.5	1.7	0.2	**2.1**	0.4	0.7	5.9	0.9	1.5	**5.8**
	**2.0 (0.6, 3.6)**	0.2 (-1.0, 1.3)	0.6 (-1.6, 2.8)	-0.1 (-2.4, 2.4)	-4.3 (-10.2, 2.1)	-3.7 (-17.3, 12.9)	**4.7 (0.8, 8.7)**	10.9 (-4.6, 21.1)	0.6 (-11.1, 13.7)	-1.4 (-3.5, 0.8)	-1.1 (-8.2, 6.5)	-0.4 (-4.4, 3.8)	**10.1 (6.4, 13.9)**
Italy	**63.4**	29.7	8.6	5.2	0.5	0.5	3.1	0.6	1.1	6.2	1.3	3.2	**33.7**
	**1.4 (0.3, 3.0)**	-0.9 (-2.8, 1.0)	-1.0 (-5.3, 3.5)	-0.3 (-3.2, 2.8)	1.5 (-11.7, 18.3)	4.6 (-2.8, 12.0)	2.3 (-4.2, 9.3)	5.9 (-6.8, 20.8)	-0.9 (-9.9, 7.4)	-1.1 (-4.4, 2.4)	0.7 (-7.6, 9.6)	-4.6 (-7.9, -1.0)	**4.2 (2.3, 6.7)**
Latvia	**40.1**	33.3	5.2	6.6	1.5	0.2	2.6	0.7	0.5	12.0	1.3	4.3	**6.7**
	**2.4 (1.2, 3.6)**	0.4 (-1.3, 2.1)	2.2 (-1.4, 6.0)	0.2 (-3.1, 3.6)	-1.7 (-8.6, 5.6)	0.6 (-5.8, 7.6)	-0.4 (-6.5, 8.2)	-6.4 (-18.5, 7.6)	8.5 (-5.6, 25.6)	-0.2 (-1.9, 1.5)	-7.9 (-16.3, 1.6)	0.1 (-4.6, 5.2)	**13.0 (8.3, 18.0)**
Lithuania	**46.8**	34.1	5.4	6.9	**1.9**	0.4	3.0	0.5	0.6	11.1	1.2	4.8	**12.8**
	**1.7 (0.1, 3.3)**	0.8 (-0.6, 2.1)	0.7 (-3.7, 5.3)	2.8 (-0.9, 5.1)	**12.6 (6.2, 19.7)**	-3.8 (-13.7, 6.6)	3.0 (-0.1, 6.2)	-1.3 (-18.6, 19.6)	6.1 (-2.5, 15.7)	0.6 (-1.5, 2.7)	-0.1 (-5.2, 5.1)	-0.8 (-4.2, 2.7)	**6.9 (4.5, 9.7)**
Malta	40.0	24.4	7.4	4.3	1.2	1.0	2.7	1.2	**1.1**	7.3	1.6	1.5	15.6
	1.7 (-2.2, 5.7)	0.1 (-5.0, 5.0)	0.6 (-14.6, 14.9)	-9.7 (-18.0, -0.7)	NA	NA	4.7 (-2.6, 14.6)	NA	**2.3 (0.8, 3.3)**	0.9 (-3.7, 5.9)	-7.4 (-12.5, -0.5)	-0.4 (-8.5, 7.8)	4.2 (-0.2, 8.8)
Netherlands	**25.6**	21.3	**8.7**	2.4	1.7	0.4	1.5	0.4	1.0	3.8	1.1	1.6	**4.3**
	**1.1 (0.2, 2.0)**	0.6 (-0.2, 1.3)	**1.9 (0.9, 3.0)**	-0.6 (-2.3, 1.1)	-2.4 (-6.6, 1.6)	-0.2 (-5.7, 5.5)	3.8 (0.0, 7.7)	4.5 (-1.9, 11.4)	0.4 (-4.8, 5.9)	-2.7 (-4.4, -0.9)	0.6 (-2.9, 4.3)	0.4 (-3.1, 4.0)	**4.0 (2.0, 6.0)**
Norway	33.6	26.0	9.2	4.6	0.7	0.5	**1.9**	0.4	0.8	6.2	1.2	1.3	**7.6**
	0.6 (-0.5, 1.8)	-0.3 (-1.5, 1.0)	1.0 (-2.1, 4.2)	1.3 (-3.6, 5.4)	-1.2 (-8.0, 6.4)	-0.7 (-8.1, 7.5)	**6.7 (1.4, 12.4)**	4.8 (-14.6, 28.7)	1.4 (-4.6, 7.9)	-2.7 (-5.0, -1.0)	0.1 (-5.2, 5.7)	-5.4 (-10.1, -0.5)	**3.7 (0.8, 6.7)**
Poland	38.8	26.7	5.6	5.8	1.0	0.8	2.1	0.7	0.8	7.8	1.0	2.6	12.1
	1.2 (-1.1, 3.2)	-0.7 (-3.0, 1.8)	0.3 (-3.1, 3.2)	-1.2 (-7.4, 5.3)	NA	0.5 (-8.4, 10.0)	-1.3 (-5.1, 2.9)	0.0 (-14.2, 16.5)	-7.7 (-17.9, 3.1)	0.6 (-4.3, 6.2)	-2.2 (-14.7, 12.2)	5.2 (-9.0, 21.4)	3.3 (-0.7, 7.4)
Slovakia	**39.1**	**32.1**	8.6	6.3	2.6	0.7	2.6	0.7	0.6	8.3	1.2	2.8	**7.1**
	**2.3 (0.4, 3.4)**	**1.0 (0.3, 1.6)**	1.5 (-0.5, 3.5)	1.1 (-1.2, 3.5)	-4.7 (-20.1, 12.7)	-8.3 (-16.8, 1.1)	4.3 (-0.3, 9.1)	3.5 (-2.8, 10.4)	2.9 (-2.4, 7.3)	0.0 (-1.9, 1.9)	3.1 (-4.7, 10.6)	0.7 (-2.8, 4.4)	**8.0 (2.5, 13.7)**
Slovenia	**36.2**	26.5	7.0	4.8	1.3	0.6	1.9	0.3	0.7	6.9	1.5	3.0	**9.7**
	**2.0 (0.4, 3.4)**	0.6 (-0.6, 1.8)	2.1 (-1.1, 5.4)	-0.6 (-3.5, 2.4)	-1.3 (-17.7, 16.4)	-0.9 (-16.0, 17.9)	0.0 (-6.6, 7.1)	0.7 (-11.3, 11.5)	8.8 (-1.9, 21.2)	0.3 (-2.4, 3.1)	-4.7 (-10.6, 1.5)	0.4 (-4.7, 5.7)	**7.0 (4.1, 10.2)**
Spain	35.4	24.4	7.7	4.1	1.7	0.3	1.7	0.6	0.5	5.7	1.1	2.3	**10.9**
	1.0 (-0.5, 2.4)	-1.1 (-2.8, 0.6)	-0.9 (-4.1, 2.3)	-1.2 (-4.9, 2.6)	5.0 (-1.7, 10.7)	-2.2 (-7.4, 3.5)	0.2 (-3.2, 3.7)	0.4 (-10.5, 12.6)	3.7 (-3.1, 11.1)	-2.2 (-4.0, -0.3)	0.5 (-5.7, 7.1)	-2.1 (-5.9, 1.9)	**5.9 (3.3, 8.2)**
Sweden	25.4	19.6	6.4	2.5	0.5	0.4	1.3	0.6	0.6	5.6	**0.9**	1.2	**5.8**
	0.9 (-0.3, 2.1)	-0.2 (-1.1, 0.7)	1.1 (-0.8, 2.9)	2.8 (-0.1, 5.7)	-0.8 (-11.0, 11.2)	2.5 (-2.9, 8.4)	2.3 (-0.7, 5.6)	3.5 (-11.3, 21.3)	2.1 (-4.3, 8.8)	-4.1 (-6.1, -2.1)	**4.0 (0.1, 8.3)**	-2.0 (-7.4, 3.8)	**4.6 (2.8, 6.5)**
Switzerland	**32.2**	20.8	7.4	3.2	1.2	0.3	1.3	0.6	0.9	3.9	1.0	2.0	**11.4**
	**3.2 (0.8, 5.7)**	1.5 (-1.2, 4.2)	2.4 (-0.7, 5.8)	0.6 (-2.8, 4.0)	-3.4 (-15.1, 9.3)	0.9 (-14.8, 20.6)	1.8 (-2.9, 6.9)	2.3 (-7.9, 13.5)	7.6 (-3.0, 20.1)	-2.6 (-5.7, 1.9)	3.5 (-1.8, 8.6)	0.4 (-4.2, 5.5)	**6.5 (4.2, 8.9)**
Ukraine	**44.3**	35.4	7.0	**8.6**	1.4	0.3	2.6	0.6	0.4	**10.3**	1.2	4.4	**9.0**
	**1.3 (0.8, 1.7)**	0.2 (-0.2, 0.5)	0.0 (-1.0, 0.9)	**0.9 (0.3, 1.6)**	5.2 (-2.0, 12.8)	-0.6 (-3.8, 2.0)	1.5 (-0.3, 3.3)	-1.2 (-4.4, 0.8)	-1.2 (-5.9, 3.7)	**1.1 (0.2, 1.9)**	1.7 (-0.4, 3.9)	-3.6 (-4.2, -3.2)	**5.3 (3.8, 6.8)**
United Kingdom – England and Wales	**26.2**	**20.7**	**6.8**	**3.1**	1.7	0.2	**1.6**	**0.5**	**0.7**	5.4	**0.9**	1.0	**5.5**
	**3.3 (2.6, 3.7)**	**2.3 (1.5, 2.8)**	**3.0 (2.0, 3.9)**	**3.5 (2.3, 4.6)**	-1.5 (-4.1, 1.2)	0.9 (-1.2, 4.4)	**3.9 (2.6, 5.3)**	**4.0 (2.1, 6.3)**	**2.4 (0.8, 4.8)**	0.0 (-0.7, 0.7)	**2.2 (0.8, 3.8)**	0.4 (-2.5, 3.4)	**7.2 (6.8, 7.7)**
United Kingdom – Northern Ireland	29.7	24.7	8.2	4.8	1.8	**0.4**	1.6	0.5	0.6	5.9	0.9	1.4	**4.9**
	1.7 (-0.4, 3.9)	0.7 (-1.8, 3.2)	1.4 (-2.7, 5.7)	2.0 (-1.1, 5.1)	-3.8 (-6.4, 15.1)	**7.6 (0.7, 15.3)**	2.9 (-5.2, 11.8)	0.3 (-16.0, 22.1)	0.5 (-10.9, 13.5)	-0.6 (-7.8, 4.8)	-0.6 (-9.4, 9.2)	3.8 (-1.1, 8.9)	**7.4 (1.0, 14.2)**
United Kingdom – Scotland	**27.9**	**22.2**	**8.0**	2.8	2.1	0.3	**1.9**	0.5	0.5	5.2	1.0	1.4	**5.7**
	**2.5 (1.8, 3.3)**	**2.1 (1.5, 2.7)**	**3.2 (1.5, 5.0)**	0.4 (-2.5, 3.5)	-2.0 (-8.1, 4.7)	6.5 (-7.6, 22.6)	**6.3 (1.1, 11.8)**	3.1 (-5.9, 12.8)	2.3 (-6.5, 12.1)	0.7 (-2.3, 3.8)	-0.2 (-3.9, 4.9)	-1.6 (-6.1, 3.3)	**4.3 (1.0, 7.7)**
Oceania													
Australia	**40.6**	**26.6**	**11.1**	**4.0**	1.1	0.3	2.5	**0.6**	1.0	4.5	1.0	1.4	**14.0**
	**2.4 (2.0, 2.7)**	**1.4 (0.9, 1.9)**	**1.8 (0.7, 2.9)**	**1.6 (0.3, 3.0)**	-1.8 (-7.6, 4.4)	2.4 (-2.0, 7.0)	2.1 (0.0, 4.3)	**4.2 (0.2, 8.4)**	3.0 (-1.0, 7.2)	0.4 (-0.8, 1.5)	1.7 (-0.2, 3.7)	-1.2 (-3.6, 1.3)	**4.1 (3.3, 4.9)**
New Zealand	**35.3**	**27.1**	9.7	**5.2**	1.0	0.3	2.2	0.7	0.9	4.8	1.0	2.1	**8.2**
	**2.2 (1.3, 3.2)**	**1.7 (0.2, 3.3)**	0.6 (-1.0, 2.2)	**6.1 (3.8, 8.4)**	8.1 (-3.0, 20.4)	3.6 (-11.0, 20.1)	0.6 (-2.5, 3.9)	-1.1 (-6.5, 4.7)	2.4 (-3.2, 8.4)	-0.7 (-3.2, 1.9)	4.8 (-1.6, 11.8)	1.1 (-3.1, 4.0)	**3.9 (0.8, 7.2)**

[*] Average age-standardized cancer incidence rates (ASRs) between 2000 and 2010 were calculated.
[#] Average annual percentage changes (AAPCs) with 95% confidence intervals (CIs) in cancer incidence among 20-49-year-old adults during the period of 2000-2012 were calculated using the Joinpoint Regression Program (version 4.9.0.1). A maximum of two joinpoints were permitted in this analysis.
Bold numbers denote statistically significant positive AAPCs.
Abbreviations: AAPC, Average annual percentage change; ASR, age-standardized cancer incidence rate; CI, confidence interval.

Supplementary Table S3. Incidence trends of individual early-onset obesity-related cancers among males by countries in 2000-2012.

Country	All obesity-related cancers	All obesity-related cancers excluding thyroid cancer	Colorectum	Esophagus	Gallbladder and extrahepatic bile duct	Kidney	Liver	Multiple Myeloma	Pancreas	Stomach	Thyroid
	ASR/100,000* AAPC (95% CI)#	ASR/100,000 AAPC (95% CI)	ASR/100,000 AAPC (95% CI)	ASR/100,000 AAPC (95% CI)	ASR/100,000 AAPC (95% CI)	ASR/100,000 AAPC (95% CI)	ASR/100,000 AAPC (95% CI)	ASR/100,000 AAPC (95% CI)	ASR/100,000 AAPC (95% CI)	ASR/100,000 AAPC (95% CI)	ASR/100,000 AAPC (95% CI)
Africa											
Uganda	19.9	19.5	3.8	5.7	0.2	0.5	**6.0**	0.7	1.2	2.2	0.6
	2.8 (-0.3, 6.1)	1.3 (-1.0, 4.4)	-1.1 (-7.9, 6.3)	3.9 (-2.1, 10.8)	NA	-2.3 (-27.5, 38.8)	**6.7 (1.8, 11.8)**	0.6 (-21.1, 27.3)	4.6 (-5.7, 16.3)	-2.8 (-16.2, 13.8)	-2.2 (-14.4, 11.6)
Asia											
China	36.8	32.2	8.7	1.4	0.4	**3.1**	11.6	0.4	1.3	5.3	**4.6**
	0.6 (0.0, 1.1)	-1.7 (-2.2, -1.3)	1.3 (-0.6, 3.3)	-5.4 (-7.5, -3.3)	0.7 (-3.8, 5.4)	**7.4 (5.8, 9.1)**	-5.4 (-7.0, -3.0)	-2.0 (-5.3, 1.5)	-0.3 (-5.7, 5.3)	-5.4 (-6.7, -4.2)	**17.9 (14.6, 20.6)**
India	16.0	15.1	3.9	2.3	0.6	0.6	1.4	0.3	0.8	5.2	0.9
	0.1 (-2.0, 2.3)	0.1 (-2.0, 2.4)	2.8 (-0.7, 6.5)	-10.2 (-17.0, -1.3)	4.4 (-3.5, 13.0)	-0.8 (-8.0, 7.0)	1.4 (-6.8, 10.3)	4.1 (-0.3, 10.4)	5.6 (-2.3, 14.4)	-1.0 (-4.9, 3.1)	1.3 (-9.6, 13.5)
Israel	24.2	19.4	7.9	0.4	0.3	4.2	0.7	1.2	1.8	2.8	**4.9**
	0.4 (-0.8, 1.7)	-0.5 (-2.0, 0.9)	-0.3 (-1.7, 1.2)	-4.5 (-9.5, 1.0)	-9.1 (-17.5, 0.1)	0.7 (-2.2, 3.7)	0.0 (-9.3, 10.2)	-0.4 (-3.7, 3.0)	-2.7 (-7.9, 2.9)	-1.5 (-4.1, 1.4)	**4.7 (1.2, 8.3)**
Japan	35.0	33.3	10.5	1.6	0.7	**2.9**	4.4	0.3	1.7	11.2	1.7
	-2.0 (-3.3, -0.8)	-2.5 (-3.3, -1.6)	-1.3 (-2.4, 0.0)	-3.5 (-7.7, 1.0)	-2.6 (-7.4, 2.5)	**6.5 (4.1, 8.9)**	-6.6 (-8.1, -5.1)	-1.7 (-4.5, 2.4)	-0.4 (-2.6, 2.1)	-5.0 (-7.6, -2.2)	6.8 (-0.3, 14.6)
Kuwait	15.8	12.5	6.4	0.6	0.5	2.5	1.2	1.1	1.5	1.3	3.4
	2.2 (-1.8, 6.6)	3.2 (-1.7, 8.4)	6.3 (-1.9, 15.2)	-0.9 (-7.6, 6.6)	NA	7.3 (-2.7, 18.4)	-15.7 (-22.0, -7.6)	5.7 (-18.7, 36.8)	-8.1 (-23.9, 11.4)	-6.3 (-14.7, 3.2)	-1.4 (-7.0, 4.6)
Republic of Korea	**70.9**	56.4	**12.6**	0.6	1.1	**3.6**	16.7	0.4	1.6	19.8	**14.6**
	**4.2 (3.7, 4.9)**	0.1 (-0.3, 0.6)	**5.1 (4.3, 5.8)**	-2.3 (-5.4, 0.9)	-0.9 (-3.5, 1.9)	**6.6 (5.3, 7.9)**	-3.5 (-4.0, -2.9)	1.2 (-2.0, 4.5)	-0.2 (-1.9, 2.0)	-1.4 (-2.8, 0.4)	**28.4 (26.6, 30.1)**
Thailand	27.2	26.1	**5.1**	**0.8**	0.8	0.6	16.0	0.4	0.8	1.7	1.1
	0.6 (-0.9, 2.2)	0.6 (-0.8, 2.1)	**3.6 (1.9, 5.4)**	**8.7 (4.5, 13.2)**	4.0 (-3.4, 12.1)	6.0 (-2.0, 14.5)	-0.8 (-3.3, 1.8)	5.7 (-3.9, 16.4)	0.2 (-4.3, 5.1)	-1.6 (-4.6, 1.6)	0.4 (-5.1, 6.1)
Turkey	**21.0**	17.5	6.6	0.7	0.5	**2.0**	1.1	**0.7**	1.4	4.4	**3.5**
	**2.6 (1.8, 3.6)**	0.8 (-0.5, 2.2)	0.9 (-0.7, 2.7)	-5.2 (-12.4, 2.5)	6.3 (-6.0, 20.2)	**5.8 (2.9, 8.8)**	-1.5 (-6.3, 3.5)	**4.0 (1.8, 6.8)**	3.1 (-1.1, 7.6)	-1.6 (-4.5, 1.5)	**12.9 (10.2, 15.6)**
South America											
Brazil	24.9	21.9	8.4	2.6	0.7	2.6	1.5	1.0	1.1	4.4	3.0
	-0.3 (-2.7, 2.2)	-2.9 (-4.8, -1.1)	0.7 (-7.1, 7.9)	-7.1 (-13.6, 0.0)	5.2 (-5.5, 17.0)	0.0 (-9.1, 10.4)	1.3 (-9.8, 14.0)	4.2 (-10.0, 21.1)	3.0 (-7.2, 14.5)	-2.2 (-9.2, 5.4)	2.9 (-2.7, 12.3)
Chile	24.8	23.1	4.8	1.9	1.7	4.5	1.6	1.4	2.4	9.5	2.9
	-4.5 (-8.5, -0.3)	-5.0 (-9.1, -0.7)	-0.2 (-10.7, 11.5)	-0.4 (-7.0, 6.4)	1.2 (-9.4, 12.7)	-7.6 (-14.2, -0.9)	5.5 (-10.0, 20.2)	-3.3 (-9.4, 2.4)	-3.2 (-22.5, 22.9)	-12.2 (-22.7, 0.0)	1.0 (-11.5, 17.3)
Colombia	21.3	18.9	5.6	0.7	0.5	1.8	0.8	1.0	0.8	7.9	2.4
	0.5 (-1.6, 2.8)	0.1 (-2.1, 2.3)	0.5 (-2.8, 3.4)	-8.5 (-22.2, 5.1)	2.8 (-6.9, 13.4)	-1.6 (-16.4, 15.8)	-8.6 (-22.8, 9.1)	-2.2 (-14.7, 12.1)	8.1 (-9.0, 28.4)	-1.4 (-6.5, 3.8)	4.4 (-4.7, 14.2)
Ecuador	20.4	16.4	4.5	0.5	1.0	**1.1**	1.0	1.2	0.8	7.4	**4.0**
	3.5 (0.0, 7.2)	1.6 (-2.0, 5.4)	3.0 (-4.1, 10.9)	-4.0 (-11.3, 4.6)	-6.4 (-18.1, 6.8)	**14.8 (6.6, 26.1)**	2.8 (-5.3, 12.0)	3.5 (-3.6, 11.0)	-3.0 (-14.2, 9.7)	0.5 (-2.3, 3.5)	**14.8 (3.4, 27.5)**
Central America											
Costa Rica	18.0	15.4	**4.8**	0.3	0.4	1.4	1.3	0.3	0.8	6.2	**2.6**
	-0.5 (-1.7, 0.8)	-1.9 (-3.1, -0.8)	**3.4 (0.1, 6.4)**	-7.4 (-29.4, 18.4)	-13.5 (-26.2, 2.0)	-0.1 (-8.7, 9.5)	-6.5 (-12.9, 0.4)	-2.2 (-13.6, 10.9)	-2.7 (-6.7, 1.4)	-3.5 (-5.2, -1.8)	**9.3 (3.6, 15.3)**
North America											
Canada	**25.1**	**20.2**	**9.1**	1.0	**0.3**	**4.4**	1.3	0.9	**1.4**	1.9	**4.9**
	**2.5 (1.9, 3.1)**	**1.6 (0.9, 2.3)**	**1.8 (1.0, 2.6)**	1.9 (-0.3, 4.2)	**3.7 (0.5, 6.9)**	**2.8 (1.5, 4.2)**	-2.1 (-4.0, -0.2)	0.8 (-1.9, 3.6)	**2.0 (0.6, 3.6)**	0.1 (-2.5, 2.7)	**6.3 (4.8, 7.9)**
United States of America	**28.5**	**23.8**	**10.3**	0.9	0.3	**5.0**	2.4	**1.2**	1.6	2.0	**4.7**
	**2.1 (1.7, 2.5)**	**1.6 (1.1, 2.0)**	**1.6 (1.1, 2.2)**	-1.6 (-3.1, -0.2)	1.0 (-1.6, 3.6)	**4.1 (3.3, 4.9)**	-0.8 (-2.2, 0.7)	**3.2 (0.3, 6.2)**	0.1 (-1.6, 1.8)	0.6 (-0.9, 2.2)	**5.1 (3.7, 6.5)**
Europe											
Belarus	33.3	**29.0**	**6.2**	1.6	0.3	**7.7**	0.9	0.6	**2.4**	9.2	4.2
	0.4 (-0.3, 1.2)	**0.5 (0.2, 0.8)**	**1.7 (0.1, 3.3)**	0.6 (-1.4, 2.8)	1.2 (-3.4, 5.9)	**2.8 (1.5, 4.1)**	0.7 (-4.4, 5.1)	2.0 (-2.2, 6.5)	**2.5 (0.2, 4.8)**	-2.8 (-3.5, -2.0)	-1.0 (-3.0, 1.4)
Bulgaria	23.8	22.7	8.4	0.8	0.4	3.6	1.9	0.6	3.0	4.0	1.1
	-0.1 (-2.1, 2.0)	-0.3 (-2.0, 1.4)	-2.1 (-4.0, -0.7)	0.9 (-2.7, 4.8)	4.1 (-1.0, 9.5)	2.9 (-0.1, 6.0)	-0.2 (-5.9, 5.8)	2.2 (-8.4, 14.3)	-1.0 (-4.1, 2.2)	-2.8 (-5.4, -0.1)	4.6 (-0.4, 9.8)
Croatia	29.2	25.7	10.1	1.4	0.5	4.7	1.4	0.7	2.5	4.3	**3.6**
	-2.6 (-3.7, -0.6)	-3.4 (-4.7, -1.3)	-3.7 (-4.9, -1.7)	-6.4 (-9.1, -2.6)	2.7 (-8.4, 12.8)	-0.3 (-3.1, 2.5)	-2.2 (-7.2, 3.1)	-6.4 (-12.1, -0.3)	-3.2 (-9.7, 3.7)	-3.9 (-6.3, -1.5)	**5.3 (1.2, 9.6)**
Czech Republic	26.9	24.7	9.4	1.6	0.5	6.6	1.0	0.6	2.3	2.6	**2.2**
	-1.1 (-2.4, 0)	-1.8 (-2.7, -0.9)	-2.4 (-3.8, -1.0)	-2.2 (-6.1, 1.9)	-8.6 (-12.9, -4.0)	-0.5 (-2.0, 1.0)	-6.0 (-9.2, -2.5)	-0.4 (-8.5, 8.3)	-1.3 (-3.0, 0.5)	-2.2 (-4.9, 0.5)	**6.8 (4.0, 9.7)**
Denmark	19.4	17.7	7.7	1.2	0.3	**3.3**	0.9	0.7	1.7	1.9	**1.8**
	1.2 (0.0, 2.5)	0.7 (-0.5, 1.9)	0.5 (-0.6, 1.6)	-4.4 (-10.0, 1.9)	-2.6 (-11.3, 7.0)	**5.9 (2.9, 9.1)**	-3.8 (-10.7, 3.8)	0.2 (-5.0, 5.6)	-0.7 (-4.9, 3.6)	-1.9 (-6.8, 3.3)	**3.8 (0.6, 9.6)**
Estonia	25.1	24.1	5.5	1.1	0.6	5.6	0.9	1.1	2.6	6.9	1.1
	-0.2 (-2.3, 2.0)	-0.5 (-3.3, 2.4)	1.6 (-3.5, 7.0)	-1.9 (-12.1, 9.3)	4.8 (-7.9, 18.6)	-0.2 (-5.8, 5.6)	3.5 (-4.7, 12.3)	4.3 (-2.5, 12.0)	-4.0 (-9.2, 1.5)	-2.9 (-5.9, 0.3)	4.3 (-7.5, 18.1)
France	26.5	22.5	8.1	1.9	0.3	5.0	2.2	1.0	**1.9**	2.2	**3.9**
	0.6 (-0.2, 1.4)	0.2 (-0.7, 1.2)	-0.9 (-2.9, 1.2)	-5.9 (-7.4, -4.4)	1.1 (-13.9, 17.5)	2.7 (-0.5, 6.0)	3.1 (-0.1, 6.5)	1.4 (-2.5, 5.4)	**3.9 (0.3, 7.8)**	-0.3 (-3.2, 2.6)	**2.9 (0.5, 5.4)**
Germany	23.1	21.3	8.4	1.4	0.5	4.0	1.5	1.0	1.6	2.9	1.8
	1.1 (-0.3, 2.6)	0.9 (-0.5, 2.4)	2.0 (0.0, 4.1)	0.0 (-3.8, 4.1)	5.0 (-2.6, 13.5)	-1.2 (-4.9, 2.6)	4.3 (-2.5, 11.8)	1.9 (-4.8, 9.2)	-3.2 (-6.9, 0.2)	-2.4 (-4.8, 0.0)	2.8 (-1.8, 7.8)
Iceland	19.0	15.8	6.2	1.7	1.4	5.6	0.7	1.3	1.9	2.1	4.2
	1.9 (-4.9, 9.2)	3.4 (-1.3, 8.5)	4.3 (-10.3, 21.4)	2.2 (-7.1, 12.5)	0.1 (-10.6, 10.5)	0.6 (-6.3, 7.9)	6.0 (-9.4, 26.2)	-0.6 (-1.6, 0.6)	0.2 (-10.0, 11.6)	4.8 (-10.3, 25.8)	-0.3 (-24.2, 26.7)
Ireland	21.3	20.0	8.5	1.7	0.3	**3.6**	**0.9**	1.1	1.4	2.4	**1.4**
	1.7 (-0.1, 3.7)	1.3 (-0.8, 3.7)	1.3 (-2.0, 4.8)	-2.7 (-7.5, 2.3)	0.4 (-11.6, 14.5)	**4.9 (1.4, 8.5)**	**8.1 (2.9, 14.8)**	2.0 (-4.9, 8.7)	-1.2 (-8.3, 6.6)	-1.3 (-5.0, 2.6)	**8.0 (3.3, 13.0)**
Italy	**32.8**	24.0	9.0	0.5	0.5	5.2	2.9	1.2	1.7	2.9	**8.7**
	**1.2 (0.5, 2.0)**	-0.2 (-1.5, 1.2)	-0.6 (-2.8, 1.7)	3.0 (-4.6, 11.2)	2.8 (-7.0, 14.2)	3.1 (-1.3, 8.5)	2.7 (-1.0, 6.1)	-0.5 (-7.0, 6.3)	2.2 (-2.9, 7.9)	-4.4 (-8.4, -0.1)	**6.2 (1.5, 11.1)**
Latvia	24.5	23.1	5.2	1.5	0.5	5.3	1.2	**0.6**	3.0	6.0	**1.5**
	0.7 (-0.5, 1.6)	0.1 (-1.1, 0.9)	2.4 (-2.1, 5.6)	1.0 (-5.4, 5.4)	-10.8 (-23.6, 5.4)	2.4 (-0.6, 5.7)	2.3 (-4.1, 9.1)	**11.8 (6.0, 19.9)**	-0.9 (-6.9, 5.4)	-4.6 (-7.0, -2.2)	**11.0 (3.8, 18.7)**
Lithuania	28.4	25.9	5.7	1.9	0.3	**6.2**	**1.0**	**0.9**	2.9	7.0	**2.5**
	0.9 (-0.5, 1.6)	0.3 (-1.1, 1.8)	1.4 (-0.5, 3.4)	-1.0 (-3.5, 3.2)	-2.9 (-19.1, 16.0)	**2.8 (0.7, 4.9)**	**8.1 (2.0, 14.8)**	**10.2 (2.9, 18.2)**	-3.6 (-6.1, -1.1)	-1.4 (-3.9, 1.2)	**7.8 (3.4, 12.4)**
Malta	18.7	14.8	6.4	1.2	1.2	3.7	1.6	1.9	1.8	2.1	4.2
	6.0 (-0.8, 13.3)	3.9 (-2.8, 11.0)	0.9 (-9.7, 12.7)	6.4 (-3.7, 17.0)	NA	4.2 (-5.8, 15.4)	6.4 (-1.5, 15.5)	6.0 (-1.9, 14.4)	0.2 (-6.8, 6.6)	4.5 (-11.7, 25.6)	12.2 (-5.9, 34.7)
Netherlands	**20.4**	**19.0**	**8.6**	1.7	0.4	**2.9**	0.6	1.3	1.4	2.1	**1.4**
	**1.7 (1.1, 2.3)**	**1.4 (0.9, 2.0)**	**2.1 (1.4, 2.8)**	-0.3 (-3.6, 3.0)	-0.8 (-4.5, 3.8)	**3.9 (2.7, 5.2)**	2.2 (-0.7, 5.2)	0.2 (-1.8, 2.3)	0.6 (-3.5, 4.8)	-1.6 (-3.4, 0.2)	**5.0 (1.0, 9.2)**
Norway	**19.9**	**17.8**	8.2	0.7	0.3	3.9	**0.8**	1.0	1.3	1.4	**2.2**
	**2.0 (0.9, 3.1)**	**1.8 (0.6, 3.0)**	0.6 (-1.3, 2.7)	4.1 (-0.7, 9.3)	-1.5 (-10.8, 9.0)	3.3 (-1.2, 8.1)	**6.1 (1.0, 11.6)**	0.6 (-5.2, 6.9)	0.6 (-4.7, 6.2)	3.1 (-1.2, 7.6)	**4.2 (0.4, 8.2)**
Poland	20.1	18.6	6.3	1.0	0.4	4.0	0.8	0.7	1.8	3.9	1.5
	0.5 (-0.8, 1.9)	0.1 (-2.0, 2.3)	1.0 (-3.3, 5.5)	-6.9 (-20.5, 8.8)	0.1 (-7.5, 7.8)	-1.4 (-7.0, 4.6)	5.3 (-4.2, 15.7)	-3.8 (-14.2, 7.5)	-5.0 (-11.5, 2.0)	2.9 (-2.6, 8.7)	6.9 (-2.9, 18.0)
Slovakia	29.0	27.3	10.3	2.6	0.4	**5.3**	1.1	0.8	2.6	4.1	1.8
	1.1 (-0.2, 2.4)	0.7 (-0.1, 1.5)	1.5 (0.0, 3.1)	-6.9 (-11.9, -1.5)	-8.8 (-16.3, 1.9)	**5.2 (1.5, 9.1)**	0.4 (-4.3, 5.3)	4.8 (-1.4, 11.5)	0.4 (-3.8, 4.9)	-2.0 (-3.5, -0.6)	6.7 (-0.5, 14.4)
Slovenia	25.6	23.1	9.0	1.3	0.5	4.1	0.9	0.9	2.1	4.3	2.5
	0.2 (-2.1, 2.5)	-0.1 (-2.7, 2.5)	0.2 (-2.6, 3.1)	-5.9 (-13.8, 2.9)	-4.2 (-12.0, 4.5)	3.9 (-1.2, 9.3)	-7.2 (-14.1, 0.4)	-0.6 (-10.0, 10.3)	-4.7 (-10.1, 0.9)	1.7 (-2.4, 6.0)	2.9 (-0.2, 6.3)
Spain	25.5	22.9	8.7	1.7	0.4	3.6	**2.7**	0.7	1.9	3.1	**2.6**
	-0.2 (-1.6, 1.2)	-0.9 (-2.2, 0.4)	-0.5 (-3.0, 2.2)	-7.4 (-10.5, -4.1)	-0.8 (-12.0, 12.3)	0.3 (-1.9, 1.9)	**6.1 (2.1, 10.1)**	-0.7 (-5.5, 4.5)	-2.2 (-7.2, 3.1)	-3.4 (-6.5, -0.2)	**6.3 (2.5, 10.2)**
Sweden	**15.3**	**13.7**	**6.5**	0.5	0.4	**2.4**	0.8	0.8	1.0	1.3	**1.6**
	**2.6 (1.6, 3.6)**	**1.9 (0.5, 3.4)**	**2.6 (1.1, 4.1)**	-0.7 (-6.9, 5.8)	7.0 (-0.7, 15.2)	**3.6 (1.2, 6.3)**	2.6 (-2.3, 7.8)	-2.7 (-5.6, -0.2)	0.8 (-4.5, 6.5)	-3.3 (-6.9, 0.4)	**9.3 (4.5, 14.2)**
Switzerland	22.8	19.5	7.2	1.2	0.4	3.3	2.4	0.9	1.5	2.6	3.3
	-0.4 (-2.0, 1.3)	-0.8 (-2.9, 1.4)	0.3 (-2.4, 3.1)	-6.5 (-16.8, 5.0)	-2.4 (-13.3, 10.5)	1.4 (-1.0, 3.8)	-2.1 (-6.0, 0.2)	-0.5 (-10.1, 10.3)	2.5 (-4.1, 9.9)	-1.6 (-6.1, 3.2)	2.8 (-3.4, 9.3)
Ukraine	27.8	25.9	6.8	1.4	0.3	**4.8**	1.0	0.5	3.5	7.4	**1.9**
	-0.7 (-1.1, -0.3)	-1.2 (-1.6, -0.8)	0.1 (-0.7, 0.8)	1.0 (-0.5, 2.7)	3.3 (-2.3, 9.0)	**1.3 (0.4, 2.3)**	-1.8 (-6.3, 2.9)	0.8 (-5.0, 6.8)	-1.4 (-2.2, -0.7)	-4.4 (-5.5, -3.3)	**5.7 (4.2, 7.3)**
United Kingdom – England and Wales	**18.6**	**17.1**	**7.2**	1.7	0.2	**3.0**	1.0	1.0	1.3	1.7	**1.5**
	**2.4 (1.9, 2.8)**	**1.8 (1.4, 2.2)**	**2.5 (1.7, 3.3)**	-0.5 (-2.3, 1.3)	4.7 (-0.7, 9.3)	**3.9 (3.0, 4.9)**	2.2 (-0.6, 5.1)	2.9 (0.0, 5.9)	0.3 (-1.0, 1.9)	-2.3 (-3.9, -0.5)	**7.5 (5.9, 9.2)**
United Kingdom – Northern Ireland	19.9	18.7	9.0	1.8	0.5	**2.5**	0.7	1.1	1.4	1.9	1.2
	2.2 (-0.7, 5.3)	2.4 (-0.5, 5.4)	4.3 (-1.9, 10.8)	-1.6 (-10.3, 8.3)	3.7 (-15.3, 29.1)	**6.4 (3.2, 9.7)**	4.9 (-7.0, 18.4)	-3.7 (-17.7, 12.6)	3.6 (-7.6, 16.1)	-8.6 (-15.3, -1.4)	-0.8 (-7.3, 6.5)
United Kingdom - Scotland	19.9	18.4	7.9	2.1	0.3	**3.2**	**0.9**	1.0	1.3	1.8	1.5
	0.8 (-0.1, 1.8)	0.6 (-0.5, 1.7)	0.2 (-1.6, 2.0)	0.1 (-3.4, 3.6)	-3.0 (-13.7, 9.0)	**3.3 (1.3, 5.4)**	**5.8 (0.7, 11.2)**	5.0 (-2.7, 11.6)	-2.4 (-6.8, 2.2)	-2.0 (-5.7, 1.8)	4.2 (-0.7, 9.3)
Oceania											
Australia	**27.2**	**23.5**	**11.2**	1.1	0.3	**4.5**	**1.7**	1.3	1.5	1.9	**3.6**
	**1.6 (0.7, 2.4)**	**1.1 (0.8, 1.5)**	**1.4 (0.7, 2.4)**	-0.1 (-1.8, 1.7)	-1.8 (-8.2, 5.0)	**3.8 (1.8, 5.7)**	**3.5 (1.2, 5.2)**	0.6 (-1.5, 4.1)	1.9 (-0.4, 4.2)	-0.9 (-3.9, 2.2)	**5.4 (3.2, 6.8)**
New Zealand	**23.2**	**20.7**	**8.8**	1.0	0.2	**3.6**	2.0	1.4	1.3	2.5	**2.4**
	**2.6 (0.6, 4.6)**	**2.4 (0.4, 4.4)**	**3.5 (0.1, 6.0)**	-0.7 (-7.2, 6.4)	2.2 (-11.2, 18.3)	**4.5 (2.1, 7.1)**	0.3 (-5.5, 6.5)	2.5 (-1.3, 6.6)	-0.6 (-6.5, 7.9)	1.9 (-1.5, 5.5)	**4.4 (0.8, 8.3)**

* Average age-standardized cancer incidence rates (ASRs) between 2000 and 2012 were calculated.
\# Average annual percentage changes (AAPCs) with 95% confidence intervals (CIs) in cancer incidence among 20-49-year-old adults during the period of 2000-2012 were calculated using the Joinpoint Regression Program (version 4.9.0.1). A maximum of two joinpoints were permitted in this analysis.
Bold numbers denote statistically significant positive AAPCs.
Abbreviations: AAPC, Average annual percentage change; ASR, age-standardized cancer incidence rate; CI, confidence interval.

Supplementary Table S4. Incidence trends of individual early-onset non-obesity-related cancers among females by countries in 2000-2012.

Country	All non-obesity-related cancers	Bladder	Brain CNS	Breast	Cervix Uteri	Hodgkin Lymphoma	Kaposi Sarcoma	Larynx	Leukemia	Lips, oral cavity, and pharynx	Lung	Melanoma of Skin	Non-Hodgkin Lymphoma
	ASR/100,000* AAPC (95% CI)#	ASR/100,000 AAPC (95% CI)	ASR/100,000 AAPC (95% CI)	ASR/100,000 AAPC (95% CI)	ASR/100,000 AAPC (95% CI)	ASR/100,000 AAPC (95% CI)	ASR/100,000 AAPC (95% CI)	ASR/100,000 AAPC (95% CI)	ASR/100,000 AAPC (95% CI)	ASR/100,000 AAPC (95% CI)	ASR/100,000 AAPC (95% CI)	ASR/100,000 AAPC (95% CI)	ASR/100,000 AAPC (95% CI)
Africa													
Uganda	98.0	0.6	1.0	26.0	50.0	1.7	33.9	0.4	**1.1**	2.6	1.5	0.7	5.3
	-1.7 (-3.8, 0.5)	1.1 (-16.3, 22.1)	4.4 (-8.7, 19.5)	-0.1 (-3.6, 5.9)	-0.5 (-2.6, 1.7)	-1.9 (-17.1, 17.4)	-6.8 (-9.8, -3.7)	-6.3 (-24.0, 14.8)	**14.3 (4.5, 25.1)**	-2.4 (-10.2, 6.3)	-9.5 (-21.9, 4.9)	12.1 (-6.4, 33.7)	-1.7 (-7.5, 4.6)
Asia													
China	**42.6**	0.4	2.5	**43.9**	**7.9**	**0.6**	0.0	0.1	2.5	5.5	5.5	0.3	2.9
	**1.4 (0.9, 1.8)**	-4.9 (-7.7, -1.9)	-1.7 (-3.0, -0.4)	**1.6 (1.0, 2.2)**	**3.6 (1.7, 5.7)**	**4.9 (2.6, 7.3)**	NA	-1.1 (-15.2, 15.6)	-0.6 (-2.0, 0.9)	-1.6 (-2.7, -0.5)	0.8 (-0.5, 2.2)	4.3 (-4.3, 13.7)	0.8 (-1.0, 2.6)
India	29.7	0.2	1.7	27.3	15.1	0.7	NA	0.3	2.3	5.4	1.9	0.2	1.9
	-1.1 (-3.3, 0.9)	0.7 (-8.5, 11.0)	-3.3 (-6.8, -0.4)	0.8 (-0.4, 2.2)	-6.5 (-8.8, -4.1)	10.4 (-2.8, 22.5)	NA	-2.3 (-8.3, 4.5)	5.7 (0.0, 11.8)	1.0 (-3.3, 5.4)	2.1 (-2.7, 8.5)	3.5 (-9.1, 18.2)	2.0 (-1.2, 5.3)
Israel	41.8	1.0	2.9	64.2	7.4	5.1	0.1	0.3	3.1	1.7	3.5	8.7	7.9
	-0.7 (-1.4, 0.0)	3.1 (-4.0, 10.9)	0.3 (-2.2, 2.8)	-0.6 (-1.2, 0.1)	-0.5 (-2.6, 1.6)	-0.2 (-2.7, 2.3)	-3.1 (-10.9, 5.0)	3.8 (-5.5, 13.9)	-2.7 (-5.6, 0.4)	0.2 (-3.3, 3.9)	-1.3 (-5.4, 2.1)	-1.4 (-3.3, 0.3)	1.2 (-0.5, 3.2)
Japan	**26.1**	0.5	1.3	**49.7**	**12.4**	**0.4**	NA	0.1	2.7	1.8	**3.6**	0.4	2.8
	**3.3 (2.3, 4.4)**	7.7 (-2.3, 19.2)	3.5 (-2.0, 9.4)	**3.4 (2.4, 4.4)**	**4.0 (2.6, 5.5)**	**8.0 (2.5, 14.1)**	NA	-1.5 (-10.8, 10.5)	-0.3 (-2.3, 1.8)	4.8 (-0.8, 10.7)	**2.9 (0.5, 5.4)**	0.7 (-11.2, 14.8)	0.9 (-1.7, 3.7)
Kuwait	16.2	0.6	1.8	42.9	2.3	**2.1**	NA	NA	3.2	1.5	1.0	0.6	4.1
	0.7 (-2.0, 3.5)	-1.8 (-39.7, 60.0)	1.2 (-11.0, 15.4)	0.9 (-1.6, 3.5)	-4.7 (-14.9, 6.8)	**14.5 (2.8, 27.5)**	NA	NA	-5.8 (-13.7, 2.8)	-2.3 (-15.6, 13.0)	-5.6 (-21.4, 13.8)	-4.0 (-17.0, 12.0)	3.3 (-8.3, 16.5)
Republic of Korea	**28.3**	0.4	1.8	**46.2**	14.2	**0.3**	0.0	0.0	2.7	**1.5**	**3.6**	0.3	**3.4**
	**3.6 (3.3, 3.8)**	-2.6 (-4.8, -0.3)	0.2 (-1.0, 1.5)	**6.0 (5.6, 6.4)**	-2.5 (-4.0, -1.1)	**9.9 (7.0, 13.0)**	-1.6 (-17.7, 16.4)	-8.8 (-19.4, 3.1)	0.1 (-1.8, 1.9)	**3.3 (1.8, 4.6)**	**3.0 (2.0, 4.1)**	0.5 (-2.6, 3.7)	**6.8 (4.2, 9.4)**
Thailand	35.3	0.4	1.7	25.9	20.0	0.5	0.1	0.1	3.0	2.5	4.0	0.2	3.1
	-1.5 (-2.7, -0.3)	-0.4 (-15.9, 17.5)	0.0 (-4.7, 5.0)	1.1 (-0.2, 2.5)	-6.1 (-7.6, -4.5)	6.3 (-5.0, 19.3)	-8.7 (-21.9, 7.0)	-1.4 (-11.6, 10.2)	-0.2 (-3.4, 3.0)	-0.8 (-2.5, 1.0)	0.5 (-1.4, 2.0)	3.6 (-4.4, 12.3)	0.4 (-1.4, 2.2)
Turkey	**23.9**	**1.1**	3.3	**40.6**	**5.0**	1.5	0.1	0.3	**3.3**	1.8	3.4	1.1	3.0
	**2.5 (1.8, 3.3)**	**9.2 (3.5, 15.2)**	2.7 (-1.1, 6.6)	**2.4 (1.2, 3.4)**	**4.3 (2.9, 5.9)**	1.4 (-2.5, 5.5)	0.6 (-12.1, 13.3)	0.5 (-11.8, 15.0)	**1.9 (0.1, 3.8)**	2.4 (-3.0, 7.0)	1.5 (-1.2, 4.2)	4.5 (-1.5, 11.0)	0.2 (-2.9, 3.6)
South America													
Brazil	43.9	0.9	3.5	43.9	20.9	2.0	0.4	0.6	3.5	2.7	**3.0**	3.6	3.4
	-2.7 (-4.6, -0.8)	-2.6 (-10.7, 6.1)	-2.7 (-10.8, 6.2)	-1.0 (-2.0, 0.1)	-6.3 (-9.5, -2.9)	-1.7 (-6.8, 3.6)	NA	3.2 (-11.2, 19.6)	-14.5 (-23.8, -4.0)	1.9 (-4.1, 8.5)	**6.9 (1.0, 13.1)**	0.5 (-7.2, 8.9)	-2.7 (-9.1, 4.2)
Chile	38.1	1.2	2.6	25.3	24.3	1.8	1.3	1.3	3.2	1.5	1.5	2.6	2.9
	-2.7 (-5.6, 0.2)	-0.7 (-4.0, 2.3)	-4.3 (-11.5, 2.6)	-0.9 (-5.4, 3.7)	-3.2 (-11.8, 4.4)	-4.4 (-16.1, 9.3)	NA	NA	-1.8 (-10.5, 7.6)	-1.7 (-17.2, 15.4)	-12.0 (-16.4, -5.6)	-4.8 (-15.5, 7.5)	-0.4 (-13.3, 14.7)
Colombia	39.8	0.5	2.8	35.8	21.3	1.3	0.3	0.3	3.7	**1.8**	1.7	1.7	4.8
	-2.0 (-3.1, -0.9)	-0.2 (-12.7, 14.5)	3.1 (-2.3, 8.8)	-1.2 (-2.5, 0.1)	-5.0 (-6.0, -3.9)	0.2 (-7.7, 8.8)	-2.5 (-9.1, 4.6)	-8.2 (-17.9, 3.2)	1.6 (-5.3, 9.0)	**2.9 (0.5, 5.5)**	-2.6 (-7.3, 3.5)	-4.0 (-9.2, 1.5)	-1.8 (-7.1, 3.9)
Ecuador	36.7	0.6	3.1	27.7	18.1	0.7	0.3	0.5	3.9	1.0	2.0	1.6	**5.6**
	1.1 (-0.6, 2.7)	-1.1 (-14.8, 14.7)	0.8 (-4.1, 6.0)	0.9 (-0.8, 2.5)	-0.5 (-4.6, 3.7)	2.0 (-13.8, 21.7)	19.6 (-6.2, 53.1)	-46.8 (-60.9, -27.4)	-0.7 (-7.2, 6.1)	4.6 (-4.7, 15.0)	5.1 (-2.6, 13.3)	1.0 (-4.2, 6.5)	**5.6 (2.5, 8.8)**
Central America													
Costa Rica	32.8	0.4	1.8	28.7	19.2	2.0	0.2	0.2	2.7	1.2	0.9	1.6	2.7
	-0.3 (-2.5, 2.0)	-5.9 (-10.6, -1.8)	-4.0 (-9.6, 1.9)	1.0 (-0.9, 2.9)	-1.5 (-4.1, 1.0)	3.3 (-0.9, 7.8)	-4.9 (-16.3, 8.2)	7.9 (-33.3, 87.2)	-6.0 (-11.9, 0.4)	0.9 (-6.4, 7.3)	0.1 (-8.1, 9.3)	6.8 (-3.0, 15.4)	1.3 (-4.7, 7.7)
North America													
Canada	44.8	1.1	3.1	**52.7**	10.2	3.2	0.0	0.2	**3.1**	2.3	5.8	10.6	5.2
	0.1 (-0.2, 0.5)	-1.7 (-4.7, 1.4)	0.0 (-1.0, 1.0)	**0.6 (0.2, 1.0)**	-0.3 (-1.2, 0.7)	-0.6 (-2.1, 1.4)	-5.3 (-18.8, 10.4)	-0.5 (-4.3, 3.5)	**1.8 (0.8, 2.7)**	0.7 (-0.7, 2.1)	-3.7 (-5.5, -1.9)	0.5 (-0.1, 1.1)	-1.1 (-2.1, -0.1)
United States of America	46.5	1.0	3.1	59.7	9.0	3.4	0.1	0.3	**3.4**	2.4	5.4	13.0	5.6
	-0.3 (-0.5, 0.0)	-2.4 (-4.1, -0.6)	-1.1 (-2.8, 0.6)	0.2 (-0.1, 0.6)	-1.5 (-1.9, -1.0)	-0.2 (-1.0, 0.6)	-8.0 (-13.5, -2.1)	-1.5 (-5.2, 2.2)	**1.7 (0.9, 2.4)**	-0.4 (-1.7, 1.0)	-2.3 (-3.6, -1.1)	-0.3 (-1.7, 1.0)	0.1 (-0.7, 1.0)
Europe													
Belarus	**37.9**	0.6	3.4	35.6	**16.7**	4.3	0.1	0.2	3.0	1.1	1.9	4.3	**2.4**
	**1.4 (0.7, 2.1)**	-1.1 (-6.1, 4.1)	1.2 (-0.7, 3.2)	0.8 (-0.3, 1.9)	**2.2 (1.0, 3.4)**	1.8 (-0.3, 3.5)	6.2 (-5.4, 20.0)	2.8 (-6.2, 12.7)	0.7 (-2.5, 4.1)	1.5 (-1.9, 6.8)	2.4 (-0.9, 5.8)	2.7 (-0.1, 5.6)	**4.1 (0.4, 8.0)**
Bulgaria	**48.8**	**1.3**	3.5	**45.0**	28.3	2.5	0.1	0.3	2.2	**1.7**	**3.9**	**2.7**	**2.4**
	**1.0 (0.7, 1.2)**	**6.4 (1.6, 11.6)**	-0.4 (-2.9, 2.1)	**1.0 (0.8, 1.3)**	-0.7 (-2.4, 1.0)	3.0 (-1.6, 7.9)	NA	0.0 (-6.9, 7.6)	-0.2 (-3.4, 3.1)	**4.6 (1.1, 8.2)**	**2.1 (0.7, 3.5)**	**5.1 (1.5, 8.9)**	**4.1 (2.0, 6.3)**
Croatia	48.4	**1.4**	5.4	45.3	16.0	3.7	NA	0.3	2.8	1.8	5.5	7.0	4.3
	-1.7 (-2.3, -1.1)	**4.9 (2.7, 8.1)**	-4.1 (-6.5, -1.6)	-0.8 (-2.2, 1.7)	-1.9 (-4.0, 0.3)	0.5 (-2.9, 4.0)	NA	-1.4 (-16.5, 11.3)	-7.2 (-12.3, -1.6)	-0.2 (-4.4, 4.2)	-2.2 (-5.2, 0.2)	-0.1 (-2.2, 2.1)	-0.1 (-1.6, 1.2)
Czech Republic	**48.0**	1.1	3.2	**42.0**	19.7	3.0	0.0	0.1	2.2	1.8	3.5	10.4	**3.0**
	**0.5 (0.1, 1.0)**	-4.3 (-7.2, -1.2)	-1.6 (-3.6, 0.5)	**1.9 (1.1, 2.6)**	-1.9 (-3.1, -0.6)	-0.3 (-2.7, 2.2)	0.3 (-8.2, 10.8)	-6.7 (-13.6, -1.0)	0.2 (-1.7, 2.1)	1.7 (-1.0, 4.5)	-2.7 (-4.5, -0.8)	1.9 (-0.1, 3.9)	**2.5 (0.2, 4.9)**
Denmark	**78.8**	1.5	**12.1**	58.0	17.3	2.7	NA	0.3	3.0	2.6	6.9	**28.6**	3.9
	**1.9 (1.3, 2.5)**	-2.2 (-6.5, 2.2)	**4.3 (1.5, 7.1)**	0.5 (-0.4, 1.4)	0.5 (-0.9, 2.0)	0.2 (-2.7, 3.3)	NA	-2.0 (-15.0, 12.9)	0.0 (-3.3, 3.6)	-1.3 (-5.3, 2.9)	-3.2 (-6.3, 0.0)	**4.9 (3.0, 6.9)**	0.8 (-1.7, 3.5)
Estonia	**49.8**	0.6	3.4	38.1	23.0	3.9	0.3	0.4	2.6	1.7	2.5	**9.0**	3.0
	**1.9 (1.1, 2.7)**	0.2 (-8.8, 10.2)	2.2 (-7.2, 12.4)	1.3 (-0.4, 3.0)	-0.3 (-2.9, 2.8)	0.9 (-4.1, 6.2)	NA	4.0 (-4.1, 12.5)	2.4 (-2.2, 7.3)	4.3 (-5.8, 15.7)	1.6 (-5.9, 9.7)	**7.3 (4.0, 10.7)**	0.5 (-5.0, 7.8)
France	**49.3**	0.5	3.1	76.9	10.1	3.7	0.1	0.4	3.1	**3.2**	7.0	**13.4**	4.6
	**0.8 (0.3, 1.4)**	2.2 (-4.9, 9.9)	0.9 (-1.5, 3.4)	1.1 (-0.5, 2.4)	-2.0 (-4.3, 0.4)	0.0 (-4.3, 4.5)	-3.9 (-28.9, 21.4)	-7.0 (-20.0, 8.1)	0.6 (-3.8, 4.0)	**2.9 (0.8, 5.8)**	1.0 (-1.1, 3.1)	**4.2 (2.4, 6.0)**	-0.6 (-1.8, 0.6)
Germany	50.9	1.4	2.9	**59.9**	11.8	3.0	0.2	0.4	3.5	3.0	7.7	12.9	4.3
	0.2 (-0.8, 1.2)	-0.6 (-6.7, 5.9)	-0.2 (-3.7, 3.6)	**1.6 (0.2, 3.1)**	-1.0 (-2.3, 0.8)	2.9 (-1.7, 7.6)	-39.7 (-58.2, -12.6)	-8.2 (-18.9, 4.0)	1.3 (-2.1, 4.8)	-6.1 (-9.8, -2.3)	-2.2 (-4.9, 0.6)	-2.7 (-5.4, 0.2)	-1.2 (-4.2, 1.9)
Iceland	71.9	3.0	11.3	58.0	16.4	3.9	NA	1.4	2.8	2.3	5.8	24.6	3.7
	-0.3 (-2.7, 2.1)	-7.0 (-16.8, 5.1)	3.4 (-4.8, 12.2)	1.1 (-1.4, 3.5)	1.2 (-4.0, 6.8)	0.3 (-17.4, 21.7)	NA	NA	1.1 (-8.4, 11.9)	-6.5 (-23.0, 12.0)	-5.0 (-12.9, 3.9)	-5.2 (-10.5, 0.5)	-2.1 (-14.8, 12.8)
Ireland	**48.3**	1.3	3.0	**61.1**	**15.2**	2.8	0.2	0.3	2.8	**1.8**	4.0	**12.7**	4.4
	**1.3 (0.7, 2.1)**	-0.7 (-6.0, 5.0)	0.5 (-2.9, 4.7)	**1.4 (0.5, 2.3)**	**3.1 (1.3, 5.1)**	0.4 (-6.9, 8.4)	2.9 (-11.7, 20.4)	3.6 (-8.6, 17.9)	1.2 (-2.4, 5.0)	**7.7 (2.0, 13.7)**	0.4 (-2.1, 2.7)	**1.9 (0.6, 3.3)**	-0.2 (-4.9, 4.8)
Italy	**48.0**	2.1	2.9	**74.8**	7.0	4.8	0.2	0.3	3.3	1.9	4.0	**15.1**	6.5
	**0.8 (0.2, 1.3)**	-0.6 (-7.3, 6.7)	0.1 (-8.5, 9.4)	**1.1 (0.7, 1.5)**	-1.4 (-4.5, 1.7)	1.4 (-3.7, 6.8)	-2.0 (-8.7, 5.2)	-1.2 (-17.6, 17.5)	-2.7 (-8.2, 3.2)	-0.5 (-6.1, 5.6)	0.2 (-2.9, 3.5)	**3.9 (0.1, 7.8)**	-1.0 (-2.3, 0.9)
Latvia	**39.5**	0.7	4.7	36.8	**18.3**	2.9	NA	0.2	2.3	1.2	2.5	**4.2**	**2.5**
	**3.1 (1.8, 4.0)**	6.8 (-2.9, 17.8)	2.6 (-0.8, 6.3)	0.8 (-0.6, 2.3)	**5.9 (3.8, 8.1)**	0.6 (-3.3, 4.8)	NA	-3.2 (-20.5, 18.5)	1.4 (-5.4, 7.5)	2.4 (-6.9, 13.1)	4.2 (-1.6, 10.4)	**7.2 (3.6, 11.1)**	**7.2 (2.9, 9.9)**
Lithuania	**50.9**	0.7	3.9	37.9	27.7	**3.6**	NA	0.2	**3.0**	1.3	2.4	5.2	3.0
	**0.8 (0.2, 1.3)**	-1.7 (-8.7, 5.9)	1.3 (-0.8, 3.5)	-0.1 (-1.0, 1.0)	0.0 (-2.4, 2.4)	**1.2 (4.2, 6.8)**	NA	-0.8 (-15.8, 18.5)	**4.8 (0.7, 9.3)**	1.3 (-3.8, 6.9)	3.7 (-3.9, 9.1)	2.6 (-0.9, 6.2)	4.3 (-1.4, 10.4)
Malta	**31.5**	1.5	2.8	**51.1**	6.2	2.9	NA	1.0	2.6	2.2	**3.1**	7.3	**4.6**
	**4.3 (2.4, 6.4)**	-0.6 (-23.5, 26.3)	-0.3 (-17.7, 21.0)	**2.7 (0.7, 4.7)**	-1.5 (-12.7, 11.4)	8.8 (-0.9, 19.7)	NA	0.5 (-0.9, 1.8)	0.4 (-16.2, 19.4)	5.6 (-0.7, 12.4)	**11.1 (0.7, 22.6)**	15.6 (-2.1, 36.3)	**12.6 (3.5, 22.6)**
Netherlands	**56.9**	0.8	3.3	**73.6**	**9.6**	2.9	0.1	0.3	2.9	2.2	8.8	**22.1**	3.9
	**1.2 (0.7, 1.6)**	-0.4 (-4.0, 3.5)	-0.1 (-2.5, 2.3)	**0.8 (0.2, 1.5)**	**2.5 (1.8, 3.3)**	1.6 (-0.6, 3.9)	-2.4 (-17.5, 15.7)	-2.8 (-8.8, 3.7)	-1.7 (-3.5, 0.3)	-0.4 (-2.1, 1.4)	-0.8 (-1.4, -0.1)	**3.0 (1.1, 5.2)**	1.3 (-0.7, 3.4)
Norway	**64.1**	1.1	13.1	52.6	15.6	3.0	NA	0.2	2.7	**1.9**	4.5	**18.3**	3.8
	**1.3 (0.5, 2.0)**	-0.7 (-5.7, 4.7)	2.7 (0.0, 5.4)	0.9 (-0.2, 2.0)	0.9 (-0.5, 2.2)	0.5 (-3.5, 4.7)	NA	2.0 (-11.9, 18.2)	1.3 (-2.9, 5.8)	**4.5 (0.7, 8.5)**	-0.3 (-3.0, 2.6)	**2.2 (1.1, 3.3)**	2.8 (-0.6, 6.2)
Poland	35.7	1.2	4.6	33.9	12.7	2.8	NA	0.8	2.2	1.4	3.8	4.0	2.2
	-1.9 (-3.6, -0.1)	2.2 (-7.1, 12.8)	-7.9 (-11.9, -0.8)	-0.7 (-2.4, 1.6)	-6.4 (-10.9, -1.8)	-2.3 (-6.9, 2.7)	NA	-6.3 (-24.3, 13.6)	0.7 (-4.1, 5.8)	0.5 (-12.8, 15.8)	-7.0 (-14.7, 1.1)	1.2 (-4.2, 7.2)	0.2 (-3.1, 3.6)
Slovakia	**47.1**	1.0	2.9	**36.8**	22.1	3.1	NA	0.3	2.5	1.6	3.3	6.9	3.4
	**1.1 (0.6, 1.6)**	4.0 (-2.9, 11.5)	-0.2 (-7.6, 7.8)	**1.9 (1.4, 2.5)**	-0.6 (-2.1, 0.9)	2.5 (-1.1, 6.3)	NA	-7.0 (-23.5, 14.0)	-0.3 (-4.4, 4.0)	1.4 (-4.8, 8.2)	1.4 (-3.4, 6.5)	0.1 (-3.0, 3.3)	4.6 (-1.7, 11.4)
Slovenia	53.5	0.6	2.6	47.2	18.3	2.8	NA	0.5	2.3	2.3	5.7	**14.9**	3.8
	-0.3 (-1.2, 0.7)	2.0 (-18.7, 27.6)	-1.1 (-8.2, 6.5)	0.6 (-0.8, 1.9)	-7.0 (-9.3, -4.6)	-1.8 (-9.9, 6.9)	NA	-3.9 (-18.5, 12.8)	1.0 (-6.3, 8.8)	-0.7 (-7.0, 5.9)	-2.9 (-5.8, 0.1)	**5.9 (1.0, 11.1)**	2.1 (-2.6, 7.0)
Spain	40.8	1.9	2.9	57.1	8.5	3.3	0.1	0.4	2.9	2.2	5.4	8.1	5.1
	-0.4 (-1.3, 0.4)	0.5 (-4.1, 5.5)	-1.7 (-5.8, 2.7)	0.5 (-0.3, 1.4)	-0.3 (-2.7, 2.2)	2.4 (-1.1, 6.1)	3.7 (-7.2, 16.7)	-0.8 (-6.3, 4.9)	-0.7 (-3.5, 2.2)	-3.4 (-8.4, 1.8)	0.4 (-2.8, 3.7)	-0.5 (-4.0, 3.0)	-1.2 (-4.4, 1.7)
Sweden	**57.3**	1.1	**13.9**	56.1	11.4	2.5	NA	0.1	2.7	2.0	3.4	**16.8**	3.3
	**1.3 (0.7, 2.0)**	-2.9 (-6.4, 0.8)	**2.0 (1.1, 3.0)**	0.8 (-0.1, 1.7)	1.6 (-0.5, 2.9)	1.4 (-1.0, 3.0)	NA	1.1 (-8.8, 11.9)	-0.4 (-3.0, 2.2)	0.8 (-1.2, 2.8)	-3.2 (-6.5, 0.2)	**4.8 (3.6, 5.9)**	0.3 (-2.2, 2.8)
Switzerland	50.0	1.0	2.9	60.3	5.8	3.7	0.4	0.3	2.5	2.6	5.3	21.4	4.4
	0.0 (-1.1, 1.0)	9.2 (-6.2, 23.1)	-1.6 (-7.5, 4.9)	0.5 (-0.7, 1.8)	-2.8 (-5.3, -0.1)	0.4 (-5.7, 6.9)	-2.5 (-18.1, 17.9)	-0.3 (-17.0, 16.6)	0.5 (-4.2, 5.4)	0.7 (-3.7, 5.4)	-0.6 (-3.7, 2.6)	-0.6 (-2.3, 1.1)	-0.1 (-3.6, 3.6)
Ukraine	**44.2**	0.6	3.6	34.6	**22.7**	4.0	0.0	0.2	2.7	1.3	2.6	**4.4**	**2.2**
	**0.8 (0.5, 1.2)**	2.4 (-1.5, 6.2)	0.8 (-1.3, 2.8)	-0.1 (-0.7, 0.4)	**1.8 (0.9, 2.6)**	0.8 (-0.9, 2.5)	14.3 (-9.1, 46.5)	-0.7 (-5.4, 4.2)	-0.3 (-2.5, 1.9)	0.8 (-0.9, 2.6)	-0.4 (-0.9, 0.2)	**2.4 (1.5, 3.5)**	**5.5 (3.5, 8.1)**
United Kingdom – England and Wales	**44.9**	0.6	2.7	**60.7**	**12.4**	2.9	0.1	0.2	2.6	**2.2**	4.0	**13.0**	**4.1**
	**1.0 (0.6, 1.5)**	-1.5 (-3.4, 0.5)	-0.8 (-2.2, 0.2)	**0.9 (0.5, 1.3)**	**2.2 (1.0, 3.5)**	1.3 (-0.1, 3.4)	6.1 (-0.7, 12.9)	0.0 (-3.3, 3.5)	0.9 (-0.2, 2.1)	**2.7 (1.5, 4.0)**	-0.9 (-2.6, 0.9)	**2.7 (1.9, 3.4)**	**1.3 (0.3, 2.4)**
United Kingdom – Northern Ireland	50.8	1.6	2.9	58.0	**16.2**	2.7	NA	0.4	2.7	**2.1**	4.1	**14.2**	4.1
	1.5 (0.0, 3.0)	4.0 (-1.1, 9.5)	-1.2 (-6.7, 2.9)	0.3 (-0.8, 1.5)	**5.0 (0.3, 9.8)**	0.9 (-6.4, 8.8)	NA	-1.3 (-11.5, 9.7)	0.6 (-6.7, 8.5)	**9.2 (3.2, 15.7)**	0.8 (-4.6, 6.6)	**4.1 (1.5, 6.8)**	-2.2 (-7.6, 3.6)
United Kingdom - Scotland	**53.5**	1.6	2.7	**57.1**	**15.2**	3.0	**0.1**	0.4	2.8	2.3	5.4	**15.9**	4.2
	**1.1 (0.6, 1.6)**	3.4 (-1.1, 8.1)	0.3 (-3.3, 4.0)	**1.0 (0.5, 1.5)**	**1.5 (0.3, 2.9)**	-1.8 (-6.0, 2.7)	**7.1 (4.8, 9.6)**	-4.2 (-13.9, 6.8)	-3.1 (-5.8, 0.7)	4.8 (0.0, 9.9)	-2.4 (-6.5, 1.8)	**1.8 (0.2, 3.2)**	2.0 (-1.6, 5.9)
Oceania													
Australia	60.7	0.4	3.2	**62.3**	**8.5**	**2.9**	0.0	0.1	3.5	3.2	4.2	29.5	5.2
	0.0 (-0.3, 0.3)	-3.3 (-10.1, 3.9)	-0.2 (-2.6, 2.4)	**0.5 (0.1, 0.9)**	**1.7 (0.8, 2.4)**	**1.5 (0.2, 2.8)**	-1.1 (-3.1, 0.6)	0.1 (-5.6, 6.3)	1.3 (-0.5, 3.1)	0.5 (-0.6, 1.5)	0.2 (-1.9, 2.2)	-1.9 (-2.6, -1.1)	0.4 (-0.9, 1.8)
New Zealand	60.7	0.7	2.9	**66.8**	10.0	2.4	0.2	0.2	3.8	2.2	4.8	29.4	4.3
	-0.2 (-1.1, 0.6)	-13.2 (-22.9, -1.8)	-0.3 (-4.3, 3.9)	**1.4 (0.6, 2.2)**	-2.3 (-4.0, -0.5)	0.7 (-3.8, 5.5)	NA	-2.4 (-27.4, 31.7)	-2.2 (-5.1, 0.8)	0.1 (-2.7, 3.0)	-0.7 (-3.8, 2.6)	-2.8 (-4.4, -1.2)	1.1 (-1.8, 4.2)

* Average age-standardized cancer incidence rates (ASRs) between 2000 and 2010 were calculated.
\# Average annual percentage changes (AAPCs) with 95% confidence intervals (CIs) in cancer incidence among 20-49-year-old adults during the period of 2000-2012 were calculated using the Joinpoint Regression Program (version 4.9.0.1). A maximum of two joinpoints were permitted in this analysis.
Bold numbers denote statistically significant positive AAPCs.
Abbreviations: AAPC, Average annual percentage change; ASR, age-standardized cancer incidence rate; CI, confidence interval; CNS, central nervous system.

Supplementary Table S5. Incidence trends of individual early-onset non-obesity-related cancers among males by countries in 2000-2012.

Country	All non-obesity-related cancers	Bladder	Brain CNS	Hodgkin Lymphoma	Kaposi Sarcoma	Larynx	Leukemia	Lips, oral cavity, and pharynx	Lung	Melanoma of Skin	Non-Hodgkin Lymphoma	Prostate	Testis
	ASR/100,000[*] AAPC (95% CI)[#]	ASR/100,000 AAPC (95% CI)	ASR/100,000 AAPC (95% CI)	ASR/100,000 AAPC (95% CI)	ASR/100,000 AAPC (95% CI)	ASR/100,000 AAPC (95% CI)	ASR/100,000 AAPC (95% CI)	ASR/100,000 AAPC (95% CI)	ASR/100,000 AAPC (95% CI)	ASR/100,000 AAPC (95% CI)	ASR/100,000 AAPC (95% CI)	ASR/100,000 AAPC (95% CI)	ASR/100,000 AAPC (95% CI)
Africa													
Uganda	60.4	0.7	1.1	1.6	40.1	0.8	1.7	4.6	1.5	0.9	6.4	1.5	0.5
	-2.7 (-6.2, 1.4)	-9.7 (-20.3, 0.9)	2.5 (-13.0, 20.7)	1.4 (-4.2, 7.4)	-5.7 (-25.4, -18.5)	-2.3 (-16.1, 14.1)	14.2 (-2.7, 34.5)	-2.4 (-10.2, 6.3)	8.9 (-6.7, 22.2)	-5.7 (-25.4, 18.5)	0.7 (-9.9, 12.7)	-6.5 (-14.0, 2.1)	-3.6 (-21.1, 19.9)
Asia													
China	35.6	1.3	2.8	0.7	0.1	0.6	3.2	12.7	8.5	0.3	3.2	**0.2**	**2.2**
	-1.1 (-1.5, -0.7)	-7.2 (-9.9, -4.5)	-1.5 (-4.6, 1.8)	-0.4 (-3.6, 3.2)	3.3 (-3.5, 7.8)	-4.5 (-12.2, 3.5)	0.6 (-1.1, 2.1)	-1.6 (-2.7, -0.5)	-2.8 (-3.8, -1.8)	3.3 (-3.5, 7.8)	0.6 (-2.3, 3.5)	**17.9 (11.4, 24.8)**	**3.7 (2.4, 4.6)**
India	29.1	0.6	2.3	1.0	0.1	1.3	3.0	12.8	3.8	0.2	2.8	0.2	1.0
	1.1 (0, 2.1)	-2.8 (-10.5, 5.6)	-2.8 (-6.4, 1.1)	0.5 (-4.9, 6.3)	0.1 (-10.1, 11.1)	0.2 (-8.4, 9.6)	0.4 (-3.4, 4.3)	1.0 (-3.3, 5.4)	-1.1 (-5.3, 3.3)	0.1 (-10.1, 11.1)	-3.3 (-8.2, 1.8)	3.1 (-9.4, 17.1)	-2.2 (-8.8, 5.0)
Israel	57.5	5.3	4.3	5.0	0.4	1.3	4.6	2.8	6.5	7.2	**10.1**	1.6	8.4
	-1.4 (-2.3, -0.6)	-3.9 (-6.8, -0.1)	-0.8 (-3.1, 1.5)	-1.2 (-2.8, 0.5)	-2.5 (-3.6, -1.5)	-1.3 (-5.9, 3.4)	-5.0 (-8.5, -0.6)	0.2 (-3.3, 3.9)	-1.7 (-4.6, 1.4)	-2.5 (-3.6, -1.5)	**1.8 (0.2, 3.7)**	-0.2 (-8.9, 9.3)	0.2 (-1.7, 2.1)
Japan	**26.4**	**2.1**	1.8	0.5	0.1	0.4	3.7	3.3	6.5	0.3	3.5	**0.3**	**3.9**
	**1.4 (0.4, 2.7)**	**2.5 (0.7, 4.4)**	-0.4 (-3.0, 4.0)	5.7 (-1.2, 12.3)	-0.3 (-10.4, 11.1)	-9.1 (-17.1, -0.3)	1.5 (-0.5, 3.6)	4.8 (-0.8, 10.7)	-2.0 (-3.8, -0.2)	-0.3 (-10.4, 11.1)	2.7 (-0.6, 6.1)	**22.2 (8.7, 37.9)**	**6.4 (3.7, 9.2)**
Kuwait	30.8	2.0	2.6	**4.5**	0.7	0.8	4.1	3.0	1.9	0.6	8.7	1.1	3.1
	0.5 (-2.2, 3.4)	2.9 (-6.6, 13.7)	-1.9 (-11.5, 8.9)	**11.2 (2.0, 18.7)**	NA	2.8 (-6.9, 15.7)	0.7 (-7.7, 10.1)	-2.3 (-15.6, 13.0)	-4.3 (-9.2, 0.9)	2.7 (NA, NA)	-3.7 (-10.2, 3.4)	2.8 (-3.6, 10.5)	8.9 (-1.7, 20.9)
Republic of Korea	**22.3**	1.7	**2.3**	**0.5**	0	0.5	**3.3**	**2.7**	5.4	0.3	**4.1**	**0.4**	**1.2**
	**1.3 (0.6, 1.9)**	-1.0 (-3.4, 1.4)	**1.1 (0.2, 2.0)**	**6.7 (1.2, 12.4)**	0 (-4.3, 4.5)	-8.6 (-11.8, -6.0)	**2.1 (0.6, 3.7)**	**3.3 (1.8, 4.6)**	-2.4 (-3.8, -1.0)	0 (-4.3, 4.5)	**3.1 (1.9, 4.3)**	**16.4 (9.4, 23.8)**	**7.0 (4.8, 9.2)**
Thailand	23.5	1.0	1.6	0.5	0.2	0.5	2.9	5.3	6.4	0.2	4.1	0.1	0.8
	1.1 (-0.6, 2.3)	0.2 (-7.6, 8.7)	1.3 (-5.6, 8.7)	2.2 (-8.3, 13.8)	-7.0 (-18.2, 6.5)	4.7 (-3.3, 13.5)	2.4 (-1.2, 6.2)	-0.8 (-2.5, 1.0)	-0.5 (-3.6, 2.8)	-7.0 (-18.2, 6.5)	-0.2 (-4.1, 3.8)	-5.2 (-18.5, 10.1)	0.9 (-5.5, 7.9)
Turkey	52.3	4.7	4.3	2.2	0.2	2.9	3.7	3.4	17.9	1.3	4.2	**1.0**	**6.4**
	-0.7 (-1.6, 0.1)	0.2 (-3.0, 3.4)	0.1 (-1.4, 1.7)	4.0 (0, 8.2)	4.2 (-1.0, 9.8)	-6.3 (-10.3, -2.1)	0.3 (-2.2, 3.8)	2.4 (-3.0, 7.0)	-4.8 (-6.3, -3.3)	4.2 (-1.0, 9.8)	1.8 (-1.4, 5.2)	**11.1 (3.5, 18.6)**	**4.6 (2.1, 7.2)**
South America													
Brazil	42.4	1.7	4.3	2.2	1.5	1.9	3.3	8.2	3.2	2.9	4.7	**6.0**	2.7
	0.1 (-3.7, 2.9)	-2.2 (-7.8, 3.9)	-3.4 (-12.2, 6.2)	-1.7 (-9.3, 6.9)	-9.2 (-16.9, -0.8)	-4.1 (-12.8, 5.5)	-14.8 (-23.3, -5.5)	1.9 (-4.1, 8.5)	0.8 (-10.4, 13.3)	-9.2 (-16.9, -0.8)	6.8 (-5.3, 17.3)	**8.9 (1.5, 16.6)**	1.8 (-6.9, 11.5)
Chile	42.7	1.5	2.8	2.1	1.2	1.2	2.7	1.5	2.0	2.0	4.4	2.3	25.0
	-0.6 (-4.6, 3.6)	-2.4 (-11.0, 6.8)	-0.7 (-16.1, 18.5)	-1.8 (-10.4, 9.4)	2.6 (-11.0, 17.8)	NA	-2.2 (-11.6, 9.4)	-1.7 (-17.2, 15.4)	-9.8 (-18.5, -0.3)	2.6 (-11.0, 17.8)	-3.5 (-14.6, 9.4)	-1.8 (-15.2, 14.8)	0.2 (-5.0, 5.6)
Colombia	34.9	0.8	4.3	1.5	3.0	0.6	4.5	**2.2**	2.3	1.5	6.9	**2.3**	4.9
	-1.5 (-3.4, 0.5)	-3.2 (-12.5, 6.9)	0.3 (-3.9, 4.7)	1.5 (-6.9, 10.6)	-6.6 (-15.5, 3.1)	-3.6 (-12.4, 6.2)	-1.2 (-6.1, 3.8)	**2.9 (0.5, 5.5)**	-8.3 (-13.7, -2.4)	-6.6 (-15.5, 3.1)	-1.2 (-5.7, 3.5)	**8.2 (3.8, 13.2)**	0.8 (-4.1, 6.1)
Ecuador	**33.0**	0.9	4.3	1.1	0.9	0.5	4.8	1.0	1.6	1.3	6.2	**1.5**	9.1
	**3.3 (1.5, 4.8)**	-4.3 (-12.7, 4.7)	2.5 (-3.8, 9.2)	-4.2 (-16.7, 10.2)	1.0 (-8.4, 11.5)	5.4 (-5.1, 17.8)	0.8 (-5.3, 7.4)	4.6 (-4.7, 15.0)	0 (-9.7, 11.1)	1.0 (-8.4, 11.5)	2.0 (-3.4, 7.9)	**8.2 (1.9, 16.5)**	2.8 (-1.7, 7.4)
Central America													
Costa Rica	**25.8**	0.7	2.5	2.1	0.7	0.5	3.1	1.7	1.4	1.2	3.7	1.8	**6.3**
	**1.7 (0.3, 3.1)**	0.7 (-7.4, 9.7)	1.2 (-1.5, 4.0)	1.0 (-2.5, 4.6)	0.8 (-5.9, 7.9)	-4.5 (-12.3, 7.2)	-3.5 (-9.2, 1.8)	0.9 (-6.4, 7.3)	-1.8 (-10.1, 7.6)	0.8 (-5.9, 7.9)	-0.6 (-4.9, 4.0)	7.5 (-1.9, 18.0)	**4.8 (1.0, 8.9)**
North America													
Canada	55.6	2.5	4.3	3.7	0.3	0.6	4.2	4.6	4.7	7.4	7.4	**5.4**	**10.6**
	0 (-0.3, 0.2)	-1.2 (-2.9, 0.6)	0.2 (-1.3, 1.7)	-0.5 (-1.2, 0.3)	-0.5 (-1.3, 0.3)	-2.9 (-7.0, 1.3)	0.7 (-0.1, 1.5)	0.7 (-0.7, 2.1)	-4.1 (-4.9, -3.3)	-0.5 (-1.3, 0.3)	-0.7 (-1.2, -0.1)	**2.8 (1.7, 4.0)**	**1.6 (0.9, 2.4)**
United States of America	65.9	2.9	4.1	3.8	2.2	0.8	**4.8**	4.9	5.3	9.5	8.5	8.3	**10.7**
	-1.1 (-1.3, -0.9)	-2.3 (-4.4, -0.2)	-0.2 (-1.1, 0.7)	-1.0 (-3.6, 1.7)	-1.6 (-2.4, -0.7)	-4.0 (-5.7, -2.3)	**1.9 (0.5, 2.9)**	-0.4 (-1.7, 1.0)	-4.5 (-5.6, -3.5)	-1.6 (-2.4, -0.7)	-1.7 (-2.4, -0.8)	0.2 (-1.5, 1.6)	**0.7 (0.1, 1.4)**
Europe													
Belarus	47.4	2.5	4.1	3.6	**0.1**	3.3	3.7	7.5	12.2	**2.6**	**3.0**	**0.8**	**4.1**
	0.5 (-0.4, 1.4)	1.5 (-2.2, 5.2)	1.8 (-0.7, 4.3)	0.8 (-1.6, 3.3)	**3.3 (0.6, 6.7)**	-5.1 (-7.0, -3.1)	-0.6 (-2.9, 1.8)	1.5 (-1.9, 6.8)	-2.0 (-3.4, -0.5)	**3.3 (0.6, 6.7)**	**5.3 (2.3, 8.2)**	**6.9 (1.2, 12.9)**	**4.6 (2.1, 7.1)**
Bulgaria	53.8	**4.3**	4.9	2.8	0.1	4.0	**3.0**	**5.7**	14.2	2.5	2.9	0.4	**9.1**
	0 (-0.9, 0.8)	**4.3 (2.5, 6.2)**	0.3 (-2.2, 3.0)	-1.4 (-3.6, 0.9)	2.1 (-0.1, 4.3)	-6.1 (-8.9, -3.2)	**3.6 (0.8, 6.3)**	**4.6 (1.1, 8.2)**	-2.1 (-3.4, -0.9)	2.1 (-0.1, 4.3)	3.9 (-0.5, 8.6)	-0.7 (-9.6, 7.9)	**3.1 (1.4, 4.8)**
Croatia	71.5	4.4	6.6	3.2	0.1	3.6	3.7	8.7	14.8	6.3	4.8	0.8	**14.6**
	-3.6 (-4.5, -1.8)	-2.1 (-4.9, 2.9)	-2.8 (-6.0, 0.5)	0.1 (-3.3, 3.6)	1.2 (-0.8, 3.4)	-9.5 (-14.5, -4.4)	-6.2 (-7.8, -4.5)	-0.2 (-4.4, 4.2)	-8.9 (-10.8, -5.9)	1.2 (-0.8, 3.4)	-0.7 (-4.0, 2.6)	2.6 (-6.0, 11.8)	**3.8 (2.5, 5.1)**
Czech Republic	56.4	2.8	4.4	3.1	0.1	1.9	2.8	6.0	6.9	7.4	**3.9**	**1.6**	15.6
	-1.0 (-1.5, -0.6)	-7.2 (-9.5, -4.8)	-0.4 (-3.6, 1.4)	0.2 (-2.3, 2.7)	2.1 (-0.1, 4.4)	-6.7 (-9.0, -4.4)	0.8 (-1.5, 3.3)	1.7 (-1.0, 4.5)	-8.7 (-9.8, -7.6)	2.1 (-0.1, 4.4)	**1.5 (0.2, 2.9)**	**13.3 (7.5, 19.3)**	0.7 (-1.3, 2.8)
Denmark	**76.5**	4.2	**11.0**	3.1	NA	1.1	4.0	5.2	5.7	**14.4**	5.2	**1.7**	20.9
	**2.1 (1.5, 2.6)**	0.2 (-2.6, 3.1)	**3.9 (1.9, 6.0)**	0.9 (-3.4, 5.4)	NA	-2.4 (-6.3, 1.8)	0.6 (-2.1, 3.4)	-1.3 (-5.3, 2.9)	-4.0 (-6.8, -1.1)	**6.8 (4.8, 8.9)**	0.7 (-1.0, 2.6)	**15.2 (8.5, 22.6)**	1.2 (-0.1, 2.6)
Estonia	45.3	2.4	4.3	3.6	**0.6**	2.1	3.5	3.7	7.6	**4.5**	**4.9**	**2.7**	6.0
	1.2 (-0.9, 3.5)	-0.6 (-9.3, 9.0)	-0.4 (-7.8, 7.5)	4.4 (-3.5, 12.8)	**5.3 (0.9, 10.8)**	-9.6 (-18.8, 0.7)	1.0 (-4.4, 6.8)	4.3 (-5.8, 15.7)	-6.4 (-9.8, -2.8)	**5.3 (0.9, 10.8)**	**8.2 (1.4, 15.5)**	**22.6 (8.3, 38.8)**	2.9 (-3.1, 9.4)
France	74.3	1.6	4.3	4.1	**0.6**	2.3	4.2	**10.5**	13.3	**9.2**	6.6	**3.0**	**14.6**
	-0.8 (-1.3, -0.3)	-6.0 (-8.1, -3.7)	1.5 (-0.6, 3.7)	1.5 (-1.7, 4.8)	**3.8 (1.6, 6.1)**	-5.6 (-11.0, 0.3)	2.2 (-0.1, 4.6)	**2.9 (0.8, 5.8)**	-3.5 (-5.4, -1.5)	**3.8 (1.6, 6.1)**	-0.3 (-1.5, 0.9)	**10.2 (7.7, 12.6)**	**2.1 (0.6, 3.6)**
Germany	69.6	4.3	4.1	3.7	0.4	1.5	4.6	7.6	8.4	8.0	6.1	3.1	18.0
	-0.9 (-1.8, 0)	0.7 (-2.8, 4.4)	0.8 (-4.5, 6.5)	1.7 (-1.5, 5.1)	-1.4 (-5.3, 2.8)	-12.7 (-18.7, -6.0)	1.4 (-3.5, 6.7)	-6.1 (-9.8, -2.3)	-6.0 (-7.8, -4.2)	-1.4 (-5.3, 2.8)	1.0 (-2.0, 4.1)	1.8 (-1.5, 5.3)	1.6 (-0.1, 3.4)
Iceland	57.9	4.4	9.0	3.5	NA	1.9	3.4	3.8	5.2	9.7	4.8	4.3	11.8
	-1.6 (-4.8, 1.8)	-11.1 (-19.6, -1.8)	1.0 (-8.8, 11.7)	6.1 (-5.1, 15.4)	NA	5.2 (-4.8, 16.3)	0.1 (-15.3, 19.5)	-6.5 (-23.0, 12.0)	-5.0 (-13.6, 4.5)	-5.3 (-12.1, 2.1)	-7.1 (-15.2, 1.6)	10.6 (-2.1, 26.3)	0.4 (-7.1, 8.4)
Ireland	**56.8**	2.8	4.5	3.4	0.4	1.0	3.6	**3.3**	4.7	7.5	6.5	**5.2**	14.1
	**2.1 (1.5, 2.6)**	-0.7 (-4.8, 3.6)	-0.9 (-3.1, 1.5)	2.1 (-1.8, 6.4)	1.7 (0, 3.6)	3.1 (-4.6, 11.5)	-0.1 (-2.4, 2.2)	**7.7 (2.0, 13.7)**	0.5 (-3.4, 4.6)	1.7 (0, 3.6)	1.3 (-1.7, 4.4)	**12.1 (8.4, 16.0)**	2.0 (-0.4, 4.4)
Italy	65.9	6.6	4.3	4.7	**1.3**	1.3	4.1	3.8	6.2	**10.7**	8.7	**1.8**	**12.5**
	0.8 (-0.5, 2.1)	-2.0 (-3.9, 0.1)	-2.2 (-5.2, 0.9)	0.6 (-5.2, 6.6)	**4.0 (2.7, 5.2)**	-5.4 (-9.2, -1.3)	-2.5 (-6.8, 2.0)	-0.5 (-6.1, 5.6)	-3.9 (-7.4, -0.2)	**4.0 (2.7, 5.2)**	2.0 (-0.9, 5.0)	**11.4 (3.8, 19.7)**	**3.0 (1.5, 4.5)**
Latvia	**42.5**	3.1	5.5	2.8	0.2	2.0	3.0	4.1	9.4	2.7	3.3	**1.2**	5.3
	**3.1 (1.8, 4.5)**	2.8 (-1.2, 7.1)	2.6 (-3.9, 9.5)	3.9 (-1.9, 10.2)	8.9 (-4.6, 20.2)	-0.2 (-3.0, 2.8)	0.4 (-3.0, 4.1)	2.4 (-6.9, 13.1)	-2.5 (-5.6, 0.7)	8.9 (-4.6, 20.2)	5.1 (-0.2, 10.7)	**10.3 (0.9, 17.2)**	2.3 (-3.1, 7.9)
Lithuania	45.8	2.2	5.0	2.9	**0.1**	2.4	3.7	5.7	9.1	**3.4**	4.0	**2.9**	4.6
	-0.3 (-1.8, 1.4)	-5.4 (-10.2, -0.1)	0.2 (-4.4, 4.1)	2.2 (-1.4, 6.5)	**3.9 (0.8, 7.1)**	-3.2 (-9.5, 3.4)	-1.1 (-4.6, 2.5)	1.3 (-3.8, 6.9)	-5.1 (-6.8, -3.2)	**3.9 (0.8, 7.1)**	1.8 (-1.6, 5.5)	**14.8 (7.5, 23.3)**	1.6 (-2.2, 5.3)
Malta	44.9	1.6	3.9	4.0	NA	1.6	4.4	4.8	4.5	**3.6**	7.0	1.5	10.1
	1.7 (-1.3, 4.8)	-1.2 (-10.4, 9.1)	-0.4 (-13.2, 14.3)	2.0 (-7.1, 9.8)	NA	2.5 (-6.6, 12.6)	-6.9 (-15.9, 3.2)	5.6 (-0.7, 12.4)	-7.8 (-18.6, 5.5)	**10.4 (1.6, 20.1)**	8.9 (-5.2, 25.1)	-4.5 (-14.1, 5.9)	4.6 (-5.6, 15.7)
Netherlands	**61.4**	1.4	4.5	3.9	**0.7**	0.9	3.8	3.6	6.4	**12.4**	6.2	**1.7**	**16.0**
	**1.7 (1.4, 2.1)**	-0.6 (-3.3, 2.1)	0.4 (-1.3, 2.2)	0.3 (-2.0, 2.6)	**4.3 (3.3, 5.3)**	-5.7 (-8.1, -3.2)	0.4 (-1.1, 1.9)	-0.4 (-2.1, 1.4)	-2.3 (-3.6, -1.1)	**4.3 (3.3, 5.3)**	1.6 (-0.5, 3.6)	**6.3 (1.1, 11.7)**	**3.8 (3.2, 4.4)**
Norway	**71.2**	3.3	**10.8**	3.8	NA	0.4	3.5	**2.8**	4.3	**10.7**	5.2	**3.0**	23.2
	**1.1 (0.4, 1.9)**	1.8 (-1.0, 4.7)	**2.4 (0.2, 4.6)**	1.2 (-2.2, 4.6)	NA	-9.5 (-22.5, 5.8)	2.0 (-3.4, 7.8)	**4.5 (0.7, 8.5)**	-4.2 (-6.7, -1.6)	**2.7 (1.4, 3.9)**	-0.8 (-2.5, 1.0)	**8.9 (6.1, 11.8)**	1.0 (-1.2, 3.3)
Poland	46.4	3.2	5.6	3.2	NA	2.1	2.8	4.1	11.2	3.0	3.3	0.9	7.0
	-2.3 (-3.8, -0.5)	-0.7 (-8.2, 6.6)	-4.1 (-7.4, -0.5)	-3.4 (-8.4, 2.1)	NA	-6.6 (-14.0, -1.8)	4.9 (-4.6, 15.6)	0.5 (-12.8, 15.8)	-7.5 (-10.0, -4.9)	-0.2 (-5.7, 5.6)	3.9 (-4.0, 12.8)	2.4 (-7.6, 13.5)	4.7 (-0.3, 10.0)
Slovakia	64.5	2.9	**4.5**	2.8	0.1	3.0	3.6	13.1	8.8	5.2	3.8	**0.8**	**15.8**
	-1.0 (-1.7, -0.4)	-0.4 (-4.7, 4.3)	**4.2 (0.3, 8.1)**	2.0 (-2.9, 7.1)	2.1 (-1.5, 5.9)	-6.4 (-10.4, -2.1)	0.6 (-2.9, 4.3)	1.4 (-4.8, 8.2)	-6.2 (-7.6, -4.7)	2.1 (-1.5, 5.9)	3.0 (-2.1, 8.4)	**8.7 (3.1, 14.8)**	**2.5 (0.4, 4.6)**
Slovenia	66.6	1.3	3.9	3.0	**0.3**	2.1	3.1	8.3	9.8	**9.8**	4.5	**2.0**	18.8
	-0.9 (-2.3, 0.6)	-4.8 (-16.1, 8.3)	-1.4 (-10.4, 10.1)	-2.1 (-13.4, 10.4)	**4.3 (0.8, 8.1)**	-8.3 (-14.7, -1.1)	-7.4 (-13.3, 0.9)	-0.7 (-7.0, 5.9)	-6.4 (-11.0, -1.6)	**4.3 (0.8, 8.1)**	-0.8 (-4.4, 3.0)	**16.2 (11.1, 21.9)**	0.3 (-2.7, 3.4)
Spain	65.2	6.7	3.9	4.2	1.0	3.8	3.7	7.7	11.6	5.1	7.5	**1.8**	**8.3**
	-1.3 (-3.2, 0.6)	-2.5 (-5.9, 1.0)	1.5 (-1.2, 4.4)	0.9 (-4.3, 6.4)	2.2 (-1.0, 5.7)	-9.8 (-13.8, -5.6)	-0.4 (-2.4, 1.8)	-3.4 (-8.4, -1.8)	-5.7 (-7.2, -4.1)	2.2 (-1.0, 5.7)	0.4 (-1.7, 2.6)	**7.7 (2.7, 13.1)**	**6.3 (5.0, 7.1)**
Sweden	**55.8**	2.7	10.0	2.6	NA	0.3	3.5	2.8	2.5	**10.3**	4.1	**3.3**	**13.7**
	**1.5 (1.0, 2.0)**	-2.5 (-5.9, 1.0)	1.1 (-0.7, 2.8)	-0.5 (-3.1, 2.1)	NA	-3.6 (-12.2, 6.1)	2.5 (-0.8, 5.9)	0.8 (-1.2, 2.8)	-3.3 (-5.5, -1.0)	**3.7 (1.8, 5.7)**	0.8 (-2.5, 4.1)	**6.1 (3.4, 9.4)**	**1.8 (0.6, 3.0)**
Switzerland	71.7	**3.1**	4.3	4.6	0.8	1.2	4.1	5.6	6.3	13.9	6.8	2.3	18.5
	-0.2 (-1.0, 0.6)	**4.4 (0.4, 8.5)**	1.1 (-0.4, 3.1)	3.0 (-1.6, 7.8)	0.8 (-1.3, 3.1)	-5.0 (-14.4, 4.7)	1.1 (-2.0, 4.5)	0.7 (-3.7, 5.4)	-4.0 (-7.8, 0)	0.8 (-1.3, 3.1)	0 (-3.1, 3.2)	7.8 (0, 16.3)	0.2 (-1.3, 1.8)
Ukraine	45.4	2.9	4.4	3.2	**0.1**	3.0	3.2	6.5	11.2	**3.2**	**3.1**	0.7	**3.8**
	-0.3 (-0.7, 0.1)	0.7 (-1.3, 2.7)	-0.2 (-2.5, 2.2)	0.6 (-1.3, 2.4)	**3.6 (1.9, 4.9)**	-3.0 (-4.1, -2.2)	-1.4 (-3.9, 1.1)	0.8 (-0.9, 2.6)	-3.4 (-4.2, -2.6)	**3.6 (1.9, 4.9)**	**3.4 (1.5, 5.4)**	3.2 (-0.7, 7.2)	**2.2 (1.1, 3.2)**
United Kingdom – England and Wales	**52.5**	1.3	4.2	3.8	**0.6**	0.8	3.7	**4.4**	4.3	**8.2**	**6.1**	**2.2**	**13.2**
	**0.9 (0.5, 1.5)**	-3.2 (-4.6, -1.1)	-1.0 (-1.8, -0.3)	0.1 (-0.6, 1.3)	**2.9 (1.6, 4.1)**	-3.3 (-4.7, -1.1)	0.3 (-1.3, 2.0)	**2.7 (1.5, 4.0)**	-2.5 (-3.4, -1.1)	**2.9 (1.6, 4.1)**	**1.2 (0.6, 2.3)**	**8.9 (7.2, 10.7)**	**0.9 (0.2, 1.5)**
United Kingdom – Northern Ireland	54.8	3.7	5.1	3.3	0.4	1.1	3.0	**4.0**	4.5	7.7	5.3	**2.4**	14.5
	0.8 (-0.3, 1.9)	0.2 (-8.1, 9.2)	-1.7 (-5.5, 2.3)	2.0 (-4.6, 9.3)	-0.9 (-4.3, 2.5)	2.4 (-7.1, 13.2)	0.8 (-3.6, 5.5)	**9.2 (3.2, 15.7)**	-1.1 (-5.0, 3.0)	-0.9 (-4.3, 2.5)	0.4 (-3.2, 4.1)	**15.9 (7.9, 24.5)**	0.5 (-2.5, 3.5)
United Kingdom - Scotland	60.6	3.9	4.2	4.3	**0.3**	1.2	3.6	4.9	5.7	**8.7**	6.2	1.6	16.0
	0.3 (-0.7, 1.2)	2.1 (-2.4, 5.5)	-2.1 (-4.7, 2.8)	-0.6 (-1.6, 0.5)	**1.7 (0.2, 3.2)**	-2.8 (-6.7, 1.2)	0.5 (-2.3, 3.4)	4.8 (0, 9.9)	-1.1 (-3.7, 1.6)	**1.7 (0.2, 3.2)**	0.7 (-0.7, 2.1)	4.2 (-4.8, 13.9)	-0.9 (-2.6, 0.8)
Oceania													
Australia	82.5	1.1	**4.6**	**3.3**	0.5	0.6	4.9	8.8	4.5	26.6	7.7	**7.1**	**12.9**
	0.3 (0, 0.7)	-1.8 (-4.3, 0.9)	**1.3 (0.5, 2.1)**	**1.5 (0.5, 2.6)**	-2.2 (-3.0, -1.5)	-2.3 (-4.6, -0.3)	1.2 (-0.2, 2.6)	0.5 (-0.6, 1.5)	-0.4 (-2.3, 1.6)	-2.2 (-3.0, -1.5)	-0.4 (-1.4, 0.5)	**12.8 (10.1, 15.8)**	**0.9 (0.2, 1.6)**
New Zealand	70.0	1.4	4.6	2.9	0.2	0.4	4.4	4.6	3.8	21.5	6.5	5.1	14.3
	-1.4 (-2.2, -0.4)	-6.8 (-13.8, -1.3)	0.1 (-2.4, 2.8)	-0.7 (-3.6, 2.3)	-3.9 (-5.7, -1.8)	2.6 (-5.5, 11.5)	-1.5 (-4.4, 1.7)	0.1 (-2.7, 3.0)	-2.2 (-4.7, 0.3)	-3.9 (-5.7, -1.8)	-2.9 (-5.4, -0.5)	3.8 (-0.7, 8.4)	-0.2 (-2.7, 2.4)

[*] Average age-standardized cancer incidence rates (ASRs) between 2000 and 2012 were calculated.
[#] Average annual percentage changes (AAPCs) with 95% confidence intervals (CIs) in cancer incidence among 20-49-year-old adults during the period of 2000-2012 were calculated using the Joinpoint Regression Program (version 4.9.0.1). A maximum of two joinpoints were permitted in this analysis.
Bold numbers denote statistically significant positive AAPCs.
Abbreviations: AAPC, Average annual percentage change; ASR, age-standardized cancer incidence rate; CI, confidence interval; CNS, central nervous system.

Supplementary Table S6. Sensitivity analyses of incidence trends of early-onset obesity-related cancers and non-obesity-related cancers by regions in 2000-2010.

Region	All obesity-related cancers	All obesity-related cancers excluding thyroid cancer	All obesity-related cancers excluding stomach cancer	All obesity-related cancers excluding oesophagus cancer	All non-obesity-related cancers	All non-obesity-related cancers excluding prostate cancer
	ASR/100,000* AAPC (95% CI)#	ASR/100,000 AAPC (95% CI)	ASR/100,000 AAPC (95% CI)	ASR/100,000 AAPC (95% CI)	ASR/100,000 AAPC (95% CI)	ASR/100,000 AAPC (95% CI)
Female						
All countries combined	**42.4**	**27.0**	**39.0**	**42.1**	**97.2**	
	**4.3 (4.1, 4.6)**	**0.9 (0.7, 1.0)**	**4.8 (4.5, 5.0)**	**4.4 (4.1, 4.7)**	**0.8 (0.7, 1.0)**	
Africa	21.6	20.0	19.2	19.7	125.5	
	3.0 (-2.2, 8.6)	2.3 (-3.1, 8.1)	4.9 (-0.7, 10.7)	2.0 (-4.3, 8.7)	-2.1 (-4.0, -0.1)	
Asia	**60.4**	**31.5**	**52.2**	**54.4**	29.2	
	**9.2 (8.8, 9.7)**	**1.1 (0.8, 1.4)**	**11.0 (10.7, 11.5)**	**10.0 (9.7, 10.4)**	0.3 (-0.1, 0.7)	
South America	39.5	24.3	**34.2**	**39.3**	75.4	
	3.0 (-0.1, 6.7)	0.8 (-0.9, 2.7)	**3.3 (1.3, 5.7)**	**3.1 (0.1, 6.5)**	-1.7 (-2.7, -0.7)	
Central America	**40.6**	22.3	**34.5**	**40.5**	102.4	
	**3.3 (1.4, 5.2)**	-0.5 (-3.0, 2.0)	**4.2 (1.9, 6.6)**	**3.2 (1.3, 5.2)**	-0.3 (-0.6, 0)	
North America	**48.3**	**28.6**	**46.8**	**41.1**	102.9	
	**3.4 (2.9, 3.8)**	**1.7 (1.5, 1.9)**	**3.4 (2.9, 3.8)**	**3.6 (3.3, 4.0)**	0 (-0.3, 0.4)	
Europe	**32.8**	24.6	**30.8**	**32.5**	**102.5**	
	**1.7 (1.4, 2.1)**	0.3 (0, 0.7)	**2.0 (1.6, 2.4)**	**1.7 (1.4, 2.1)**	**1.1 (0.9, 1.2)**	
Oceania	**38.7**	**26.2**	**37.2**	**38.5**	123.7	
	**2.3 (1.9, 2.7)**	**1.4 (0.7, 2.0)**	**2.4 (1.9, 2.9)**	**2.3 (1.9, 2.7)**	-0.2 (-0.5, 0.2)	
Male						
All countries combined	**27.9**	**24.5**	**23.3**	**26.6**	**53.5**	50.6
	**1.4 (1.2, 1.7)**	**0.3 (0.1, 0.5)**	**2.1 (1.9, 2.4)**	**1.5 (1.3, 1.9)**	**0.2 (0.1, 0.4)**	-0.2 (-0.3, 0)
Africa	**19.9**	**19.4**	**17.9**	14.1	62.7	61.1
	**4.8 (0.2, 9.6)**	**4.9 (0.1, 9.9)**	**5.5 (0.8, 10.5)**	3.2 (-4.4, 11.3)	-0.6 (-3.2, 2.1)	-0.5 (-3.1, 2.2)
Asia	**43.4**	37.8	**42.4**	**42.3**	**31.2**	30.8
	**2.1 (1.8, 2.5)**	-0.4 (-1.0, 0.2)	**3.6 (3.3, 3.9)**	**2.3 (1.9, 2.7)**	**0.4 (0.1, 0.7)**	0.2 (0, 0.5)
South America	22.1	19.5	**15.0**	21.1	36.7	33.9
	0.7 (-1.5, 2.9)	0 (-2.4, 2.5)	**2.3 (0.1, 4.6)**	1.1 (-1.1, 3.5)	-0.1 (-0.8, 0.6)	-0.7 (-2.2, 0.9)
Central America	18.1	15.5	11.8	17.9	**25.9**	**24,1**
	-0.3 (-1.6, 1.1)	-1.8 (-3.5, -0.2)	1.5 (-0.3, 3.3)	-0.1 (-1.5, 1.3)	**2.1 (0.5, 3.5)**	**1.5 (0.1, 3.0)**
North America	**26.3**	**21.9**	**24.4**	**25.4**	62.2	55.1
	**2.0 (1.4, 2.7)**	**1.4 (0.9, 1.9)**	**2.2 (1.7, 2.7)**	**2.1 (1.5, 2.6)**	-0.2 (-0.6, 0.3)	-0.6 (-0.9, -0.2)
Europe	**22.2**	**20.1**	**19.2**	**20.6**	**57.4**	55.6
	**1.0 (0.7, 1.2)**	**0.5 (0.3, 0.7)**	**1.5 (1.1, 1.8)**	**1.1 (0.9, 1.4)**	**0.3 (0.1, 0.5)**	0 (-0.2, 0.2)
Oceania	**25.9**	**22.6**	**23.9**	**24.8**	80.5	74.4
	**2.1 (1.7, 2.6)**	**1.7 (1.2, 2.4)**	**2.3 (1.9, 2.9)**	**2.2 (1.7, 2.7)**	0.3 (-0.4, 0.9)	-0.8 (-1.1, -0.4)

* Average age-standardized cancer incidence rates (ASRs) between 2000 and 2010 were calculated.
\# Average annual percentage changes (AAPCs) with 95% confidence intervals (CIs) in cancer incidence among 20-49-year-old adults during the period of 2000-2010 were calculated using the Joinpoint Regression Program (version 4.9.0.1). A maximum of two joinpoints were permitted in this analysis.
Bold numbers denote statistically significant positive AAPCs.
Abbreviations: AAPC, Average annual percentage change; ASR, age-standardized cancer incidence rate; CI, confidence interval.
